# Forest Heterogeneity by Chain Saw: How Between‐Patch Variation in Old Growth Attributes Changes the Metacommunities of Beetles

**DOI:** 10.1111/ele.70355

**Published:** 2026-03-05

**Authors:** Oliver Mitesser, Marc W. Cadotte, Akira S. Mori, Fons van der Plas, Anne Chao, Julia Rothacher, Claus Bässler, Mirjana Bevanda, Peter H. W. Biedermann, Pia Bradler, Antonio Castañeda‐Gómez, Orsi Decker, Benjamin M. Delory, Sebastian Dittrich, Heike Feldhaar, Andreas Fichtner, Alexander Kreis, Lisa Köstler‐Albert, Ludwig Lettenmaier, Goddert von Oheimb, Luisa Pflumm, Kerstin Pierick, Jakob Schwalb‐Willmann, Simon Thorn, Leah Vogelfänger, Wolfgang Weisser, Martin Wegmann, Clara Wild, Jörg Müller

**Affiliations:** ^1^ Chair of Conservation Biology and Forest Ecology, Biocenter Julius‐Maximilians‐Universität Würzburg Rauhenebrach Germany; ^2^ Biological Sciences University of Toronto‐Scarborough Toronto Ontario Canada; ^3^ Research Center for Advanced Science and Technology University of Tokyo Tokyo Japan; ^4^ Plant Ecology and Nature Conservation Wageningen University Wageningen the Netherlands; ^5^ Institute of Statistics National Tsing Hua University Hsin‐Chu Taiwan; ^6^ University of Bayreuth Bayreuth Germany; ^7^ Earth Observation Research Cluster, Department of Remote Sensing University of Würzburg Würzburg Germany; ^8^ Forest Entomology and Forest Protection Albert Ludwig University of Freiburg Freiburg im Breisgau Germany; ^9^ Institute of Ecology Leuphana University Lüneburg Germany; ^10^ Institute of General Ecology and Environmental Protection TUD Dresden University of Technology Tharandt Germany; ^11^ Environmental Sciences Group, Copernicus Institute of Sustainable Development Utrecht University Utrecht the Netherlands; ^12^ Animal Population Ecology, Bayreuth Center for Ecology and Environmental Research (BayCEER) University Bayreuth Bayreuth Germany; ^13^ Spatial Structures and Digitization of Forests/Silviculture and Forest Ecology of the Temperate Zones University of Göttingen Göttingen Germany; ^14^ Philipps Universität Marburg, Applied Ecology Marburg Germany; ^15^ Hessian Agency for Nature Conservation, Environment and Geology State Institute for the Protection of Birds Gießen Germany; ^16^ Czech Academy of Sciences, Biology Centre Institute of Entomology České Budějovice Czech Republic; ^17^ Forest Zoology TUD Dresden University of Technology Tharandt Germany; ^18^ Terrestrial Ecology Research Group, Department of Ecology and Ecosystem Management, School of Life Sciences Weihenstephan Technical University of Munich Freising Germany; ^19^ Bavarian Forest National Park Grafenau Germany

**Keywords:** beetles, coleoptera, diversity, metacommunity paradigms, temperate forests

## Abstract

Metacommunity theory has expanded our understanding of how spatial dynamics and local interactions influence species communities. Different assembly archetypes, reflecting different roles of species differences, habitat differences, and dispersal have been described, but we lack empirical studies specifically in terrestrial habitats testing which archetype is most important. In a replicated design, we experimentally enhanced structural between‐patch heterogeneity in homogeneous production forests and developed a statistical framework controlling for sample incompleteness to detect different metacommunity processes. Meta‐analyses on > 100 K individuals of > 1.3 K beetle species showed an increase of ~60 species in heterogenized forests at γ‐level promoted by increasing α‐diversity consistent with the *mass‐effect* and an increase of β‐diversity by ~10% supporting *species‐sorting*. Additionally, we tested β‐deviations from random assembly as a proxy of *neutral processes*. Findings indicate that enhancing structural heterogeneity can shift forests from *patch‐dynamics* dominance towards *mass‐effect* and *species‐sorting*, offering a promising pathway to restore biodiversity in managed landscapes.

## Graphical abstract

Metacommunity theory has expanded our understanding of how spatial dynamics and local interactions influence species communities, but we lack empirical studies specifically in terrestrial habitats testing which archetype is most important. In a replicated design we experimentally enhanced structural between‐patch heterogeneity in homogeneous production forests and developed a statistical framework controlling for sample incompleteness to detect different metacommunity processes. Meta‐analyses on > 100 K individuals of > 1.3 K beetle species showed an increase of ~60 species in heterogenized forests at γ‐level promoted by increasing α‐diversity consistent with the mass‐effect and an increase of β‐diversity by ~10% supporting species‐sorting.

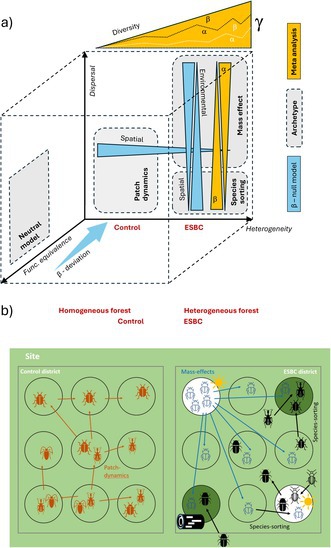

## 1 Introduction

During the last two decades, metacommunity theory has expanded our understanding of how spatial dynamics and local interactions influence the structure and diversity of species assemblages (Leibold et al. 2004; Cottenie 2005; Gonzalez 2009; Record et al. 2021). Within this framework, four archetypes that characterise metacommunities are recognised (Leibold et al. 2004; Logue et al. 2011; Leibold and Chase 2018). Each of them predicts different assembly mechanisms based on dispersal rates and differences in habitats and species: The *patch‐dynamics* archetype assumes homogeneous patches which are occupied by species with a trade‐off between dispersal and competitive ability. Superior competitors can displace others in occupied patches, whereas weaker competitors persist at the regional level by rapidly colonising newly available patches. Variation in species composition is due to processes of colonisation and extinction. The *species‐sorting* archetype assumes that habitat patches are heterogeneous, and dispersal rates are relatively low, allowing local competitive dynamics to determine community composition. Local community composition is primarily shaped by a match between environmental conditions and the ecological niches of the species. A high level of environmental variation across the landscape thus leads to high regional species richness through different species assemblages in different patches. The *mass‐effect* archetype assumes that heterogeneous habitat patches are interconnected, and dispersal rates are high. This allows species to reproduce in source patches and frequent dispersal allows them to also exist in sink patches where local conditions prevent long‐term survival or reproduction. High dispersal over long distances might override signals of species‐niche matching while increasing local diversity. Finally, the *neutral* archetype assumes ecological equivalence among individuals, such that per‐capita probabilities of birth, death, and dispersal do not differ among species. Under such conditions, community composition changes through stochastic demographic processes (ecological drift) and dispersal, rather than deterministic niche‐based sorting (Hubbell 2001).

**FIGURE 1:**
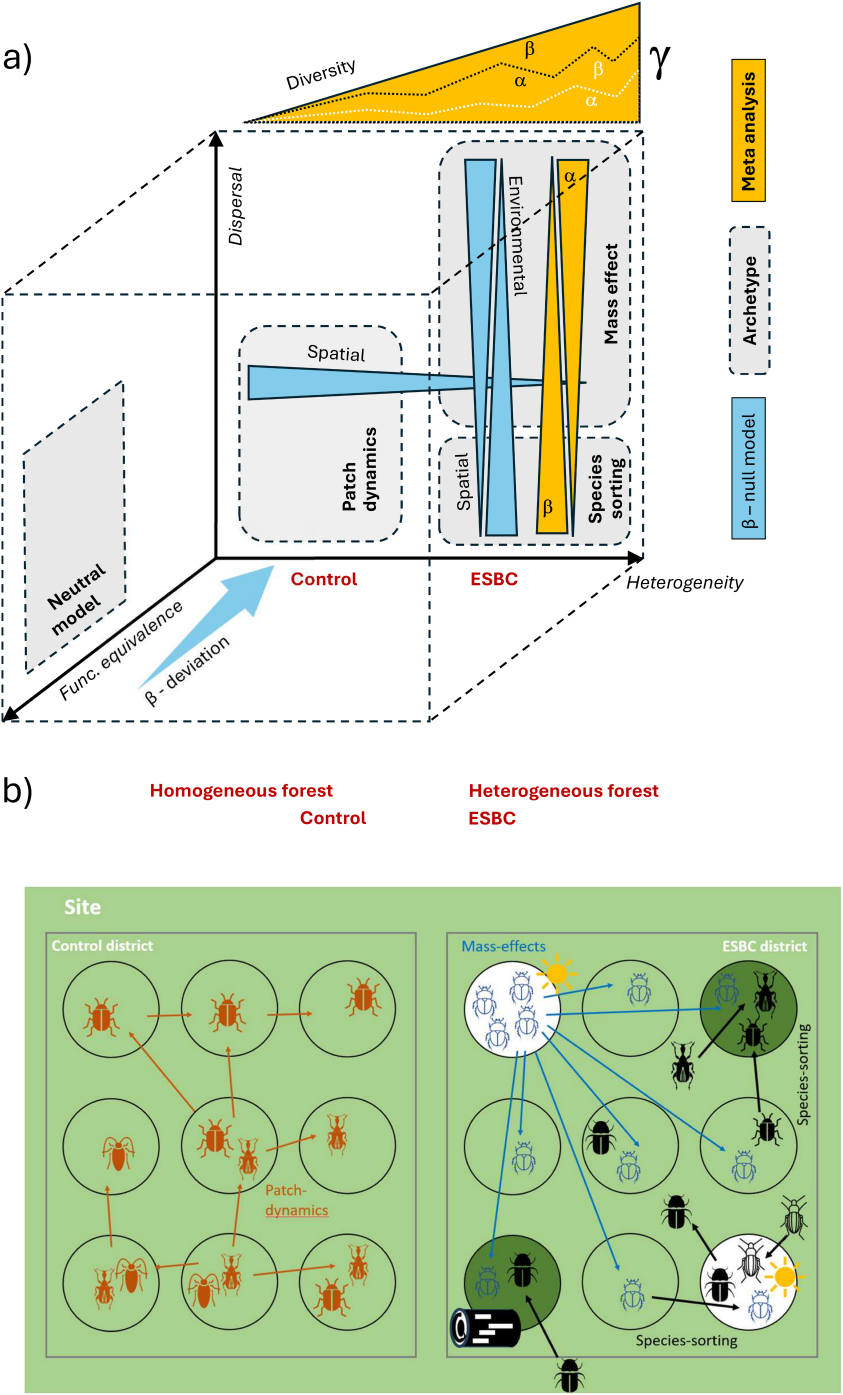
(a) Extension of Logue et al. (2011)'s scheme of metacommunity mechanisms based on the dimensions of heterogeneity, functional equivalence of individuals, and dispersal. Metacommunity archetypes of *species‐sorting*, *mass‐effect*, *patch‐dynamics*, and *neutral processes* occupy typical areas in the diagram. Both meta‐analyses (yellow) and β‐deviation analysis (blue) allow discrimination between the processes. Dotted black and white lines within the hypothetical increase of γ‐diversity (yellow triangle at the top) indicate different hypothetical allocation of the effect to α‐ and β‐diversity. (b) Conceptual scheme of archetypes from metacommunity theory explaining diversity and assembly mechanisms in local beetle communities in homogeneous and heterogenous forests. White and green colours indicate habitat differences. In ESBC patches light and dark beetle symbols indicate matching of beetles' attributes to light and dark green habitats, respectively. Patch dynamics in the homogeneous forest (on the left) result in more similar communities in patches closer to each other while species sorting between the patches of a heterogeneous forest (on the right) populates patches by species from the surrounding landscape (from inside and outside the other patches) according to their attribute match. In addition, suitable habitat can constitute source patches flooding the forest with specific species (mass effect). This might result in an overall increase from 3 (on the left) to 4 species (on the right).

Theoretical studies have shown that the different metacommunity archetypes make different predictions on how biodiversity is distributed across space and which predictors explain the patterns (Thompson et al. 2020). While processes of all archetypes have been demonstrated to matter, these relationships can be utilised to identify the most influential mechanisms in real‐world landscapes (see hypotheses below). Despite the theoretical progress in metacommunity theory, it is still insufficiently linked to empirical data, particularly in terrestrial habitats (Logue et al. 2011), where soft boundaries between patches with gradual transitions of resources, community composition, and processes are the rule rather than the exception. To disentangle the importance of different metacommunity archetypes, Logue et al. (2011) proposed developing an integrative framework that combines new experimental approaches and novel statistical methods and is based on three key axes: species differences (functional equivalence vs. differentiation), habitat differences (homogeneity vs. heterogeneity), and dispersal dynamics (Figure 1a).

We selected homogeneous production forests as a model system to implement a novel large‐scale experimental approach (Müller et al. 2023) aimed at identifying the dominant processes driving metacommunity assembly and assessing how their relative importance varies with landscape heterogeneity. At the beginning of the experiment, we manipulated the between‐forest‐patch heterogeneity by creating gaps and deadwood towards old‐growth features by chainsaw and selected homogeneous control forests.

We developed a new meta‐analysis approach to disentangle the effects of within‐patch α‐ and between‐patch β‐diversity on γ‐diversity at the landscape scale while accounting for sample incompleteness in empirical data for taxonomic (TD) and functional diversity (FD) along Hill numbers to capture differences in species abundances and ecological attributes (Chao et al. 2023; Masso et al. 2025). Hill numbers refer to a generalised framework that sums the weighted exponential proportional species abundances. Specifically, a Hill exponent of 0 means that species contribute to estimated diversity regardless of their frequency (i.e., species richness), while exponents > 1 give more weight to more frequent species. As they can easily be transformed to traditional metrics like Shannon entropy or the Simpson index (Chao et al. 2014), they relate this study to former analyses of different diversity measures adopted by ecologists for decades.

Using the same framework, we were able to estimate pairwise β‐deviations between patches of a forest compared to randomised communities, while accounting for unobserved species. Employing estimates of unobserved species addresses well‐documented artefacts in β‐diversity differences by under‐sampling (Tuomisto 2010; Tucker et al. 2016). Subtraction of null‐model β‐diversity in general controls for effects of demographic and dispersal stochasticity on the β‐diversity pattern expected from the *neutral* archetype (Tucker et al. 2016). Linking β‐deviation to spatial and environmental distances, which are independent predictors due to the experimental design of our study, offered the option to compare the relevance of the structural heterogenization with the effect of space.

Considering multiple dimensions of biodiversity (here facet: TD and FD; scale: α, β, γ; Hill exponent: *q* = 0, 1, 2) including β‐deviation and different predictors enabled the investigation of underlying processes structuring species assemblages. Note that not every conceivable combination of these biodiversity dimensions is informative for each archetype. Thus, we selected a specific subset for each of them to complement the core analysis of taxonomic diversity. In homogeneous forests, *patch‐dynamics* would be indicated by a strong spatial response of β‐deviation (Logue et al. 2011) and is expected to be most pronounced when diversity measures are weighted to prioritise more abundant species (*q* > 0), because a key component of successful dispersal is a sufficient number of dispersers (Fronhofer et al. 2012). An increase of taxonomic γ‐diversity via increasing α‐diversity in heterogeneous forests should indicate *mass‐effects* which would be supported further if pronounced in more abundant species (*q* > 0). An increase of β‐diversity in heterogeneous forests alone would already be a hint on *species‐sorting*. However, β‐diversity might also be affected by *neutral* processes; hence, it has been generally questioned if *species‐sorting* can be derived from responses just in β‐diversity (Cadotte and Tucker 2017). Accounting for neutral processes by a null model approach and expanding the framework to FD based on traits related to dispersal and known as responsive to the enhanced heterogeneity treatment in our study opens the avenue for inferring whether increased heterogeneity also leads to an increased importance of *species‐sorting* (Cadotte and Tucker 2017). A response to structural heterogenization in functional β‐diversity would confirm the selection of relevant traits and point to the high relevance of the axis of functional equivalence in Logue et al. (2011)'s scheme of metacommunity mechanisms (Figure 1a). Vanishing effects in FD would hint at traits and linked mechanisms other than those already included in FD to explain a taxonomic effect. In addition, finding a positive statistical response in functional β‐deviation to environmental heterogeneity only in experimentally heterogenized forests would indicate a genuine response to the treatment via *species‐sorting* (Jeliazkov and Chase 2024).

We used beetles, one of the taxonomically and functionally most diverse insect orders, as a model system (Farrell 1998, see Supplement I1). According to theory (Logue et al. 2011), we expected a shift from metacommunities dominated by *patch‐dynamics* in homogeneous control forests to metacommunities structured by *species‐sorting* and *mass‐effects*, which gives rise to several hypotheses about scale‐dependent biodiversity responses (α, β, and γ) and β‐deviation. We first test the hypothesis (H1) that enhancement of between‐patch heterogeneity increases γ‐diversity of a forest. We expect γ‐diversity to increase (i) via higher β‐diversity according to *species‐sorting* or (ii) via higher α‐diversity if resource‐enriched source patches promote the *mass‐effect*; in the latter case, increased dispersal could homogenise communities and thus reduce β‐diversity (Mouquet and Loreau 2003) (Figure 1). Secondly, we hypothesise (H2) that β‐deviation is better explained by spatial distance in homogeneous forests because other environmental attributes as potential alternative predictors have only low variation. In heterogeneous forests where *species‐sorting* can increase the correlation between communities and the environment, we hypothesise (H3) environmental variation to be the better predictor of β‐deviation. As species differences in habitat requirements and in mobility affect *species‐sorting* and dispersal, we expect effects of habitat difference in heterogeneous forests specifically on functional β‐deviation and a significant explanatory power of spatial distance between patches when taking abundance into account (*q* > 0).

Our results demonstrate an increase in α‐, β‐, and γ‐diversity in response to ESBC treatment and suggest that metacommunities in homogeneous forests are dominated by *patch‐dynamics*, while those in heterogeneous forests by *mass‐effect* and *species‐sorting*.

## 2 Materials and Methods

### 2.1 Study Area and Experimental Design

**FIGURE 2:**
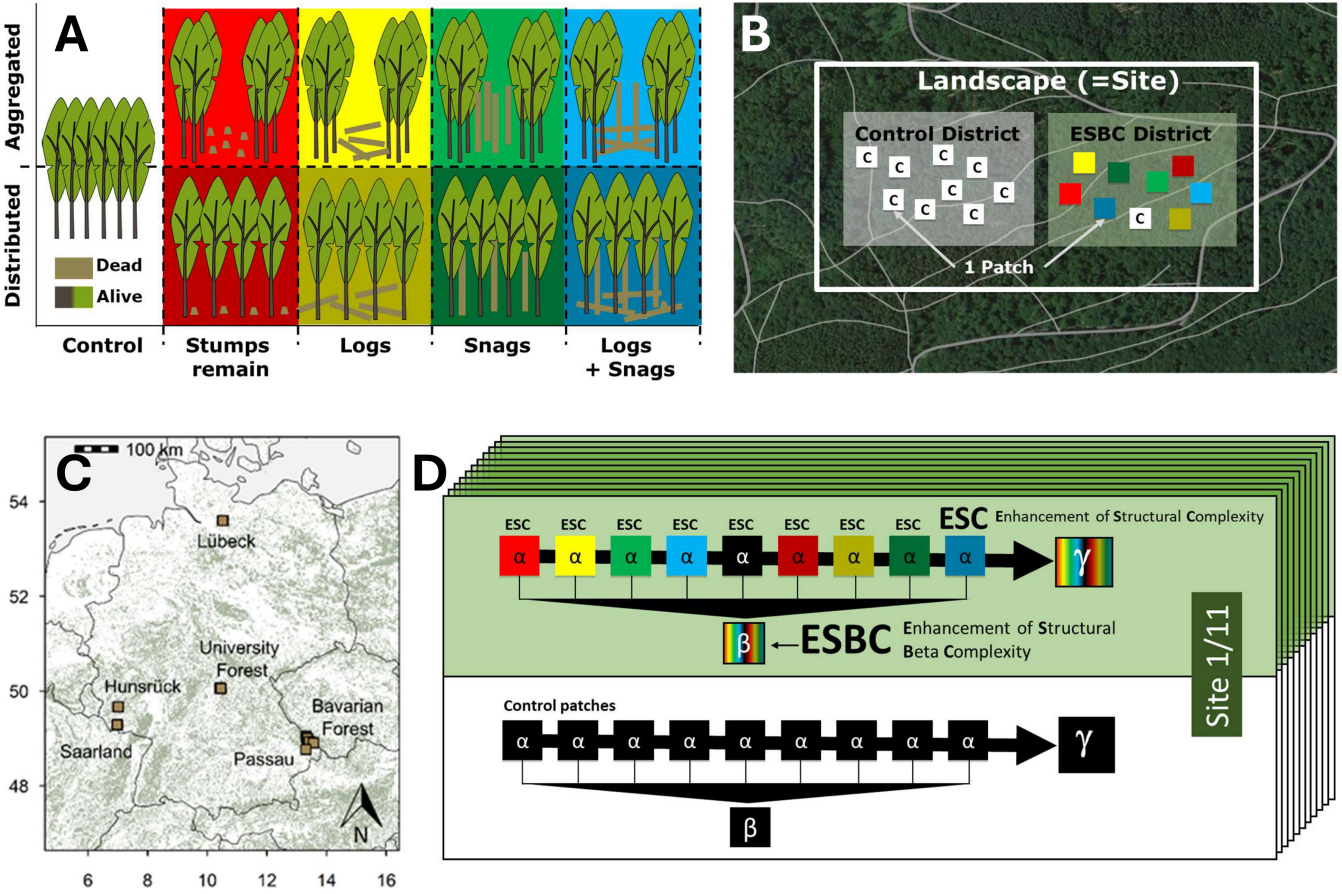
Design of replicated manipulation of habitat heterogeneity in 11 homogeneous production forests and their 11 control forests. (A) Interventions were either aggregated in the centre of the patch (upper row) or evenly distributed over the patch area (lower row). (B) Site with treatment (ESBC: Enhancement of Structural Beta Complexity) district and control district. (C) Geographical distribution of the 11 sites. (D) Schematic representation of the ecosystem elements site, district, and patch in relation to derived diversity levels for an exemplary site.

We established a pairwise design of comparable districts at 11 sites throughout Germany covering a wide range of climate (c. 50–1100 m a.s.l.) and soil conditions (acidic to calcareous) in European beech ( *Fagus sylvatica* ) mixed forests (Müller et al. 2023). Each site comprised a district experimentally enhanced in structural beta complexity (ESBC) and a homogeneous control district. In total, the study included 22 districts of ~20 ha. Within each district a set of 9 or 15 patches was established, with a spatial extent of 50 m × 50 m in a randomised block design. The interventions in ESBC districts aimed to enhance the variation in canopy density and deadwood structures. Forest gaps were created with a diameter of 30 m similar to the average patch size in a typical equilibrium landscape of about 18 ha evaluated theoretically for temperate broad‐leafed forests (Bugmann et al. 1996). Manipulations were carried out in the winter of 2015/16 (Bavarian Forest, Passau), 2016/17 (Saarland, Lübeck, Hunsrück) and 2018/19 (University Forest, i.e., the forest of the University of Würzburg) to allow sufficient time for the colonisation by forest fauna. LiDAR measurements validate that the ESBC districts remained structurally more heterogeneous than the control districts during the study period (Pierick et al. 2025, *preprint*) (Figure 2).

### 2.2 Beetle Data

In each patch we set up two flight interception traps and two pitfall traps to reach sufficient sample size (number of individuals) to represent the local environmental conditions (see Müller and Brandl 2009). Sampling was conducted from May to July 2022 in the University Forest (3 sites), the Bavarian Forest (4 sites), and the Passau region (1 site), while the remaining sites were sampled in 2023. These months represent > 90% of individuals in beetle sampling during the vegetation period. Traps were emptied monthly and sorted on order level. Species identification was conducted by professional taxonomists and trait information was taken from the literature (Freude et al. 1964–1983; Koch 1989–1992; Seibold et al. 2015; Hagge et al. 2021). Details of trait selection and trait characteristics are compiled in Supplement M1.

### 2.3 Environmental and Spatial Difference

To characterise the habitat dissimilarity of patches in addition to their spatial distance, we calculated the environmental distance between any two patches of a district as their difference in quantitative attributes of forest structure with focus on canopy cover and deadwood. We calculated canopy densities measured by LiDAR via drone flights (DJI M300 L1) as the proportion of LiDAR points classified as canopy (> 7 m height) and the volume of deadwood per patch to quantify the level of ESBC. Variation in both variables was predominantly driven by our experimental ESBC manipulation. However, as natural tree mortality is still increasing in Europe (Senf et al. 2018), both also capture add‐on natural dieback processes. There might be additional, more subtle variation or changes between forest patches in environmental conditions, e.g., soil moisture, not captured by LiDAR and deadwood volume. Therefore, we additionally calculated unweighted mean Ellenberg‐indicator values based on plant relevés estimated according to a simplified percentage scale (Dittrich et al. 2013) and recorded in all patches (Ellenberg et al. 1991; Ewald 2003), which has been proven to be highly integrative in describing such subtle environmental conditions (Ewald 2003; Müller et al. 2009). We used the difference between patches of these values as a proxy for abiotic distance. To avoid overlap between information in structure measured by LiDAR and these indicator values we removed the light indicator value. Spatial distance between patches was calculated in meters based on projected coordinates in the ETRS1989 format.

### 2.4 Statistical Methods

#### 2.4.1 Meta‐Analyses

**FIGURE 3:**
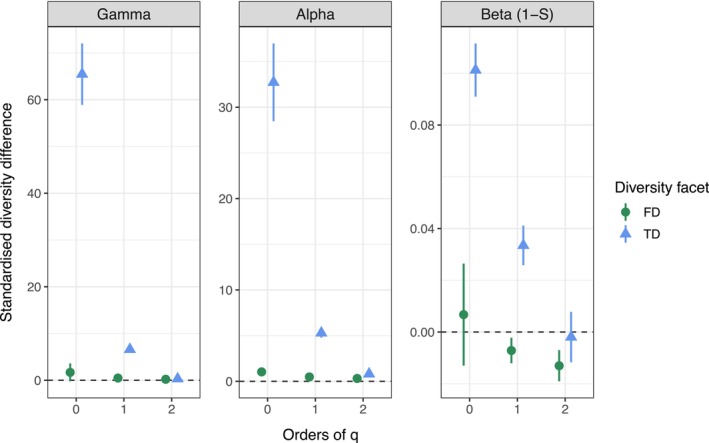
Results of the meta‐analyses based on 11 pairwise comparisons of homogeneous and heterogenized forests on γ‐, α‐, and β‐level with focus on rare (*q* = 0), common (*q* = 1), and dominant species (*q* = 2) as well as on taxonomic (TD) and functional diversity (FD) of 1308 beetle species in 234 patches. Positive values indicate higher diversity levels in ESBC districts compared to control districts. Error bars denote the 95% confidence intervals calculated from weighted averages of bootstrapped district‐specific diversity estimates for each of the 18 cases.

Statistical analyses were conducted in R (version 4.3.3) (R Core Team 2023). To allow for direct comparison, taxonomic (TD) and functional diversity (FD) were assessed as the effective number of equally abundant species and distinct functional groups and for Hill numbers *q* = 0, 1, 2 focusing on rare, common, and dominant species. These facets of beetle diversity and corresponding confidence intervals were calculated for each of the 22 districts, utilising the iNEXT.3D package (Chao et al. 2021) and the included bootstrapping approach with 200 replicates. γ‐diversity (at the landscape level) was decomposed into α (patch level) and multiplicative β‐diversity using iNEXT.beta3D (version 2.0.8) (Chao et al. 2023). We applied the 1‐S transformation, a normalised dissimilarity measure equivalent to Jaccard‐type turnover, mapping β‐diversity to the range of 0 indicating identical species composition among patches to 1 representing completely disjunct communities. Standardisation also controls for variation in patch numbers of sites (9 and 15) (Chao et al. 2019). For comparison between ESBC and control districts, we calculated differences in diversity metrics (TD and FD in combination with α‐, β‐, and γ‐diversity, as well as with *q* = 0, 1, and 2) for each of the 11 district pairs with positive values indicating higher diversity in the ESBC district. Differences from the 11 forest pairs were weighted and combined by meta‐analysis (R‐package in preparation: https://github.com/AnneChao/iNEXT.meta and code presented in Masso et al. (2025)) propagating district‐wise bootstrapping confidence intervals to the aggregated metric and being presented as error bars in Figure 3. Confidence intervals that exclude the value 0 indicate significant differences between ESBC and control districts. To control for variation in sampling effort eventually correlating with the treatment (for detailed discussion see Supplement D1), we standardised diversity estimates to a common coverage level of 0.95 of the maximum of the observed distribution (Chao et al. 2020). District‐specific results are shown in the Figures S1–S18. As a check of robustness, we compared meta‐analysis results with simple paired *t*‐tests ignoring bootstrapping confidence intervals for *q* = 0 and found very good agreement (Table S3).

#### 2.4.2 β‐Deviation

To compute pairwise β‐diversity indices (distance matrices) as a measure of dissimilarity for all study plots, we utilised the function iNEXTbeta3D_pair3D adapted from the iNEXT.beta3D package. This step was repeated for a randomised data set generated from the observed data as a reference null model. For each site, all observed individuals were shuffled between the patches of the site and thus randomly redistributed within the ESBC and control district of a site (Kraft et al. 2015; Mori et al. 2015), preserving γ‐diversity and the relative abundance of each species in a site. β‐deviation was calculated as the difference of β‐diversities between observed and randomised data. Increasing positive β‐deviation indicates a greater difference between the higher observed dissimilarity and the lower expected dissimilarity of the null model, suggesting that patches differ more in their species composition than expected under random community assembly.

#### 2.4.3 Linear Mixed Effect Models

To model the β‐deviation for the facets of diversity (taxonomic and functional diversity in combination with Hill numbers *q* = 0, 1, and 2) we used the pairwise differences in forest structure, abiotic conditions, and spatial distance as fixed factors. We estimated the contribution of each predictor treatment specific (ESBC and control district). Using this in a single model allows a direct comparison of the effect sizes for each treatment. For testing for differences in the slopes we also present the full interaction model in the Table S1. The final models were fitted using the function *lmer* of the ‘lme4’ package (Bates et al. 2015). Rather than assuming that pairwise distances are independent observations (as in conventional fixed‐effects models), we employed site as a random‐effect to capture both within‐district and among‐district dependence (Harrison et al. 2018; Chao et al. 2024). We reported *z*‐values as a standardised metric of effect sizes.

To additionally analyse when FD patterns reveal metacommunity assembly processes, we examined functional β‐diversity independently from the changes in TD by using another null model approach. For 100 replicates of the null model, we permuted species labels across the species trait matrix, while the species abundance matrix and thus its spatial pattern was kept unchanged (Jeliazkov and Chase 2024). We calculated the standardised effect size (SES) normalising observed functional β‐diversity by mean and standard deviation of the randomised samples. Positive SES were considered to indicate higher functional turnover relative to taxonomic turnover, whereas a SES lower than 0 indicated lower functional turnover. Absolute differences greater than 1.96 indicate significant effects. Finally, we compared SES levels in control and ESBC districts by a GLMM again with site as a random factor.

## 3 Results

### 3.1 Diversity

Our final data set comprised 1308 beetle species (37–143 per patch) from 103,714 specimens (153–1888 per patch). We could identify 890 different species in the traps when pooled over the control districts and 1142 in the experimental districts with an overlap of 724 species. 166 species in control districts were not found in ESBC districts. Note that these observed quantities had to be standardised by sample coverage for the statistical analysis. Standardised species richness at the γ‐level ranged from 70 to 275 species in the different control districts (Figure S4) and from 138 to 425 species in ESBC districts (for a full species list in control and ESBC districts ordered by taxonomy and their abundances and frequencies see Table S2 and Figures S1–S18 for all facets of diversities per district). The meta‐analyses showed the most pronounced effect size in effective species number for γ‐diversity in ESBC districts by an increase of about 60 species compared to control districts when species were weighted equally and independent of their abundancies (*q* = 0, Figure 3, see Figure S19 for the observed distribution of species with low abundance). This was driven by both an increase in α‐diversity of about 30 species and of β‐diversity of about 10%. When increasing the contribution of more abundant species to the diversity estimate (*q* = 1) the effective number of species also increased at the landscape scale, but by six species only, again driven by α‐ and β‐diversity. For dominant species (*q* = 2), we found no effect on γ‐diversity, but there was a small increase in α‐diversity (0.8 species). Except for *q* = 2, β‐diversity of FD (effective numbers of functional groups) showed lower responses to ESBC forests than TD, which is an indication of a lack of turnover of functional traits (or can be referred to as redundancy) in the traits that were measured. Here the effects were most pronounced for β‐diversity. From rare to dominant species, the increase in functional β‐diversity differences between ESBC and control districts turned into a significantly negative effect.

### 3.2 β‐Deviation

Modelling of the β‐deviation for all Hill numbers and facets of diversity showed strong positive effects of the manipulated differences between patches via forest structure in ESBC districts, for all Hill numbers of TD as well as higher levels of explained variance than for FD (Table 1). Spatial distance was significant here only for β‐deviation with focus on common species (*q* = 1). The *z*‐values of structure versus space were much larger in the ESBC districts, indicating the dominance of a *species‐sorting* effect in the ESBC patches. In the control districts, β‐deviation with focus on rare and common species was also affected by structural differences which were present to a small extent even without the heterogenizing treatment. However, in common and dominant species, space was the most relevant predictor. Differences in abiotic conditions, using Ellenberg indicator values as a proxy for unmeasured spatial abiotic heterogeneity, did not significantly contribute to explaining taxonomic β‐deviation, suggesting that most of the environmental heterogeneity was already captured by the experimentally manipulated and measured variables. Conditional R squared values were highest for TD (Table 1).

**TABLE 1:** Results of linear mixed interaction models of the β‐deviation, i.e., the difference between β‐diversity of observed vs. null‐model data (with randomised distribution of individuals across patches in each site) for taxonomic (TD) and functional (FD) β‐diversity in combination with Hill numbers *q* = 0, 1, 2 (rare, common, and dominant species). As predictors we used the structural difference between patches (Structure), the difference in space (Spatial), and the difference in abiotic conditions (Abiotic) extracted from Ellenberg indicator values for both levels of deadwood and canopy structure (Control vs. ESBC), as well as Site as random factor. Last row provides the conditional *R* squared value of the model. Bold values indicate significant estimates.

Diversity facet	TD	TD	TD	FD	FD	FD
Hill number *q*	**0**	**1**	**2**	**0**	**1**	**2**
Control:Structure	**3.628**	**3.196**	1.579	−0.068	0.352	0.340
ESBC:Structure	**4.936**	**11.75**	**7.685**	**3.865**	0.509	0.495
Control:Abiotic	−0.256	−1.155	−0.467	1.258	**−2.196**	−1.673
ESBC:Abiotic	1.124	1.152	1.807	**−2.007**	0.530	0.790
Control:Spatial	0.835	**6.994**	**7.229**	0.878	**5.844**	**6.287**
ESBC:Spatial	1.538	**3.092**	1.715	0.017	1.230	1.340
*R* ^2^ conditional	0.11	0.35	0.38	0.071	0.15	0.18

The β‐deviation of FD revealed similar patterns. In the treatment districts, the structural heterogeneity increased FD of rare species, additionally supporting the *species‐sorting* effect due to differences in species' attributes. In homogeneous control districts, however, functional diversity with a focus on common and dominant species increased with increasing spatial distance, which supports the *patch‐dynamics* archetype with species showing differences in dispersal and competition.

Standardised effect sizes of mean pairwise distances in functional β‐diversity significantly decreased from control districts to ESBC districts for common and rare species (Table S1 and Figure S20) indicating additionally a transition towards metacommunities more affected by habitat filtering even when controlling for TD.

## 4 Discussion

Our results supported our first hypothesis that heterogenization of a homogeneous temperate production forest increases the diversity of beetles but only with focus on less abundant species, while dominant species remained unaffected or were even disfavoured. The finding that the increase in γ‐diversity was determined by an increase in α‐ and β‐diversity confirmed the predictions that both *mass‐effects* and *species‐sorting* contribute to the higher γ‐diversity. This was also supported by the increase in FD again on the α‐scale. Furthermore, the consistent result of larger effects of spatial distance between forest patches on β‐deviation in homogeneous forests shifting to reduced spatial effects, but an increase of forest structure effects in heterogeneous forests, supported that ESBC forests are more driven by *species‐sorting* processes rather than *patch‐dynamics*. This could particularly be observed for FD which reflects species differences in habitat requirements and mobility and thus is linked to *species‐sorting*.

### 4.1 Patch‐Dynamics

The results in homogeneous forests are mostly in accordance with metacommunities dominated by *patch dynamics* (Levins and Culver 1971; Leibold et al. 2004). By centuries of human management, managed forests are relatively homogeneous in tree dimensions and vertical/horizontal structure, so compositional differences among patches are expected to be driven primarily by spatial processes rather than by strong environmental contrasts. Accordingly, we expect that dispersal limitation and stochastic demographic processes would contribute substantially to β‐diversity patterns, producing a detectable effect of geographic distance even after accounting for measured environmental differences (Holyoak et al. 2005). Such distance–decay can be consistent with *patch‐dynamics*‐type colonisation–extinction dynamics and also ecological drift, and therefore we interpret it as evidence for the importance of spatially structured processes (Chase and Myers 2011) in homogeneous forests. Beetles show a wide range in the dispersal ability from flightless species to species dispersing actively over many kilometres (Buse 2012; Komonen and Müller 2018). Even though the maximum distance between two patches within a district was only ~1 km, which might be bridged by all species in our data set (Komonen and Müller 2018), we found a clear spatial effect independent from environmental differences in both homogeneous and heterogeneous forests. This was supported even for the direct measures of functional β‐deviation. *Patch‐dynamics* occurring in both forest types but being more pronounced in homogeneous forests suggests that this mechanism always contributes to metacommunities of beetles in forests but dominates in homogeneous ones.

The beetles in our data do not only differ in their abundance and mobility (from flightless to highly mobile), but also in their ability to outcompete other species after colonisation. For instance, a prominent example is Ambrosia beetles. They have evolved highly flexible life history strategies during the colonisation of new patches, e.g., by sib‐mating without inbreeding depression, changing the level of social behaviour, and adopting a complex mutualism with their ambrosia fungi (Biedermann 2010; Biedermann et al. 2011). In contrast, other beetle species are less flexible in traits resulting in a gradient of species' location on the trade‐off association between dispersal ability and competitive strength as required for *patch‐dynamics*.

### 4.2 Species‐Sorting

MacArthur and MacArthur (1961) laid the cornerstone for the habitat heterogeneity hypothesis using the gradient of vertical heterogeneity of forests as an axis of different niches for bird species. Later, this single gradient has been expanded with horizontal heterogeneity as one of the overall most prominent ones among taxa and functions (Heidrich et al. 2020). In this light, our substantial manipulation of forest structure, including the horizontal heterogeneity and amount of deadwood should lead to *species‐sorting* (Leibold et al. 2004; van der Gucht et al. 2007; Hernandez‐Ordonez et al. 2019). We expected this especially, because we manipulated deadwood, which promotes many obligatory, but also facultative saproxylic arthropod and particularly beetle species (Seibold et al. 2016; Graf et al. 2022). In fact, the increase in β‐diversity and β‐deviation with increasing differences in forest structure, supports this at least for rare and common species. The question remains, why we could not find an effect on diversity of dominant species. Here we must note that our local interventions did not change the structure of deadwood and light availability of large, forested landscapes. So, the majority of the forest remained even‐aged with closed‐canopy, making specifically the dominant species in this ecosystem resistant to the small scale ESBC treatments. However, dominant species can be affected by large scale habitat heterogeneity. An analysis of the rarity level of the same forest beetles in Europe in response to wilderness areas revealed that rare species in Europe were dominant in the wilderness areas of Mongolia and vice versa (Müller et al. 2013). Similarly, we have reports of single species, e.g., the highly endangered *Peltis grossa* (Trogossitidae) formerly very rare due to homogenization in modern forestry over centuries, turning into a dominant species after landscape‐wide pulses of deadwood by windstorms and bark beetle disturbances on a regional with intensities many times higher than in our experiment (Busse et al. 2022). Together with our findings, this shows that our patch‐wide interventions were insufficient to affect the *species‐sorting* of dominant species in production forests of the temperate European zone.

### 4.3 Mass‐Effect

The second archetype commonly stressed in metacommunity theory with increasing heterogeneity is the *mass‐effect* stimulating source‐sink dynamics (Mouquet and Loreau 2003; Leibold et al. 2004). It has been stated that without information of dispersal rates or frequencies it cannot be distinguished from the *species‐sorting* effect (Logue et al. 2011). However, our comprehensive approach on different spatial scales provides some support that the *mass‐effect* is a critical mechanism providing higher γ‐diversity in ESBC forests, as we found a clear effect of the treatment on α‐diversity. This indicates that the gain of the resources (deadwood and sunlight) in the formerly homogeneous, dark forest locally increases the diversity of beetles. Considering that spatial distance additionally affected the β‐deviation beyond the pronounced effects of forest structure suggests that at least the increase in common species is also triggered by *mass‐effects*. In addition, it could be responsible for the slightly negative effect in β‐diversity in dominant species (Figure 3), when massive dispersal of the most frequent species is promoted eventually resulting in homogenization of species distribution (Mouquet and Loreau 2002; Pardini et al. 2010). We could not identify such a spatial effect on β‐deviation for rare species, but we found a strong effect on α‐diversity of rare species. This might be explained by the fact that many rare species are promoted by resource pulses at the α‐scale, but as they are too rare, this does not display in β‐deviations. Nevertheless, the large contribution of α‐diversity to higher γ‐diversity in ESBC districts cannot be explained only by β‐diversity suggesting an overall *mass‐effect*.

### 4.4 Facets of Diversity

A statistical response in FD constituted by treatment‐related traits as in our study indicates a genuine response of functional composition to the treatment. Jeliazkov and Chase's (2024) synthesis of many biodiversity studies showed that TD likely masks a functional effect by placeholder species, a potentially important reason why studies in general find weaker effects in FD than in TD (e.g., Carvalho et al. 2020; Saito et al. 2020; Peng et al. 2021; Tison‐Rosebery et al. 2022) and a potential explanation of the reduced amount of explained variance in functional β‐deviation compared to taxonomic β‐deviation in our study. A similar argument was applied to advocate for FD over TD as it allowed to remove the effects of regional species pools (Püls et al. 2025). This is in line with Cadotte and Tucker's (2017) claim that “inferences about the importance of the environment cannot rely on compositional data alone, and that species abundances, population growth, or traits must be correlated with the environment” to disentangle environmental filtering from interspecific interactions. Inspecting the significance patterns in FD in Table 1, we can refine the coarser results from TD: Effects of structure in ESBC districts (indicating species sorting) are significant only for rare species, while space dominates β‐deviation in control plots which is in good agreement with the expectation of more successful dispersal with higher Hill exponents eventually constituting a mass effect.

In contrast to Jeliazkov and Chase (2024) we based our standardised effect sizes (SES) calculation on the multiplicative Hill number approach for diversity decomposition (Chao et al. 2023), which allows us to differentiate the perspective between rare, common, and dominant species. For common and dominant species, we found mostly positive SES values indicating trait divergence, which can reflect limiting similarity due to selection and competitive exclusion (Jeliazkov and Chase 2024).

### 4.5 From Mechanisms to Management Strategies

At the midpoint of the UN decade of restoration, success is still limited. From a forest management perspective, increasingly frequent natural disturbances are used as guidelines for new strategies aiming to improve resistance and biodiversity of forests (Aszalós et al. 2022). Many of these strategies aim to locally increase the number of old‐growth attributes, such as trees with microhabitats or deadwood (Bauhus et al. 2009) or to mimic natural disturbance events (Seibold et al. 2016). However, natural events provide no standardised design, are most often not replicated, and spatially not independent. These factors prevent the necessary comprehensive understanding of the effects on diversity and the essential mechanisms of metacommunity theory. In addition, they increase the risk of misinterpretation and might even contribute to establish unproven paradigms (Weisser et al. 2023). In contrast to long lasting expectations, two large global assessments recently found that land use intensity does not consistently form homogeneous communities, and that higher γ‐diversity is often driven by increasing α‐diversity, while effects of β‐diversity were less consistent (Gonçalves‐Souza and Sanders 2025; Keck et al. 2025). Thus, the combination of both strategies, an opportunistic increase of resources generated by dieback of trees, summer storms, or droughts as well as a targeted spatial heterogeneity in forests, seem very promising in understanding and restoring the beetle diversity of homogeneous production forests, which could be extended to other taxa.

## Author Contributions

J.M., M.W.C., A.S.M., F.P., S.T., M.W.C., O.M. and W.W. designed the study, J.R., C.B., M.B., P.H.W.B., P.B., A.C., O.D., B.D., S.D., A.F., A.K., L.K.‐A., G.O., S.T., L.V., L.L., L.P., J.S.‐W., H.F., and C.W. performed the research, A.C. provided the meta‐analysis, and J.M. and O.M. wrote the first draft of the manuscript, and all authors contributed substantially to revisions.

## Funding

This work was supported by the Deutsche Forschungsgemeinschaft [BETA‐FOR, 459717468]. J.R. received funding from the Bavarian State Ministry for Food, Agriculture, Forestry and Tourism (grant no. L062).

## Supporting information


**Data S1:** ele70355‐sup‐0001‐supinfo.pdf.

## Data Availability

Data and code are available at https://figshare.com/s/4b6b4d5a5cfbcf0dbac3. https://doi.org/10.6084/m9.figshare.29994688 is reserved to be published when the manuscript is accepted.
