# Supplementary material for: Forest Heterogeneity by Chain Saw: How Between‐Patch Variation in Old Growth Attributes Changes the Metacommunities of Beetles
Source: Ecol Lett. 2026 Mar 5;29(3):e70355. doi: 10.1111/ele.70355 (PMC12962797; doi:10.1111/ele.70355)
Supplement: Supplementary file 1 — Data S1: ele70355‐sup‐0001‐supinfo.pdf. [file ELE-29-0-s001.pdf]

Supplement of **“Forest heterogeneity by chain saw: how between-patch variation in old growth attributes changes the metacommunities of beetles”**

Introduction I1: Interactions of beetles, anvorinment, and other species

Beetles are involved in many different interspecific interactions with plants, fungi, other insects, and vertebrates, eventually relativizing the direct impact of the environment. Many weevils (Curculionidae), leaf beetles (Chrysomelidae) and long-horn beetles (Cerambycidae) consume foliage, needles, seeds, or wood from various tree species (Vogel et al. 2021); many are host or habitat specialists (Brändle & Brandl 2001). Bark beetles (Scolytinae) excavate galleries beneath the bark, thereby weakening host trees and some of them are able to trigger mass outbreaks (Bussler et al. 2011). Mutualistic ambrosia beetles (Scolytinae) cultivate fungal gardens within wood as a primary food source, with fungi gaining from vector-mediated transport and dispersal (Kirkendall et al. 2015). Xylophagous beetles facilitate mycorrhizal fungal dissemination or decompose dead wood in symbiosis with bacteria and fungi (Birkemoe et al. 2018, Seibold et al. 2019). Predatory ground beetles (Carabidae) and rove beetles (Staphylinidae) prey on gastropods, annelids, and insect larvae; approximately 30–35% of wood-dwelling beetles are predatory. Detritivorous burying beetles (Silphidae) bury vertebrate carcasses, competing with avian and mammalian scavengers for carrion, while larder beetles degrade insect remains (Seibold et al. 2019). Saproxylic beetles can accelerate dead wood decomposition, enhancing nutrient cycling (Zou et al. 2023). While some beetles as the checkered beetles (Cleridae) are specialized predators on bark beetles, many other beetle species can also promote the habitat of other beetle and insect species (Weslien et al. 2012).

Methods M1: Trait selection

As traits we selected

1) body size, taken as mean values of minimum and maximum from the literature (Freude et al. 1964-1983; Hagge et al. 2021), ranging from 0.5 to 50 mm, as a universal characteristic for aspects of reproduction, dispersal ability, and general population size (Chown & Gaston 2010), but also related to deadwood amount (Gossner et al. 2013),

2) brightness of beetle species, estimated by Hagge et al. (2021) using the workflow of a computer-assisted digital image analysis system. Digital photos (Dries 2016, unpublished, print on demand, in Hagge et al. 2021) served as the reference. Beetle species were photographed under standardized conditions in dorsal view against a white background. Subsequently, the white background of the red, green and blue (RGB) photos was removed and the mean of the RGB color channel across all pixels of each image was calculated. Brightness values of our beetles ranged from 69 (dark) to 155 (bright species). Brightness indicates the species' response to climate conditions (Zeuss et al. 2014) and specifically canopy openness, here manipulated by gap creation in spatially distributed and aggregated patterns

3) the adoption of a life style in deadwood (saproxylic) as a binary factor which could support saproxylic species in patches with deadwood accumulation (Graf et al. 2022), taken from Hagge et al. (2021), and

4) the feeding strategy, taken from Koch (1989-1992), discriminating between seven factor levels: coprophagous, mycetophagous, necrophagous, phytophagous, saprophagous, xylophagous, and zoophagous with variable advantages in response to the treatment combinations and which also can be linked to canopy openness (Heidrich 2020).

Discussion D1: Sample coverage standardization in the framework of Hill numbers

Our study implements the concluding suggestion of Jeliazkov and Chase (2024) to consider scale-explicit approaches depending on disturbance, scales, and decomposition into α-, γ-, and specifically β-components for improving metacommunity analysis. However, a particular problem in β-diversity analyses has been observed for many years. In observational data, a fraction of β-response can be regularly attributed to locally varying unobserved species resulting in artifacts of the analysis which distort the results (Tuomisto 2010). This issue is even more pronounced in experimental approaches with substantial, independent variation in predictors where sample coverage has been shown to systematically correlate with treatment (Kortmann et al. 2025b; Rothacher et al. 2025) which is increasingly relevant in highly diverse taxa with many unobserved species, as in beetles. It does not only matter in species-rich and structurally extremely heterogeneous tropical forests, but also in European temperate zones. Recent analyses showed that many studies with insect samples from temperate zones are incomplete, in cases using flight and pitfall traps in beetles, light trapping of moths or even for total insect data from malaise traps (Müller & Brandl 2009; Kortmann et al. 2025a; Püls et al. 2025). In addition, it especially affects the conclusions from null model approaches which are evaluating the relative role of different assembly processes in a spatially hierarchical setting (Mori et al. 2015a). Tucker et al. (2016) demonstrated this effect of a higher β-diversity in undersampled communities with simulated data and suggested developing methods to control for it. This has been solved recently by standardizing not only diversity by the comprehensive approach of attribute diversity, but also dissimilarity between communities, by controlling for sample coverage (Chao et al. 2023).

In contrast to the widely used Bray-Curtis-dissimilarity the generalized concept is applicable even to dissimilarities along Hill numbers and enables a much wider range of β-diversity applications. Moreover, this unified approach for TD, FD, and even PD (not included here), does not only allow direct comparisons of the different units, but also to generalize the β-diversity concept to the different aspects of species dissimilarity as previously proposed by Logue et al. (2011). Finally, the difference is not only reflected in traits. Besides species identity, species abundance substantially affects population dynamics and community assembly. Populations of more abundant species have, for instance, higher dispersal potential than smaller populations and are less affected in survival by negative stochastic effects even if sometimes most affected in general (see for insects in Müller et al. 2024; van Klink et al. 2024). Hence, the use of Hill numbers in our study provides a generalized framework to investigate metacommunity patterns from different angles just by using the abundance distributions of species (see also Guzman et al. 2022).

Figures S1 to S18: All, i.e. in total 18, meta-analyses of taxonomic (TD) and functional (FD) diversity (3x) at coverage level of 0.95 for Hill numbers q=0, 1, and 2 (3x) at alpha, gamma, and beta (1-S) scale (3x), each for eleven study sites. The bottom row of each figure indicates the aggregated results at the meta-analysis level with bootstrapping confidence levels (lower LCL, upper UCL) indicating overall significance when not including 0. W(fixed) in each row indicates weighting of each site within the meta-analysis according to site-specific confidence intervals. Column Difference gives the difference between estimated diversity for Enhanced ESBC and Control districts. These analyses are summarized in Fig. 3 of the main manuscript.

Alpha with Coverage = 0.95

Site	TD(q=0) Enhanced	TD(q=0) Control	Difference	LCL	UCL	W(fixed)
B04	127.721	80.161	47.561	33.47	61.652	9.1%
B05	134.019	128.59	5.429	−10.189	21.047	7.41%
B06	98.816	89.523	9.293	−4.773	23.358	9.14%
B07	108.028	55.51	52.518	42.434	62.602	17.77%
H09	109.694	76.586	33.109	16.014	50.203	6.18%
L11	127.894	80.684	47.21	29.393	65.027	5.69%
P08	150.04	129.981	20.059	3.615	36.503	6.68%
S10	100.687	81.678	19.01	7.239	30.78	13.05%
U01	173.954	133.012	40.942	27.209	54.675	9.58%
U02	178.462	134.919	43.543	28.511	58.575	8%
U03	182.073	157.026	25.047	9.413	40.682	7.39%
**Meta analysis**			**32.728**	**28.477**	**36.979**	

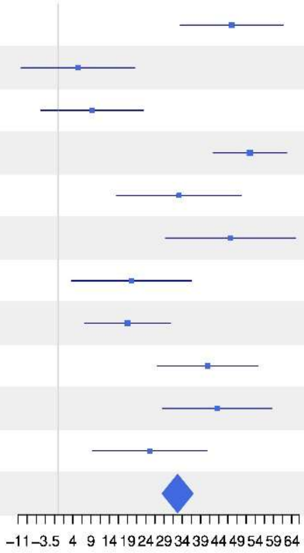

Alpha with Coverage = 0.95

Site	TD(q=1) Enhanced	TD(q=1) Control	Difference	LCL	UCL	W(fixed)
B04	20.742	16.296	4.445	2.868	6.023	10.36%
B05	27.24	40.528	−13.288	−16.063	−10.514	3.35%
B06	15.448	13.383	2.064	0.87	3.258	18.09%
B07	12.701	7.04	5.661	4.865	6.456	40.74%
H09	32.301	22.158	10.143	7.758	12.528	4.53%
L11	45.822	14.91	30.912	27.259	34.566	1.93%
P08	23.64	32.536	−8.896	−11.047	−6.745	5.57%
S10	23.879	24.692	−0.814	−2.736	1.108	6.98%
U01	57.149	27.013	30.136	27.704	32.568	4.36%
U02	62.756	32.132	30.624	27.324	33.924	2.37%
U03	57.39	54.471	2.919	−0.971	6.809	1.7%
**Meta analysis**			**5.289**	**4.781**	**5.797**	

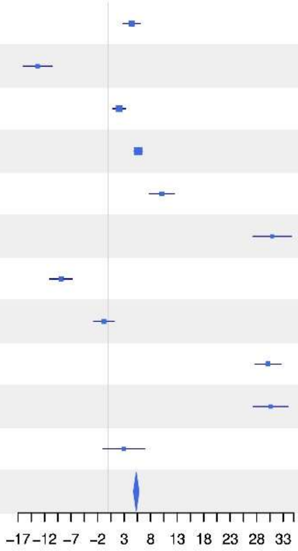

Alpha with Coverage = 0.95

Site	TD(q=2) Enhanced	TD(q=2) Control	Difference	LCL	UCL	W(fixed)
B04	6.482	6.849	−0.367	−0.948	0.213	7.71%
B05	8.27	17.295	−9.024	−10.311	−7.738	1.57%
B06	5.961	4.617	1.344	0.97	1.719	18.55%
B07	3.736	2.65	1.086	0.877	1.294	59.74%
H09	17.024	10.137	6.888	5.667	8.108	1.75%
L11	18.55	5.372	13.178	11.425	14.93	0.85%
P08	2.624	13.224	−10.6	−11.405	−9.795	4.01%
S10	8.81	10.941	−2.131	−3.092	−1.17	2.81%
U01	20.781	6.026	14.754	13.441	16.067	1.51%
U02	22.543	7.346	15.197	13.398	16.996	0.8%
U03	18.735	20.741	−2.006	−3.937	−0.075	0.7%
**Meta analysis**			**0.805**	**0.644**	**0.967**	

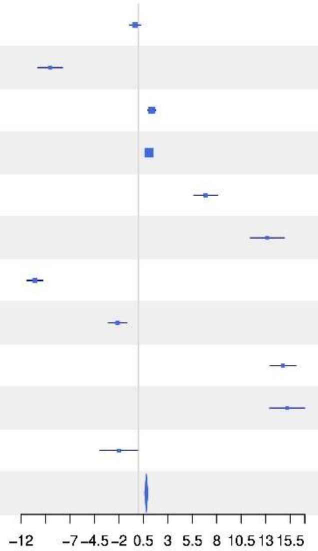

Gamma with Coverage = 0.95

Site	TD(q=0) Enhanced	TD(q=0) Control	Difference	LCL	UCL	W(fixed)
B04	177.424	102.738	74.686	54.8	94.572	10.88%
B05	210.24	198.799	11.442	−12.377	35.261	7.58%
B06	137.771	106.836	30.934	14.733	47.136	16.39%
B07	148.443	70.203	78.24	64.712	91.769	23.51%
H09	169.124	112.93	56.194	32.583	79.805	7.72%
L11	267.236	107.256	159.98	124.816	195.143	3.48%
P08	215.451	168.128	47.323	25.712	68.934	9.21%
S10	146.474	125.804	20.67	−0.504	41.844	9.6%
U01	360.988	256.097	104.891	73.085	136.697	4.25%
U02	425.328	242.372	182.956	149.286	216.625	3.8%
U03	425.248	275.908	149.34	114.717	183.962	3.59%
**Meta analysis**			**65.464**	**58.905**	**72.023**	

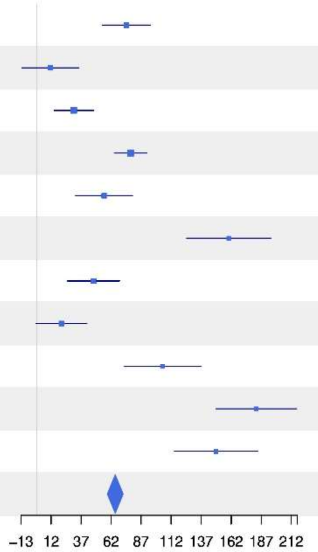

Gamma with Coverage = 0.95

Site	TD(q=1) Enhanced	TD(q=1) Control	Difference	LCL	UCL	W(fixed)
B04	25.384	19.804	5.58	3.231	7.929	8.41%
B05	34.144	53.106	−18.962	−22.533	−15.391	3.64%
B06	21.054	14.962	6.092	4.669	7.515	22.93%
B07	15.537	10.686	4.851	3.831	5.871	44.63%
H09	39.811	28.555	11.256	8.368	14.144	5.57%
L11	69.047	17.462	51.584	46.08	57.089	1.53%
P08	45.067	42.413	2.654	−0.714	6.021	4.09%
S10	29.865	32.599	−2.734	−5.726	0.258	5.19%
U01	99.511	52.324	47.186	41.99	52.383	1.72%
U02	119.887	56.095	63.793	57.585	70	1.2%
U03	110.448	83.254	27.194	20.637	33.751	1.08%
**Meta analysis**			**6.599**	**5.917**	**7.28**	

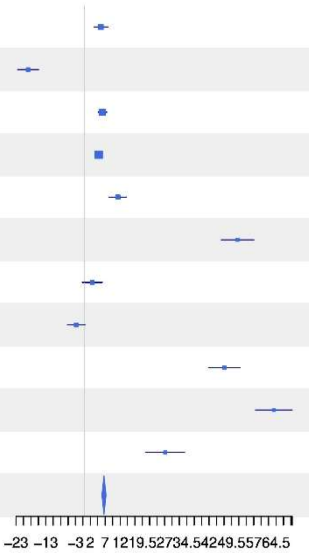

Gamma with Coverage = 0.95

Site	TD(q=2) Enhanced	TD(q=2) Control	Difference	LCL	UCL	W(fixed)
B04	7.145	8.096	−0.952	−1.607	−0.296	9.83%
B05	9.76	26.596	−16.836	−18.501	−15.171	1.52%
B06	8.187	4.921	3.266	2.743	3.79	15.41%
B07	4.304	4.504	−0.201	−0.456	0.055	64.58%
H09	21.861	13.657	8.204	6.727	9.681	1.94%
L11	26.976	6.225	20.751	17.981	23.521	0.55%
P08	13.771	17.88	−4.109	−5.395	−2.824	2.55%
S10	10.957	14.647	−3.69	−5.002	−2.378	2.45%
U01	39.779	18.85	20.929	18.017	23.841	0.5%
U02	48.974	22.395	26.579	23.065	30.094	0.34%
U03	43.154	39.179	3.975	0.335	7.616	0.32%
**Meta analysis**			**0.309**	**0.104**	**0.515**	

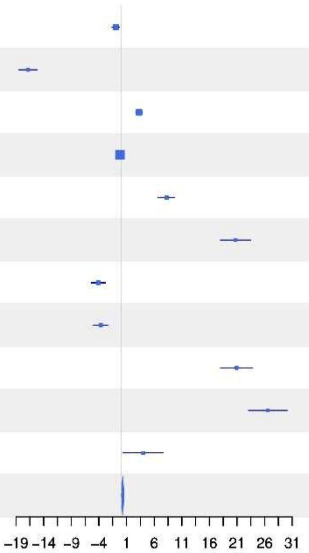

1–S with Coverage = 0.95

Site	TD(q=0) Enhanced	TD(q=0) Control	Difference	LCL	UCL	W(fixed)
B04	0.315	0.247	0.068	0.025	0.111	5.68%
B05	0.408	0.397	0.011	–0.025	0.046	8.54%
B06	0.318	0.182	0.136	0.086	0.185	4.3%
B07	0.306	0.235	0.071	0.016	0.126	3.45%
H09	0.395	0.362	0.033	–0.018	0.085	4%
L11	0.587	0.279	0.308	0.266	0.349	6.14%
P08	0.342	0.255	0.086	0.048	0.124	7.35%
S10	0.352	0.395	–0.043	–0.094	0.008	4.08%
U01	0.555	0.515	0.04	0.017	0.064	19.04%
U02	0.622	0.475	0.147	0.123	0.171	18.81%
U03	0.613	0.462	0.151	0.127	0.175	18.61%
**Meta analysis**			**0.101**	**0.091**	**0.112**	

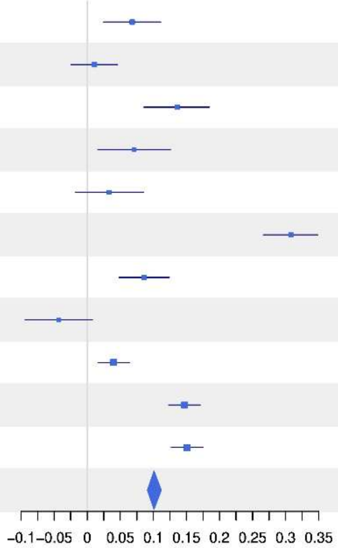

1–S with Coverage = 0.95

Site	TD(q=1) Enhanced	TD(q=1) Control	Difference	LCL	UCL	W(fixed)
B04	0.206	0.199	0.006	–0.026	0.039	5.4%
B05	0.227	0.266	–0.039	–0.069	–0.009	6.5%
B06	0.3	0.119	0.181	0.152	0.21	6.83%
B07	0.205	0.384	–0.178	–0.201	–0.156	11.1%
H09	0.212	0.252	–0.04	–0.07	–0.009	6.27%
L11	0.378	0.164	0.214	0.176	0.252	4.01%
P08	0.535	0.262	0.273	0.247	0.299	8.81%
S10	0.225	0.273	–0.047	–0.08	–0.015	5.45%
U01	0.456	0.518	–0.062	–0.081	–0.044	17.28%
U02	0.511	0.458	0.053	0.034	0.072	15.94%
U03	0.515	0.37	0.144	0.123	0.166	12.42%
**Meta analysis**			**0.033**	**0.026**	**0.041**	

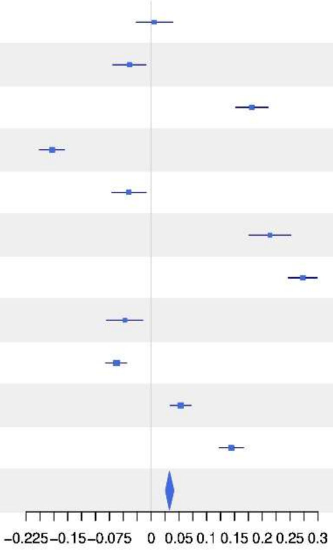

1−S with Coverage = 0.95

Site	TD(q=2) Enhanced	TD(q=2) Control	Difference	LCL	UCL	W(fixed)
B04	0.104	0.173	−0.069	−0.095	−0.043	13.82%
B05	0.172	0.393	−0.222	−0.264	−0.179	5.25%
B06	0.306	0.07	0.236	0.208	0.265	11.45%
B07	0.148	0.463	−0.315	−0.344	−0.286	11.25%
H09	0.249	0.29	−0.041	−0.084	0.002	5.06%
L11	0.351	0.154	0.197	0.139	0.255	2.81%
P08	0.911	0.293	0.618	0.588	0.648	10.67%
S10	0.22	0.285	−0.064	−0.106	−0.022	5.36%
U01	0.512	0.729	−0.217	−0.244	−0.19	13.1%
U02	0.578	0.72	−0.142	−0.169	−0.115	12.95%
U03	0.606	0.504	0.102	0.068	0.136	8.28%
**Meta analysis**			**−0.002**	**−0.012**	**0.008**	

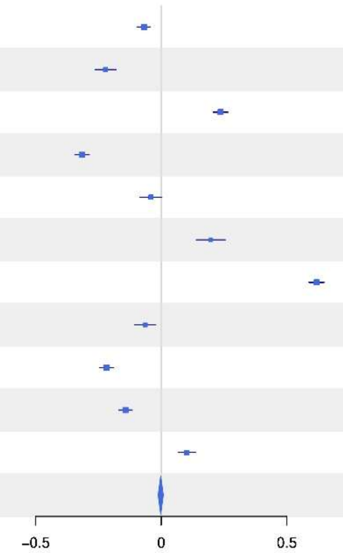

Alpha with Coverage = 0.95

Site	FD(q=0) Enhanced	FD(q=0) Control	Difference	LCL	UCL	W(fixed)
B04	11.079	9.525	1.554	−0.186	3.294	7.08%
B05	11.688	11.474	0.214	−1.272	1.7	9.71%
B06	9.736	9.578	0.158	−1.16	1.476	12.33%
B07	10.623	7.989	2.634	1.174	4.094	10.06%
H09	10.675	9.129	1.546	−0.082	3.174	8.09%
L11	11.556	9.972	1.584	−0.103	3.27	7.54%
P08	11.081	10.649	0.431	−1.126	1.988	8.85%
S10	9.764	9.118	0.645	−0.509	1.8	16.08%
U01	12.628	11.368	1.26	−0.278	2.798	9.07%
U02	12.856	11.708	1.149	−0.842	3.139	5.41%
U03	13.004	12.084	0.921	−1.003	2.845	5.79%
**Meta analysis**			**1.031**	**0.568**	**1.494**	

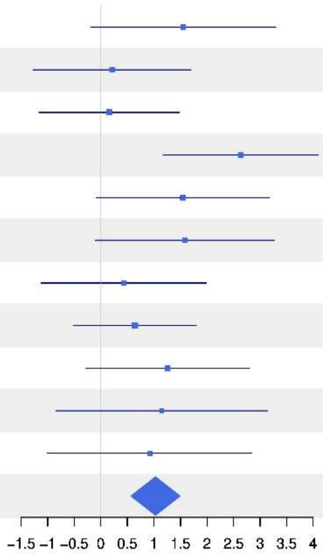

Alpha with Coverage = 0.95

Site	FD(q=1) Enhanced	FD(q=1) Control	Difference	LCL	UCL	W(fixed)
B04	4.501	4.401	0.1	−0.1	0.301	8.51%
B05	5.149	6.203	−1.054	−1.305	−0.802	5.42%
B06	4.05	3.708	0.343	0.188	0.497	14.4%
B07	3.585	2.768	0.817	0.696	0.938	23.47%
H09	5.408	4.834	0.573	0.332	0.815	5.88%
L11	6.205	4.098	2.106	1.803	2.41	3.72%
P08	4.062	4.921	−0.859	−1.022	−0.696	12.9%
S10	4.837	4.729	0.108	−0.1	0.317	7.88%
U01	6.787	4.568	2.219	2.012	2.426	8.01%
U02	7.069	5.2	1.869	1.607	2.132	4.97%
U03	6.707	6.594	0.112	−0.154	0.379	4.84%
**Meta analysis**			**0.478**	**0.42**	**0.537**	

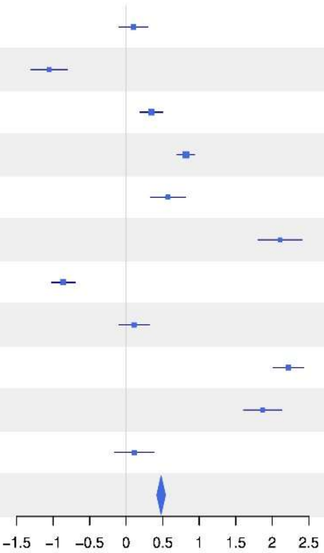

Alpha with Coverage = 0.95

Site	FD(q=2) Enhanced	FD(q=2) Control	Difference	LCL	UCL	W(fixed)
B04	3.193	3.278	−0.085	−0.207	0.037	9.82%
B05	3.584	4.589	−1.005	−1.184	−0.826	4.57%
B06	2.986	2.612	0.374	0.279	0.468	16.48%
B07	2.399	1.723	0.676	0.606	0.746	29.7%
H09	4.193	3.666	0.527	0.364	0.69	5.52%
L11	4.167	2.849	1.317	1.078	1.557	2.56%
P08	1.625	3.437	−1.812	−1.931	−1.694	10.41%
S10	3.556	3.472	0.084	−0.072	0.24	6.06%
U01	4.761	2.508	2.253	2.1	2.407	6.26%
U02	4.914	3.015	1.899	1.72	2.077	4.61%
U03	4.566	4.661	−0.095	−0.286	0.096	4.01%
**Meta analysis**			**0.312**	**0.274**	**0.351**	

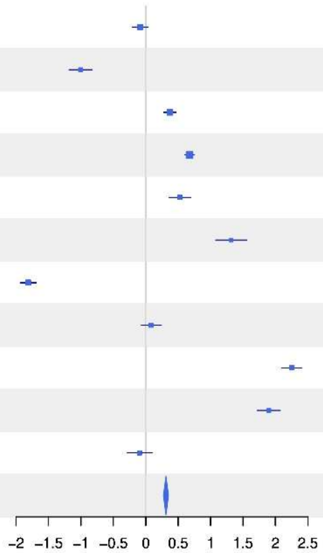

Gamma with Coverage = 0.95

Site	FD(q=0) Enhanced	FD(q=0) Control	Difference	LCL	UCL	W(fixed)
B04	11.544	9.808	1.736	−5.793	9.266	6.43%
B05	12.736	12.345	0.392	−6.729	7.512	7.19%
B06	10.323	9.688	0.635	−4.279	5.549	15.1%
B07	11.105	8.412	2.693	−1.197	6.582	24.1%
H09	11.048	9.619	1.429	−5.803	8.662	6.97%
L11	13.639	9.874	3.764	−5.014	12.543	4.73%
P08	12.116	10.849	1.267	−5.431	7.966	8.13%
S10	10.43	9.831	0.599	−4.197	5.396	15.85%
U01	15.827	13.513	2.313	−5.652	10.278	5.75%
U02	16.854	13.346	3.508	−8.158	15.174	2.68%
U03	17.03	13.634	3.396	−7.519	14.31	3.06%
**Meta analysis**			**1.691**	**−0.218**	**3.601**	

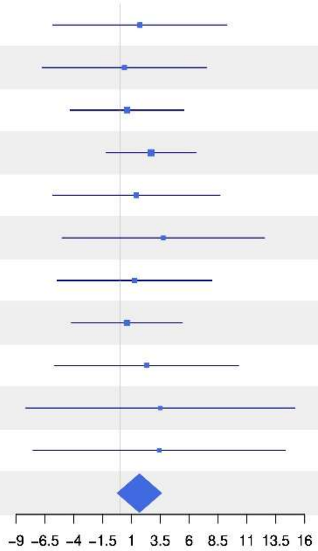

Gamma with Coverage = 0.95

Site	FD(q=1) Enhanced	FD(q=1) Control	Difference	LCL	UCL	W(fixed)
B04	4.78	4.761	0.019	−0.233	0.271	9.25%
B05	5.5	6.806	−1.306	−1.652	−0.96	4.93%
B06	4.654	3.824	0.829	0.647	1.012	17.68%
B07	3.833	3.64	0.194	0.048	0.34	27.6%
H09	5.699	5.363	0.336	0.056	0.615	7.53%
L11	6.923	4.33	2.593	2.137	3.05	2.82%
P08	5.99	5.39	0.6	0.36	0.839	10.24%
S10	5.225	5.242	−0.016	−0.281	0.248	8.41%
U01	8.254	6.455	1.799	1.455	2.143	4.99%
U02	9.017	6.999	2.018	1.59	2.446	3.22%
U03	8.546	7.77	0.776	0.356	1.197	3.33%
**Meta analysis**			**0.476**	**0.4**	**0.553**	

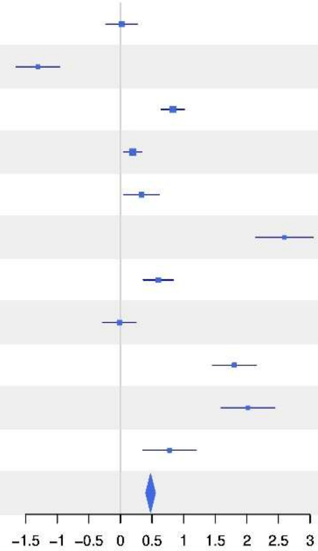

Gamma with Coverage = 0.95

Site	FD(q=2) Enhanced	FD(q=2) Control	Difference	LCL	UCL	W(fixed)
B04	3.436	3.592	−0.156	−0.29	−0.022	11.29%
B05	3.952	5.497	−1.545	−1.712	−1.378	7.31%
B06	3.531	2.728	0.803	0.704	0.902	20.82%
B07	2.616	2.735	−0.119	−0.21	−0.028	24.51%
H09	4.539	4.259	0.28	0.108	0.451	6.92%
L11	4.726	3.14	1.586	1.284	1.888	2.23%
P08	4.039	3.898	0.141	−0.035	0.316	6.62%
S10	3.976	4.005	−0.03	−0.199	0.14	7.06%
U01	6.119	4.825	1.294	1.086	1.502	4.7%
U02	6.862	5.356	1.506	1.297	1.715	4.67%
U03	6.328	6.076	0.252	0.023	0.481	3.87%
**Meta analysis**			**0.21**	**0.165**	**0.256**	

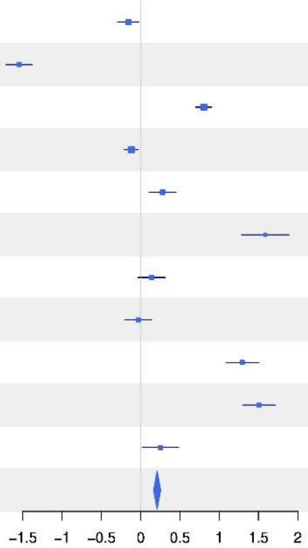

1−S with Coverage = 0.95

Site	FD(q=0) Enhanced	FD(q=0) Control	Difference	LCL	UCL	W(fixed)
B04	−0.005	0.003	−0.008	−0.092	0.075	5.59%
B05	−0.002	0.008	−0.01	−0.093	0.072	5.69%
B06	0.004	0.004	0	−0.083	0.084	5.58%
B07	0.003	0.016	−0.013	−0.103	0.076	4.83%
H09	0.005	0.002	0.003	−0.092	0.098	4.33%
L11	0.011	−0.011	0.022	−0.089	0.133	3.17%
P08	0.008	−0.014	0.023	−0.053	0.099	6.7%
S10	0.007	0.013	−0.006	−0.092	0.08	5.25%
U01	0.024	0.025	−0.001	−0.049	0.047	16.95%
U02	0.028	0.013	0.015	−0.025	0.056	23.62%
U03	0.023	0.007	0.016	−0.03	0.062	18.29%
**Meta analysis**			**0.007**	**−0.013**	**0.026**	

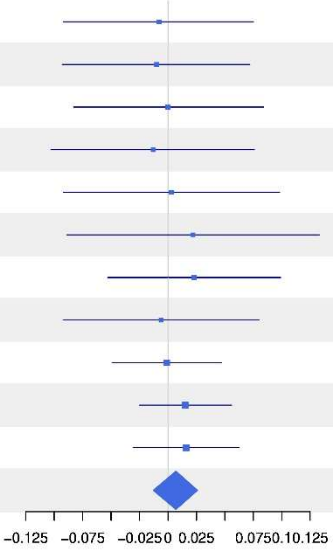

1−S with Coverage = 0.95

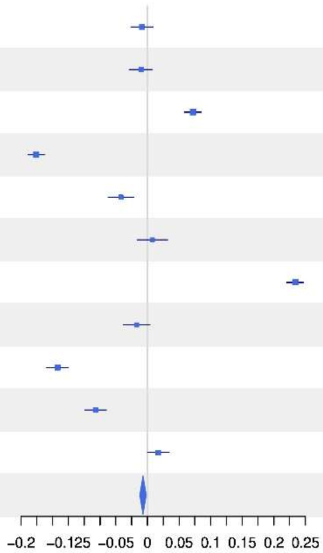

Site	FD(q=1) Enhanced	FD(q=1) Control	Difference	LCL	UCL	W(fixed)
B04	0.046	0.055	−0.009	−0.026	0.009	7.95%
B05	0.047	0.057	−0.01	−0.029	0.008	7.02%
B06	0.098	0.026	0.072	0.059	0.085	14.67%
B07	0.05	0.226	−0.176	−0.189	−0.163	15.39%
H09	0.027	0.069	−0.042	−0.062	−0.022	6%
L11	0.052	0.043	0.008	−0.016	0.032	4.19%
P08	0.289	0.056	0.234	0.22	0.247	13.79%
S10	0.051	0.068	−0.017	−0.038	0.004	5.45%
U01	0.079	0.222	−0.142	−0.159	−0.126	9%
U02	0.109	0.191	−0.082	−0.099	−0.065	8.33%
U03	0.101	0.085	0.017	0	0.034	8.22%
**Meta analysis**			**−0.007**	**−0.012**	**−0.002**	

1−S with Coverage = 0.95

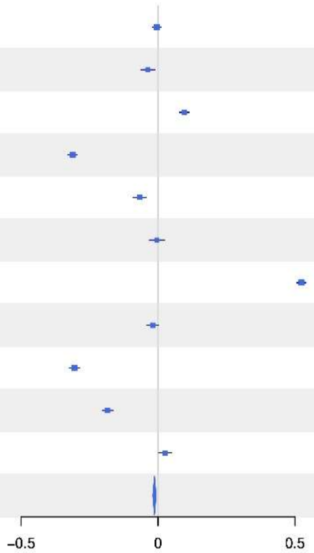

Site	FD(q=2) Enhanced	FD(q=2) Control	Difference	LCL	UCL	W(fixed)
B04	0.073	0.077	−0.004	−0.02	0.012	14.02%
B05	0.085	0.122	−0.037	−0.063	−0.011	5.44%
B06	0.14	0.044	0.096	0.078	0.114	11.28%
B07	0.081	0.393	−0.312	−0.329	−0.295	12.29%
H09	0.043	0.11	−0.067	−0.091	−0.043	6.24%
L11	0.079	0.084	−0.004	−0.032	0.024	4.65%
P08	0.621	0.097	0.524	0.507	0.54	12.96%
S10	0.088	0.107	−0.019	−0.041	0.002	7.82%
U01	0.136	0.441	−0.305	−0.324	−0.286	10.14%
U02	0.189	0.372	−0.184	−0.204	−0.163	8.57%
U03	0.179	0.153	0.026	0.003	0.05	6.61%
**Meta analysis**			**−0.013**	**−0.019**	**−0.007**	

Table S1: Results of linear mixed effects interaction models of the β-deviation, i.e. the difference between β-diversity of observed vs. null-model data (with randomized distribution of individuals across patches in each site) for taxonomic (TD) and functional beta diversity (FD) in combination with Hill numbers q=0, 1, 2 (rare, common, and dominant species). As predictors we used the structural difference between patches (DistTreat), the difference in space (DistXY), and the difference in abiotic conditions (DistPlant) extracted from vegetation indicator values in interaction with manipulated deadwood and canopy structure (TreatC vs. TreatE, i.e. control vs. enhanced), as well as Site as random factor.

Diversity facet	TD	TD	TD	FD	FD	FD
Hill number q	0	1	2	0	1	2
TreatE	0.975	-1.237	**-4.808**	-0.168	**-4.900**	**-5.947**
DistTreat	**3.753**	**2.731**	0.204	-0.111	-1.010	-1.308
DistXY	1.169	**5.816**	**4.578**	0.725	**3.268**	**3.257**
DistPlant	0.085	-1.501	**-2.039**	1.127	**-3.717**	**-3.571**
TreatE:DistTreat	-0.727	**4.298**	**5.276**	**2.147**	**2.496**	**3.032**
TreatE:DistXY	-0.187	**-2.218**	-1.199	-0.502	-0.473	-0.121
TreatE:DistPlant	0.468	**2.449**	**4.745**	**-2.294**	**4.861**	**5.388**

Figure S19: Observed species abundance distribution in control (left) and ESBC districts (right) for low abundance between 1 and 20 individuals, aggregated over all districts.

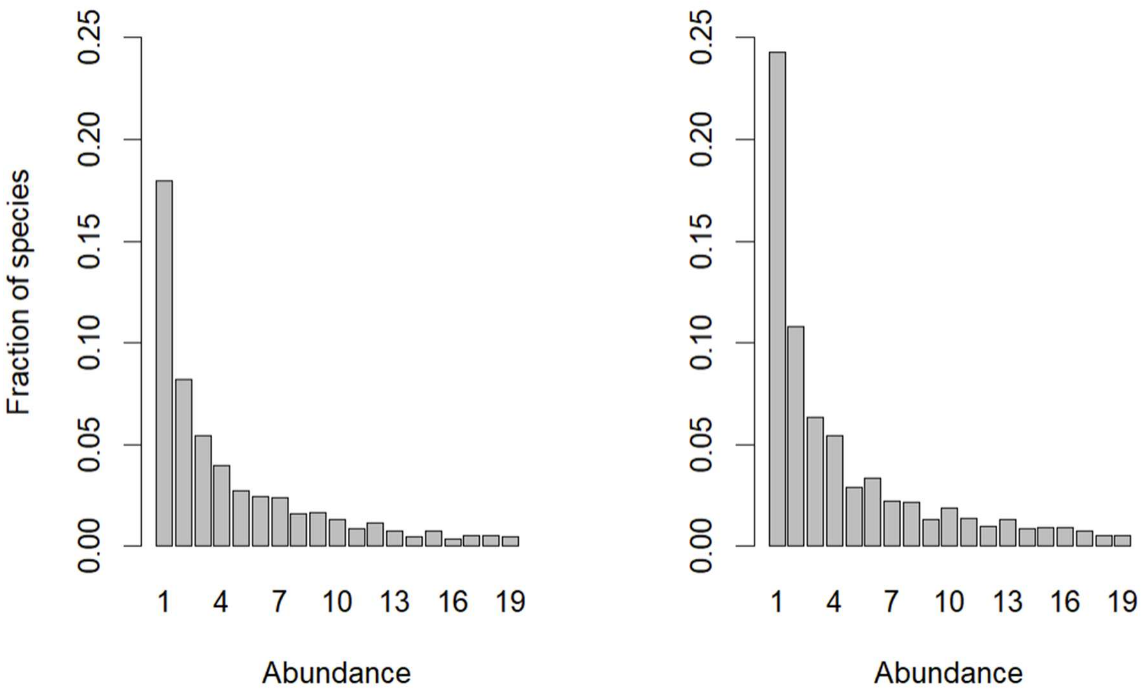

Figure S20: Standardized effect sizes of mean pairwise distances in functional β-diversity of control and enhanced patches in response to treatment categories for rare (q=0), common (q=1), and dominant (q=2) species. P-values for intercept and ESBC treatment indicate if SES values in general significantly differ from 0 and if ESBC differs from Control in a generalized mixed effect model of SES in response to treatment. Percentage values represent the fraction of replicates above (red) or below (below) the vertical 1.96 lines.

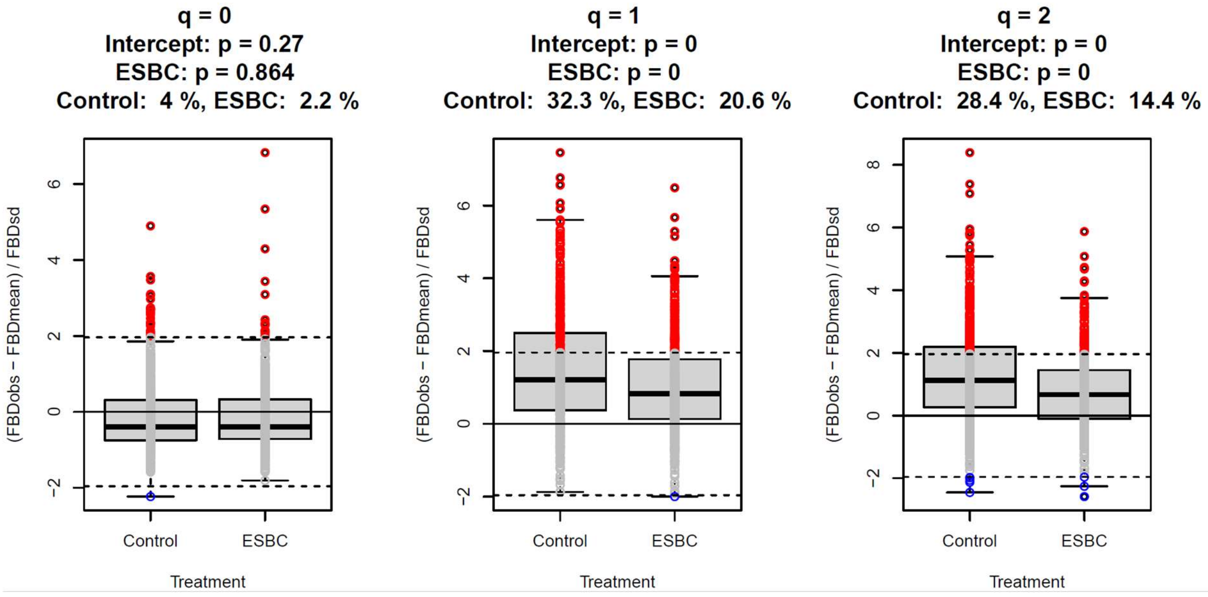

Table S2: Species list and aggregated abundance and patch frequencies in ESBC and control districts

Lucht Code	Familia	Species	Control Individuals	ESBC Individuals	Control Frequencies	ESBC Frequencies
01-.002-.001-.	Carabidae	*Calosoma inquisitor*	2		2	
01-.004-.001-.	Carabidae	*Carabus coriaceus*	260	530	44	44
01-.004-.003-.	Carabidae	*Carabus irregularis*		1		1
01-.004-.007-.	Carabidae	*Carabus violaceus*	344	366	32	67
01-.004-.008-.	Carabidae	*Carabus intricatus*	20	19	7	6
01-.004-.009-.	Carabidae	*Carabus auronitens*	403	289	58	53
01-.004-.010-.	Carabidae	*Carabus problematicus*	76	84	14	15
01-.004-.012-.	Carabidae	*Carabus granulatus*	2	11	2	2
01-.004-.017-.	Carabidae	*Carabus convexus*		2		1
01-.004-.020-.	Carabidae	*Carabus ulrichii*	2	1	2	1
01-.004-.021-.	Carabidae	*Carabus arcensis*	4	29	3	8
01-.004-.026-.	Carabidae	*Carabus nemoralis*	646	594	65	59
01-.004-.028-.	Carabidae	*Carabus hortensis*	6	7	3	4
01-.004-.029-.	Carabidae	*Carabus glabratus*	119	130	48	36
01-.004-.030-.	Carabidae	*Carabus linnei*	220	25	26	10
01-.004-.033-.	Carabidae	*Carabus sylvestris*	7	11	4	7
01-.005-.003-.	Carabidae	*Cychrus caraboides*		2		2
01-.005-.004-.	Carabidae	*Cychrus attenuatus*	61	90	21	31
01-.006-.002-.	Carabidae	*Leistus rufomarginatus*	13	6	13	6
01-.007-.006-.	Carabidae	*Nebria brevicollis*	67	20	23	7
01-.009-.003-.	Carabidae	*Notiophilus palustris*	6	39	6	9
01-.009-.008-.	Carabidae	*Notiophilus biguttatus*	109	109	43	41
01-.012-.002-.	Carabidae	*Elaphrus cupreus*		1		1
01-.013-.001-.	Carabidae	*Loricera pilicornis*	3	8	3	8
01-.015-.001-.	Carabidae	*Clivina fossor*		1		1
01-.021-.006-.	Carabidae	*Trechus quadristriatus*	12	43	10	16
01-.021-.007-.	Carabidae	*Trechus obtusus*	1		1	
01-.0271.001-.	Carabidae	*Paratachys bistriatus*	7	78	4	17
01-.028-.001-.	Carabidae	*Tachyta nana*		1		1
01-.029-.010-.	Carabidae	*Bembidion lampros*	4	18	4	8
01-.029-.011-.	Carabidae	*Bembidion properans*		2		2
01-.029-.042-.	Carabidae	*Bembidion deletum*	1	2	1	2
01-.029-.054-.	Carabidae	*Bembidion tetracolum*	1		1	
01-.029-.090-.	Carabidae	*Bembidion quadrimaculatum*		5		5
01-.029-.095-.	Carabidae	*Bembidion obtusum*		3		2
01-.029-.101-.	Carabidae	*Bembidion mannerheimii*		3		2
01-.029-.102-.	Carabidae	*Bembidion guttula*		2		2
01-.029-.103-.	Carabidae	*Bembidion lunulatum*		1		1
01-.030-.004-.	Carabidae	*Asaphidion flavipes*	3	2	3	2
01-.037-.001-.	Carabidae	*Anisodactylus binotatus*		1		1
01-.038-.001-.	Carabidae	*Diachromus germanus*	1		1	
01-.039-.001-.	Carabidae	*Trichotichnus laevicollis*	1	13	1	11
01-.039-.002-.	Carabidae	*Trichotichnus nitens*	5		4	
01-.041-.020-.	Carabidae	*Harpalus signaticornis*	8	17	6	10
01-.041-.021-.	Carabidae	*Harpalus rufipes*		18		11
01-.041-.022-.	Carabidae	*Harpalus griseus*	1		1	
01-.041-.030-.	Carabidae	*Harpalus affinis*	1	5	1	4

01-.041-.031-.	Carabidae	*Harpalus distinguendus*		4		4
01-.041-.040-.	Carabidae	*Harpalus atratus*	5	10	4	5
01-.041-.044-.	Carabidae	*Harpalus progrediens*		1		1
01-.041-.045-.	Carabidae	*Harpalus latus*	12	32	10	22
01-.041-.047-.	Carabidae	*Harpalus laevipes*		9		7
01-.041-.049-.	Carabidae	*Harpalus rubripes*		1		1
01-.0411.005-.	Carabidae	*Ophonus ardosiacus*	4	24	3	15
01-.0411.008-.	Carabidae	*Ophonus azureus*		4		4
01-.0411.010-.	Carabidae	*Ophonus schaubergerianus*		21		4
01-.0411.012-.	Carabidae	*Ophonus laticollis*	11	28	8	9
01-.0411.014-.	Carabidae	*Ophonus puncticollis*		1		1
01-.0411.017-.	Carabidae	*Ophonus puncticeps*		4		2
01-.045-.005-.	Carabidae	*Bradycellus harpalinus*		4		3
01-.046-.004-.	Carabidae	*Acupalpus meridianus*	4	15	4	10
01-.046-.008-.	Carabidae	*Acupalpus dubius*	6	3	6	3
01-.046-.010-.	Carabidae	*Acupalpus exiguus*		2		1
01-.047-.001-.	Carabidae	*Anthracus consputus*	1	1	1	1
01-.049-.001-.	Carabidae	*Stomis pumicatus*	2	1	2	1
01-.050-.007-.	Carabidae	*Poecilus cupreus*	1	13	1	9
01-.050-.008-.	Carabidae	*Poecilus versicolor*		3		3
01-.051-.005-.	Carabidae	*Pterostichus pumilio*	13	7	9	7
01-.051-.012-.	Carabidae	*Pterostichus diligens*	1	2	1	2
01-.051-.013-.	Carabidae	*Pterostichus ovoideus*	7	11	7	9
01-.051-.015-.	Carabidae	*Pterostichus vernalis*		1		1
01-.051-.022-.	Carabidae	*Pterostichus minor*	1	1	1	1
01-.051-.024-.	Carabidae	*Pterostichus oblongopunctatus*	1076	712	90	71
01-.051-.026-.	Carabidae	*Pterostichus niger*	545	144	49	44
01-.051-.027-.	Carabidae	*Pterostichus melanarius*	62	39	22	15
01-.051-.030-.	Carabidae	*Pterostichus madidus*	5		3	
01-.051-.031-.	Carabidae	*Pterostichus aethiops*	3		3	
01-.051-.035-.	Carabidae	*Pterostichus melas*	52	75	8	9
01-.051-.039-.	Carabidae	*Pterostichus burmeisteri*	589	370	45	43
01-.051-.057-.	Carabidae	*Pterostichus cristatus*		1		1
01-.052-.001-.	Carabidae	*Molops elatus*	82	30	25	15
01-.052-.002-.	Carabidae	*Molops piceus*	112	118	31	35
01-.053-.002-.	Carabidae	*Abax parallelepipedus*	1472	872	111	95
01-.053-.004-.	Carabidae	*Abax parallelus*	469	194	63	52
01-.053-.005-.	Carabidae	*Abax ovalis*	101	76	9	13
01-.053-.006-.	Carabidae	*Abax carinatus*	1	6	1	3
01-.055-.001-.	Carabidae	*Synuchus vivalis*	4		3	
01-.056-.005-.	Carabidae	*Calathus micropterus*	3		2	
01-.056-.008-.	Carabidae	*Calathus rotundicollis*	1	1	1	1
01-.062-.009-.	Carabidae	*Agonum muelleri*		1		1
01-.062-.012-.	Carabidae	*Agonum viduum*		3		1
01-.0622.001-.	Carabidae	*Anchomenus dorsalis*	1	28	1	9
01-.0631.003-.	Carabidae	*Limodromus assimilis*	59	6	17	2
01-.065-.001-.	Carabidae	*Amara plebeja*		3		3

01-.065-.008-.	Carabidae	*Amara similata*		1		1
01-.065-.009-.	Carabidae	*Amara ovata*	38	130	18	32
01-.065-.013-.	Carabidae	*Amara convexior*	2	8	2	5
01-.065-.014-.	Carabidae	*Amara communis*		7		5
01-.065-.017-.	Carabidae	*Amara curta*		1		1
01-.065-.018-.	Carabidae	*Amara lunicollis*	3	3	3	3
01-.065-.021-.	Carabidae	*Amara aenea*		6		6
01-.065-.026-.	Carabidae	*Amara familiaris*	19	89	14	36
01-.065-.028-.	Carabidae	*Amara lucida*		1		1
01-.065-.057-.	Carabidae	*Amara aulica*	1	1	1	1
01-.070-.002-.	Carabidae	*Badister bullatus*		1		1
01-.070-.003-.	Carabidae	*Badister lacertosus*		1		1
01-.071-.001-.	Carabidae	*Panagaeus cruxmajor*		1		1
01-.076-.001-.	Carabidae	*Demetrias atricapillus*		1		1
01-.079-.004-.	Carabidae	*Dromius agilis*	1	1	1	1
01-.079-.010-.	Carabidae	*Dromius fenestratus*		2		2
01-.079-.012-.	Carabidae	*Dromius quadrimaculatus*	2	1	2	1
01-.082-.001-.	Carabidae	*Microlestes minutulus*		1		1
01-.086-.001-.	Carabidae	*Brachinus crepitans*		6		3
01-.086-.003-.	Carabidae	*Brachinus explodens*	3	2	2	2
04-.008-.027-.	Dytiscidae	*Hydroporus memnonius*	3	3	3	3
04-.023-.008-.	Dytiscidae	*Agabus melanarius*	14	78	11	29
09-.0011.009-.	Helophoridae	*Helophorus aquaticus*		2		2
09-.0011.022-.	Helophoridae	*Helophorus flavipes*	15	12	13	9
09-.002-.001-.	Hydrophilidae	*Sphaeridium bipustulatum*		1		1
09-.003-.011-.	Hydrophilidae	*Cercyon lateralis*		1		1
09-.003-.012-.	Hydrophilidae	*Cercyon laminatus*	1	3	1	3
09-.003-.014-.	Hydrophilidae	*Cercyon quisquilius*		1		1
09-.003-.017-.	Hydrophilidae	*Cercyon pygmaeus*		1		1
09-.004-.001-.	Hydrophilidae	*Megasternum concinnum*	10	14	10	9
09-.005-.001-.	Hydrophilidae	*Cryptopleurum minutum*	2		2	
09-.008-.0011.	Hydrophilidae	*Hydrobius fuscipes*	13	8	8	7
09-.010-.001-.	Hydrophilidae	*Anacaena globulus*	3	4	3	4
10-.002-.002-.	Histeridae	*Plegaderus vulneratus*		1		1
10-.002-.004-.	Histeridae	*Plegaderus dissectus*	2	4	2	4
10-.005-.003-.	Histeridae	*Abraeus perpusillus*	1		1	
10-.0071.001-.	Histeridae	*Aeletes atomarius*	2		2	
10-.009-.004-.	Histeridae	*Gnathoncus buyssoni*		3		3
10-.016-.001-.	Histeridae	*Dendrophilus punctatus*	1		1	
10-.020-.001-.	Histeridae	*Paromalus flavicornis*	8	2	8	2
10-.020-.002-.	Histeridae	*Paromalus parallelepipedus*	12	8	10	6
10-.029-.006-.	Histeridae	*Margarinotus carbonarius*		1		1
10-.029-.008-.	Histeridae	*Margarinotus striola*	28	3	10	3
10-.029-.012-.	Histeridae	*Margarinotus brunneus*	1		1	
10-.029-.013-.	Histeridae	*Margarinotus marginatus*	1	1	1	1

10-.032-.001-.	Histeridae	*Hister quadrimaculatus*		1		1
11-.001-.001-.	Sphaeritidae	*Sphaerites glabratus*	1	4	1	3
12-.001-.002-.	Silphidae	*Nicrophorus humator*	32	11	18	9
12-.001-.004-.	Silphidae	*Nicrophorus investigator*	121	18	9	10
12-.001-.006-.	Silphidae	*Nicrophorus vespilloides*	3452	1389	90	93
12-.001-.008-.	Silphidae	*Nicrophorus vespillo*	200	76	43	23
12-.002-.001-.	Silphidae	*Necrodes littoralis*	1	1	1	1
12-.003-.002-.	Silphidae	*Thanatophilus sinuatus*		1		1
12-.004-.001-.	Silphidae	*Oiceoptoma thoracicum*	299	22	24	15
12-.006-.001-.	Silphidae	*Dendroxena quadrimaculata*	24	50	16	23
12-.007-.004-.	Silphidae	*Silpha obscura*		1		1
12-.007-.005-.	Silphidae	*Silpha tristis*		1		1
12-.009-.001-.	Silphidae	*Phosphuga atrata*	346	262	53	52
13-.001-.001-.	Leiodidae	*Leptinus testaceus*		1		1
14-.001-.001-.	Leiodidae	*Ptomaphagus varicornis*	123	194	35	37
14-.001-.004-.	Leiodidae	*Ptomaphagus sericatus*	5	5	4	3
14-.005-.001-.	Leiodidae	*Nargus velox*	32	8	6	6
14-.005-.003-.	Leiodidae	*Nargus wilkini*	20	35	14	17
14-.005-.005-.	Leiodidae	*Nargus anisotomoides*	6	11	5	9
14-.006-.008-.	Leiodidae	*Choleva reitteri*	1		1	
14-.006-.009-.	Leiodidae	*Choleva cisteloides*	4	9	3	6
14-.006-.011-.	Leiodidae	*Choleva glauca*	10	2	6	2
14-.006-.013-.	Leiodidae	*Choleva angustata*	15	13	12	8
14-.010-.001-.	Leiodidae	*Sciodrepoides watsoni*	528	564	49	64
14-.010-.002-.	Leiodidae	*Sciodrepoides fumatus*	6	6	4	3
14-.011-.001-.	Leiodidae	*Catops subfuscus*	17	4	5	4
14-.011-.002-.	Leiodidae	*Catops longulus*		1		1
14-.011-.003-.	Leiodidae	*Catops coracinus*	29	36	18	16
14-.011-.007-.	Leiodidae	*Catops tristis*		4		4
14-.011-.017-.	Leiodidae	*Catops fuliginosus*	1	7	1	7
14-.011-.020-.	Leiodidae	*Catops picipes*	8	15	6	12
14-.0111.001-.	Leiodidae	*Apocatops nigrita*	253	367	62	72
15-.001-.001-.	Leiodidae	*Colon latum*	3	5	3	4
15-.001-.012-.	Leiodidae	*Colon dentipes*		1		1
15-.001-.015-.	Leiodidae	*Colon brunneum*		1		1
16-.003-.0131.	Leiodidae	*Leiodes oblonga*	2	4	2	4
16-.004-.001-.	Leiodidae	*Colenis immunda*	2	2	2	2
16-.007-.001-.	Leiodidae	*Anisotoma humeralis*	12	34	11	24
16-.007-.003-.	Leiodidae	*Anisotoma castanea*	2	2	2	2
16-.007-.005-.	Leiodidae	*Anisotoma orbicularis*	2	7	2	6
16-.009-.001-.	Leiodidae	*Amphicyllis globus*	21	24	16	16
16-.009-.002-.	Leiodidae	*Amphicyllis globiformis*		2		2
16-.011-.003-.	Leiodidae	*Agathidium varians*	2	1	2	1
16-.011-.007-.	Leiodidae	*Agathidium rotundatum*		1		1
16-.011-.008-.	Leiodidae	*Agathidium confusum*	11	3	10	3
16-.011-.010-.	Leiodidae	*Agathidium nigrinum*		2		2
16-.011-.013-.	Leiodidae	*Agathidium nigripenne*	3	1	3	1

16-.011-.014-.	Leiodidae	*Agathidium atrum*	1	5	1	5
16-.011-.015-.	Leiodidae	*Agathidium seminulum*	58	179	36	74
16-.011-.016-.	Leiodidae	*Agathidium laevigatum*		1		1
16-.011-.017-.	Leiodidae	*Agathidium bohemicum*	7	4	4	4
16-.011-.019-.	Leiodidae	*Agathidium pisanum*	1		1	
18-.004-.006-.	Staphylinidae	*Cephennium gallicum*	10	3	4	3
18-.005-.001-.	Staphylinidae	*Neuraphes elongatulus*	7	29	6	25
18-.005-.007-.	Staphylinidae	*Neuraphes rubicundus*		1		1
18-.005-.009-.	Staphylinidae	*Neuraphes ruthenus*	2		2	
18-.005-.020-.	Staphylinidae	*Neuraphes parallelus*		13		11
18-.005-.029-.	Staphylinidae	*Neuraphes coecus*	1		1	
18-.006-.003-.	Staphylinidae	*Scydmoraphes helvolus*		3		3
18-.007-.003-.	Staphylinidae	*Stenichnus scutellaris*	9	12	7	10
18-.007-.005-.	Staphylinidae	*Stenichnus godarti*	7	24	7	16
18-.007-.008-.	Staphylinidae	*Stenichnus collaris*	9	4	9	4
18-.007-.010-.	Staphylinidae	*Stenichnus bicolor*	1	4	1	4
18-.008-.002-.	Staphylinidae	*Microscydmus minimus*	5	17	4	15
18-.009-.014-.	Staphylinidae	*Euconnus claviger*		2		2
18-.009-.015-.	Staphylinidae	*Euconnus pragensis*	1		1	
21-.002-.002-.	Ptiliidae	*Ptenidium laevigatum*		1		1
21-.002-.003-.	Ptiliidae	*Ptenidium turgidum*	2	4	2	4
21-.017-.001-.	Ptiliidae	*Baeocrara variolosa*	4	7	4	7
21-.019-.015-.	Ptiliidae	*Acrotrichis intermedia*	53	85	36	45
21-.019-.019-.	Ptiliidae	*Acrotrichis sitkaensis*		1		1
23-.002-.001-.	Staphylinidae	*Siagonium quadricorne*	2		2	
23-.0022.001-.	Staphylinidae	*Scaphidium quadrimaculatum*	6	10	6	9
23-.0023.001-.	Staphylinidae	*Scaphisoma agaricinum*	4	24	4	17
23-.0023.006-.	Staphylinidae	*Scaphisoma subalpinum*	1		1	
23-.0023.008-.	Staphylinidae	*Scaphisoma obenbergeri*		2		2
23-.005-.001-.	Staphylinidae	*Phloeocharis subtilissima*	1		1	
23-.0061.001-.	Staphylinidae	*Dasycerus sulcatus*	1		1	
23-.009-.001-.	Staphylinidae	*Proteinus ovalis*	3		3	
23-.009-.004-.	Staphylinidae	*Proteinus brachypterus*		2		2
23-.009-.005-.	Staphylinidae	*Proteinus atomarius*	22	11	12	5
23-.0091.002-.	Staphylinidae	*Micropeplus staphylinoides*	2		2	
23-.010-.010-.	Staphylinidae	*Eusphalerum longipenne*	3	18	3	13
23-.010-.013-.	Staphylinidae	*Eusphalerum stramineum*		5		4
23-.010-.014-.	Staphylinidae	*Eusphalerum primulae*		3		3
23-.010-.021-.	Staphylinidae	*Eusphalerum semicoleoptratum*		1		1
23-.010-.022-.	Staphylinidae	*Eusphalerum luteum*	5	8	1	7
23-.010-.023-.	Staphylinidae	*Eusphalerum marshami*	1		1	

23-.010-.024-.	Staphylinidae	*Eusphalerum signatum*	45	12	20	10
23-.010-.025-.	Staphylinidae	*Eusphalerum limbatum*	172	265	32	37
23-.010-.029-.	Staphylinidae	*Eusphalerum rectangulum*	17	1319	11	43
23-.010-.031-.	Staphylinidae	*Eusphalerum sorbi*	1	2	1	2
23-.010-.033-.	Staphylinidae	*Eusphalerum atrum*	3	3	2	3
23-.010-.034-.	Staphylinidae	*Eusphalerum tenenbaumi*		3		3
23-.011-.001-.	Staphylinidae	*Acrulia inflata*		5		5
23-.0141.001-.	Staphylinidae	*Hapalaraea pygmaea*	1		1	
23-.0143.004-.	Staphylinidae	*Dropephylla ioptera*	4	3	3	2
23-.015-.005-.	Staphylinidae	*Omalium rivulare*	35	13	24	13
23-.015-.018-.	Staphylinidae	*Omalium caesum*		8		8
23-.015-.019-.	Staphylinidae	*Omalium rugatum*	1	3	1	3
23-.016-.005-.	Staphylinidae	*Phloeonomus pusillus*	2	4	2	3
23-.016-.006-.	Staphylinidae	*Phloeonomus punctipennis*		1		1
23-.0162.001-.	Staphylinidae	*Phloeostiba plana*	1	1	1	1
23-.0162.002-.	Staphylinidae	*Phloeostiba lapponica*		1		1
23-.025-.001-.	Staphylinidae	*Anthobium melanocephalum*	1		1	
23-.025-.002-.	Staphylinidae	*Anthobium atrocephalum*	87	60	38	25
23-.030-.001-.	Staphylinidae	*Acidota crenata*	3	1	3	1
23-.030-.003-.	Staphylinidae	*Acidota cruentata*	2		2	
23-.031-.001-.	Staphylinidae	*Amphichroum canaliculatum*	315	347	38	41
23-.032-.003-.	Staphylinidae	*Lesteva longoelytrata*	125	147	31	34
23-.035-.013-.	Staphylinidae	*Anthophagus angusticollis*	7	23	7	10
23-.035-.014-.	Staphylinidae	*Anthophagus omalinus*	1		1	
23-.035-.017-.	Staphylinidae	*Anthophagus alpestris*	2	11	2	3
23-.037-.003-.	Staphylinidae	*Coryphium angusticolle*		1		1
23-.040-.001-.	Staphylinidae	*Syntomium aeneum*		1		1
23-.042-.001-.	Staphylinidae	*Coprophilus striatulus*	1	4	1	4
23-.044-.002-.	Staphylinidae	*Planeustomus palpalis*	1		1	
23-.046-.006-.	Staphylinidae	*Carpelimus bilineatus*	1		1	
23-.046-.017-.	Staphylinidae	*Carpelimus corticinus*	6	7	6	7
23-.046-.032-.	Staphylinidae	*Carpelimus elongatulus*		2		1
23-.0481.001-.	Staphylinidae	*Anotylus insecatus*	6	8	4	7
23-.0481.003-.	Staphylinidae	*Anotylus rugosus*	3	19	3	15
23-.0481.006-.	Staphylinidae	*Anotylus inustus*		1		1
23-.0481.007-.	Staphylinidae	*Anotylus sculpturatus*	130	198	30	33
23-.0481.008-.	Staphylinidae	*Anotylus mutator*	21	148	11	22
23-.0481.011-.	Staphylinidae	*Anotylus nitidulus*	2	4	2	4
23-.0481.022-.	Staphylinidae	*Anotylus tetracarinatus*	24	20	20	19

23-.049-.008-.	Staphylinidae	*Platystethus nitens*	4	4	4	4
23-.055-.006-.	Staphylinidae	*Stenus fossulatus*	3	4	3	4
23-.055-.022-.	Staphylinidae	*Stenus clavicornis*	1	9	1	4
23-.055-.026-.	Staphylinidae	*Stenus bimaculatus*		1		1
23-.055-.041-.	Staphylinidae	*Stenus canaliculatus*		1		1
23-.055-.094-.	Staphylinidae	*Stenus impressus*	1	3	1	3
23-.055-.097-.	Staphylinidae	*Stenus fuscicornis*		1		1
23-.061-.002-.	Staphylinidae	*Rugilus subtilis*	5	5	5	5
23-.061-.003-.	Staphylinidae	*Rugilus rufipes*	8	25	8	20
23-.061-.007-.	Staphylinidae	*Rugilus mixtus*	2	6	2	5
23-.062-.004-.	Staphylinidae	*Medon brunneus*	1	3	1	2
23-.066-.001-.	Staphylinidae	*Scopaeus laevigatus*	2	7	2	7
23-.067-.001-.	Staphylinidae	*Domene scabricollis*	9	6	9	5
23-.0674.011-.	Staphylinidae	*Tetartopeus terminatus*		1		1
23-.068-.017-.	Staphylinidae	*Lathrobium geminum*	4	7	3	5
23-.068-.021-.	Staphylinidae	*Lathrobium fulvipenne*	6	4	5	3
23-.068-.023-.	Staphylinidae	*Lathrobium brunnipes*	5		5	
23-.068-.030-.	Staphylinidae	*Lathrobium pallidum*		2		2
23-.075-.006-.	Staphylinidae	*Leptacinus pusillus*		1		1
23-.078-.001-.	Staphylinidae	*Nudobius lentus*	2	1	2	1
23-.079-.001-.	Staphylinidae	*Gyrohypnus punctulatus*		1		1
23-.079-.005-.	Staphylinidae	*Gyrohypnus angustatus*	1		1	
23-.080-.005-.	Staphylinidae	*Xantholinus tricolor*	26	7	16	6
23-.080-.010-.	Staphylinidae	*Xantholinus linearis*	16	16	11	14
23-.080-.015-.	Staphylinidae	*Xantholinus longiventris*		13		11
23-.0801.001-.	Staphylinidae	*Hypnogyra angularis*	1	1	1	1
23-.081-.001-.	Staphylinidae	*Atrecus affinis*	4	10	4	8
23-.082-.001-.	Staphylinidae	*Othius punctulatus*	93	65	54	37
23-.082-.005-.	Staphylinidae	*Othius subuliformis*	10	10	9	6
23-.088-.002-.	Staphylinidae	*Philonthus laevicollis*	6		4	
23-.088-.011-.	Staphylinidae	*Philonthus atratus*	1		1	
23-.088-.016-.	Staphylinidae	*Philonthus corruscus*		1		1
23-.088-.021-.	Staphylinidae	*Philonthus tenuicornis*	6	11	6	11
23-.088-.023-.	Staphylinidae	*Philonthus cognatus*	6	14	6	11
23-.088-.025-.	Staphylinidae	*Philonthus politus*	30	19	19	12
23-.088-.026-.	Staphylinidae	*Philonthus succicola*	12	10	8	10
23-.088-.027-.	Staphylinidae	*Philonthus addendus*		2		2
23-.088-.029-.	Staphylinidae	*Philonthus decorus*	412	226	68	52
23-.088-.058-.	Staphylinidae	*Philonthus sanguinolentus*	1	1	1	1
23-.0882.007-.	Staphylinidae	*Bisnius pseudoparcus*	1		1	
23-.0882.010-.	Staphylinidae	*Bisnius fimetarius*	20	11	14	11
23-.090-.002-.	Staphylinidae	*Gabrius femoralis*		5		5
23-.090-.004-.	Staphylinidae	*Gabrius tirolensis*	1		1	
23-.090-.005-.	Staphylinidae	*Gabrius astutus*	1	1	1	1
23-.090-.009-.	Staphylinidae	*Gabrius splendidulus*	28	63	23	46
23-.090-.024-.	Staphylinidae	*Gabrius appendiculatus*		2		2
23-.092-.001-.	Staphylinidae	*Ontholestes tessellatus*	3		1	
23-.092-.002-.	Staphylinidae	*Ontholestes murinus*	1		1	
23-.095-.001-.	Staphylinidae	*Platydracus fulvipes*	3	30	3	18

23-.095-.002-.	Staphylinidae	*Platydracus latebricola*	5	10	3	7
23-.095-.003-.	Staphylinidae	*Platydracus chalcocephalus*	530	577	40	33
23-.095-.005-.	Staphylinidae	*Platydracus stercorarius*		4		4
23-.096-.003-.	Staphylinidae	*Dinothenarus fossor*		2		2
23-.099-.001-.	Staphylinidae	*Ocypus olens*	3	4	2	3
23-.099-.0021.	Staphylinidae	*Ocypus pedemontanus*	5	8	4	6
23-.099-.010-.	Staphylinidae	*Ocypus nitens*	34	44	22	24
23-.099-.012-.	Staphylinidae	*Ocypus brunnipes*	1		1	
23-.0991.003-.	Staphylinidae	*Tasgius morsitans*	6	3	4	3
23-.0991.006-.	Staphylinidae	*Tasgius winkleri*	15	16	9	13
23-.0991.007-.	Staphylinidae	*Tasgius melanarius*	4	1	4	1
23-.100-.002-.	Staphylinidae	*Heterothops praevius*	1	1	1	1
23-.104-.0001.	Staphylinidae	*Quedius dilatatus*		1		1
23-.104-.001-.	Staphylinidae	*Quedius brevis*	1		1	
23-.104-.005-.	Staphylinidae	*Quedius lateralis*	7	4	6	3
23-.104-.016-.	Staphylinidae	*Quedius mesomelinus*	16	48	15	28
23-.104-.019-.	Staphylinidae	*Quedius xanthopus*	204	85	70	35
23-.104-.020-.	Staphylinidae	*Quedius scitus*	1	1	1	1
23-.104-.022-.	Staphylinidae	*Quedius cinctus*	11	5	8	5
23-.104-.024-.	Staphylinidae	*Quedius plagiatus*		2		2
23-.104-.025-.	Staphylinidae	*Quedius fuliginosus*		1		1
23-.104-.026-.	Staphylinidae	*Quedius curtipennis*		13		7
23-.104-.038-.	Staphylinidae	*Quedius picipes*	1		1	
23-.104-.043-.	Staphylinidae	*Quedius suturalis*		1		1
23-.104-.044-.	Staphylinidae	*Quedius limbatus*	1		1	
23-.104-.048-.	Staphylinidae	*Quedius fumatus*	12	17	11	16
23-.104-.055-.	Staphylinidae	*Quedius lucidulus*	1		1	
23-.104-.061-.	Staphylinidae	*Quedius paradisianus*	3	7	3	5
23-.104-.070-.	Staphylinidae	*Quedius boops*	1	2	1	2
23-.107-.001-.	Staphylinidae	*Habrocerus capillaricornis*	1	1	1	1
23-.108-.001-.	Staphylinidae	*Trichophya pilicornis*	1	1	1	1
23-.109-.002-.	Staphylinidae	*Mycetoporus mulsanti*	1		1	
23-.109-.008-.	Staphylinidae	*Mycetoporus lepidus*	34	84	24	37
23-.109-.0132.	Staphylinidae	*Mycetoporus dispersus*		1		1
23-.109-.015-.	Staphylinidae	*Mycetoporus forticornis*		1		1
23-.109-.017-.	Staphylinidae	*Mycetoporus clavicornis*		1		1
23-.109-.021-.	Staphylinidae	*Mycetoporus niger*	3	2	3	2
23-.109-.027-.	Staphylinidae	*Mycetoporus rufescens*	1	2	1	2
23-.109-.030-.	Staphylinidae	*Mycetoporus punctus*	3		3	
23-.1091.002-.	Staphylinidae	*Ischnosoma longicorne*		1		1
23-.1091.003-.	Staphylinidae	*Ischnosoma splendidum*	10	8	9	7
23-.1101.001-.	Staphylinidae	*Bryophacis crassicornis*		1		1
23-.1101.002-.	Staphylinidae	*Bryophacis rufus*	107	74	33	26
23-.111-.003-.	Staphylinidae	*Lordithon thoracicus*	1	4	1	2

23-.111-.005-.	Staphylinidae	*Lordithon exoletus*	34	18	22	11
23-.111-.006-.	Staphylinidae	*Lordithon trinotatus*		4		3
23-.111-.007-.	Staphylinidae	*Lordithon lunulatus*	18	19	14	14
23-.1111.001-.	Staphylinidae	*Carphacis striatus*	1	1	1	1
23-.112-.002-.	Staphylinidae	*Bolitobius castaneus*	1		1	
23-.1121.003-.	Staphylinidae	*Parabolitobius inclinans*	1	2	1	2
23-.1121.004-.	Staphylinidae	*Parabolitobius formosus*	1	7	1	4
23-.113-.001-.	Staphylinidae	*Sepedophilus littoreus*		1		1
23-.113-.002-.	Staphylinidae	*Sepedophilus testaceus*	4	5	4	5
23-.113-.0022.	Staphylinidae	*Sepedophilus marshami*		1		1
23-.113-.003-.	Staphylinidae	*Sepedophilus immaculatus*		1		1
23-.113-.004-.	Staphylinidae	*Sepedophilus pedicularius*	1	1	1	1
23-.113-.0042.	Staphylinidae	*Sepedophilus obtusus*		1		1
23-.114-.001-.	Staphylinidae	*Tachyporus nitidulus*	2	13	2	8
23-.114-.002-.	Staphylinidae	*Tachyporus obtusus*		2		2
23-.114-.005-.	Staphylinidae	*Tachyporus solutus*	2	1	2	1
23-.114-.007-.	Staphylinidae	*Tachyporus hypnorum*	17	32	12	22
23-.114-.008-.	Staphylinidae	*Tachyporus chrysomelinus*		1		1
23-.114-.0081.	Staphylinidae	*Tachyporus dispar*	2	4	2	4
23-.115-.001-.	Staphylinidae	*Lamprinodes saginatus*		1		1
23-.117-.004-.	Staphylinidae	*Tachinus humeralis*	9	1	7	1
23-.117-.006-.	Staphylinidae	*Tachinus subterraneus*	1	1	1	1
23-.117-.010-.	Staphylinidae	*Tachinus pallipes*	9	6	8	6
23-.117-.012-.	Staphylinidae	*Tachinus fimetarius*		2		2
23-.117-.013-.	Staphylinidae	*Tachinus rufipes*	4	4	4	3
23-.117-.014-.	Staphylinidae	*Tachinus laticollis*	10	12	9	10
23-.117-.020-.	Staphylinidae	*Tachinus elongatus*	26	32	19	21
23-.126-.008-.	Staphylinidae	*Oligota pusillima*		1		1
23-.126-.009-.	Staphylinidae	*Oligota pumilio*	5	2	4	2
23-.1261.001-.	Staphylinidae	*Holobus flavicornis*		2		2
23-.1261.002-.	Staphylinidae	*Holobus apicatus*	1	3	1	3
23-.1262.001-.	Staphylinidae	*Cypha longicornis*	1	4	1	4
23-.129-.001-.	Staphylinidae	*Encephalus complicans*		1		1
23-.130-.004-.	Staphylinidae	*Gyrophaena affinis*		1		1
23-.130-.011-.	Staphylinidae	*Gyrophaena minima*		3		3
23-.130-.022-.	Staphylinidae	*Gyrophaena manca*	1	2	1	2
23-.130-.023-.	Staphylinidae	*Gyrophaena strictula*		1		1
23-.130-.024-.	Staphylinidae	*Gyrophaena polita*		2		2
23-.130-.025-.	Staphylinidae	*Gyrophaena boleti*	2	3	2	3
23-.131-.001-.	Staphylinidae	*Cyphea curtula*		1		1
23-.132-.002-.	Staphylinidae	*Placusa depressa*	2	2	2	2
23-.132-.003-.	Staphylinidae	*Placusa tachyporoides*		1		1
23-.132-.005-.	Staphylinidae	*Placusa atrata*		1		1
23-.134-.001-.	Staphylinidae	*Anomognathus cuspidatus*		1		1
23-.141-.001-.	Staphylinidae	*Leptusa pulchella*	9	24	7	15

23-.141-.006-.	Staphylinidae	*Leptusa ruficollis*	9	11	8	8
23-.142-.001-.	Staphylinidae	*Euryusa castanoptera*	3	5	3	5
23-.142-.006-.	Staphylinidae	*Euryusa pipitzi*		1		1
23-.147-.001-.	Staphylinidae	*Bolitochara obliqua*	2	6	2	6
23-.147-.002-.	Staphylinidae	*Bolitochara bella*		5		5
23-.147-.005-.	Staphylinidae	*Bolitochara lucida*	2		2	
23-.148-.003-.	Staphylinidae	*Autalia rivularis*		1		1
23-.152-.001-.	Staphylinidae	*Bohemiellina flavipennis*		1		1
23-.158-.002-.	Staphylinidae	*Callicerus rigidicornis*		2		2
23-.160-.004-.	Staphylinidae	*Schistoglossa curtipennis*	1	6	1	5
23-.166-.014-.	Staphylinidae	*Aloconota gregaria*		2		2
23-.1661.001-.	Staphylinidae	*Enalodroma hepatica*	43	50	27	25
23-.168-.001-.	Staphylinidae	*Amischa analis*	20	80	14	33
23-.168-.004-.	Staphylinidae	*Amischa nigrofusca*	1	10	1	7
23-.168-.007-.	Staphylinidae	*Amischa decipiens*		2		2
23-.168-.008-.	Staphylinidae	*Amischa forcipata*		1		1
23-.180-.003-.	Staphylinidae	*Geostiba circellaris*	5	2	4	1
23-.182-.002-.	Staphylinidae	*Dinaraea aequata*		10		5
23-.182-.003-.	Staphylinidae	*Dinaraea linearis*		3		2
23-.182-.004-.	Staphylinidae	*Dinaraea arcana*		1		1
23-.184-.001-.	Staphylinidae	*Dadobia immersa*	2		2	
23-.186-.004-.	Staphylinidae	*Plataraea dubiosa*	15	4	12	3
23-.187-.002-.	Staphylinidae	*Liogluta granigera*	2	2	2	1
23-.187-.004-.	Staphylinidae	*Liogluta longiuscula*	14	14	14	9
23-.187-.006-.	Staphylinidae	*Liogluta microptera*	3		3	
23-.187-.009-.	Staphylinidae	*Liogluta alpestris*	22	9	11	4
23-.188-.004-.	Staphylinidae	*Atheta elongatula*	2	1	2	1
23-.188-.020-.	Staphylinidae	*Atheta palustris*		3		3
23-.188-.063-.	Staphylinidae	*Atheta palleola*	1	1	1	1
23-.188-.104-.	Staphylinidae	*Atheta voeslauensis*		1		1
23-.188-.109-.	Staphylinidae	*Atheta sodalis*	7	9	4	7
23-.188-.110-.	Staphylinidae	*Atheta gagatina*	106	56	22	21
23-.188-.111-.	Staphylinidae	*Atheta pallidicornis*	1	1	1	1
23-.188-.136-.	Staphylinidae	*Atheta fungi*	68	110	37	46
23-.188-.1361.	Staphylinidae	*Atheta negligens*		1		1
23-.188-.161-.	Staphylinidae	*Atheta hypnorum*		2		2
23-.188-.168-.	Staphylinidae	*Atheta triangulum*	1		1	
23-.188-.176-.	Staphylinidae	*Atheta incognita*		1		1
23-.188-.179-.	Staphylinidae	*Atheta laticollis*	3		3	
23-.188-.181-.	Staphylinidae	*Atheta coriaria*		1		1
23-.188-.186-.	Staphylinidae	*Atheta myrmecobia*		3		2
23-.188-.198-.	Staphylinidae	*Atheta britanniae*	234	115	61	43
23-.188-.200-.	Staphylinidae	*Atheta paracrassicornis*		1		1
23-.188-.214-.	Staphylinidae	*Atheta europaea*	8		3	
23-.188-.215-.	Staphylinidae	*Atheta cinnamoptera*	1		1	
23-.188-.223-.	Staphylinidae	*Atheta longicornis*		1		1
23-.1881.002-.	Staphylinidae	*Acrotona sylvicola*	3		1	
23-.1881.0061.	Staphylinidae	*Acrotona pseudotenera*		1		1
23-.1881.013-.	Staphylinidae	*Acrotona parvula*	1	1	1	1

23-.190-.001-.	Staphylinidae	*Alevonota rufotestacea*	7	10	7	9
23-.190-.003-.	Staphylinidae	*Alevonota egregia*		3		2
23-.195-.001-.	Staphylinidae	*Drusilla canaliculata*	2	101	2	22
23-.196-.003-.	Staphylinidae	*Zyras haworthi*	1	1	1	1
23-.1960.006-.	Staphylinidae	*Pella funesta*		7		2
23-.1960.007-.	Staphylinidae	*Pella humeralis*		6		5
23-.1960.009-.	Staphylinidae	*Pella cognata*		1		1
23-.1960.010-.	Staphylinidae	*Pella lugens*	1	2	1	2
23-.198-.001-.	Staphylinidae	*Lomechusa emarginata*	1		1	
23-.201-.001-.	Staphylinidae	*Phloeopora corticalis*	12	5	11	5
23-.201-.004-.	Staphylinidae	*Phloeopora testacea*	2	1	1	1
23-.203-.002-.	Staphylinidae	*Ilyobates bennetti*	3		3	
23-.203-.003-.	Staphylinidae	*Ilyobates nigricollis*		3		3
23-.208-.002-.	Staphylinidae	*Amarochara bonnairei*	2	1	2	1
23-.210-.002-.	Staphylinidae	*Ocalea picata*	4	15	4	11
23-.213-.026-.	Staphylinidae	*Meotica filiformis*		1		1
23-.219-.001-.	Staphylinidae	*Mniusa incrassata*		1		1
23-.223-.004-.	Staphylinidae	*Oxypoda opaca*		2		2
23-.223-.009-.	Staphylinidae	*Oxypoda acuminata*	54	115	17	21
23-.223-.010-.	Staphylinidae	*Oxypoda spectabilis*	1		1	
23-.223-.018-.	Staphylinidae	*Oxypoda brevicornis*	8	10	7	7
23-.223-.034-.	Staphylinidae	*Oxypoda alternans*	32	16	18	12
23-.223-.041-.	Staphylinidae	*Oxypoda mutata*	4	1	3	1
23-.223-.046-.	Staphylinidae	*Oxypoda brachyptera*	1	1	1	1
23-.223-.047-.	Staphylinidae	*Oxypoda tarda*		2		2
23-.223-.049-.	Staphylinidae	*Oxypoda annularis*	3		3	
23-.223-.050-.	Staphylinidae	*Oxypoda flavicornis*		1		1
23-.227-.001-.	Staphylinidae	*Stichoglossa semirufa*	2	1	2	1
23-.228-.001-.	Staphylinidae	*Ischnoglossa prolixa*	1		1	
23-.229-.001-.	Staphylinidae	*Dexiogya corticina*		1		1
23-.230-.001-.	Staphylinidae	*Homoeusa acuminata*	2	4	2	4
23-.234-.002-.	Staphylinidae	*Haploglossa villosula*	18	20	15	15
23-.237-.001-.	Staphylinidae	*Aleochara curtula*	5	2	4	2
23-.237-.005-.	Staphylinidae	*Aleochara spissicornis*		1		1
23-.237-.008-.	Staphylinidae	*Aleochara brevipennis*		1		1
23-.237-.012-.	Staphylinidae	*Aleochara tristis*		1		1
23-.237-.015-.	Staphylinidae	*Aleochara sparsa*	10		7	
23-.237-.038-.	Staphylinidae	*Aleochara ruficornis*	1		1	
23-.237-.043-.	Staphylinidae	*Aleochara bilineata*		2		2
23-.237-.046-.	Staphylinidae	*Aleochara bipustulata*	1	5	1	5
24-.002-.002-.	Staphylinidae	*Bibloporus bicolor*	34	57	22	39
24-.002-.003-.	Staphylinidae	*Bibloporus minutus*	1	2	1	2
24-.005-.001-.	Staphylinidae	*Bibloplectus tenebrosus*		1		1
24-.006-.001-.	Staphylinidae	*Euplectus nanus*	1	1	1	1
24-.006-.003-.	Staphylinidae	*Euplectus piceus*		1		1
24-.006-.005-.	Staphylinidae	*Euplectus sparsus*		1		1
24-.006-.007-.	Staphylinidae	*Euplectus bescidicus*		4		4
24-.006-.013-.	Staphylinidae	*Euplectus punctatus*	2	3	2	3
24-.006-.015-.	Staphylinidae	*Euplectus karsteni*	2		2	
24-.006-.017-.	Staphylinidae	*Euplectus brunneus*	2	3	2	3

24-.008-.004-.	Staphylinidae	*Plectophloeus erichsoni*		3		3
24-.008-.005-.	Staphylinidae	*Plectophloeus nubigena*	1	1	1	1
24-.008-.009-.	Staphylinidae	*Plectophloeus fischeri*	13	29	10	22
24-.011-.001-.	Staphylinidae	*Trimium brevicorne*	2	2	2	2
24-.012-.001-.	Staphylinidae	*Trichonyx sulcicollis*		3		3
24-.015-.002-.	Staphylinidae	*Batrisodes venustus*	1	1	1	1
24-.017-.001-.	Staphylinidae	*Bythinus macropalpus*	1	3	1	3
24-.017-.002-.	Staphylinidae	*Bythinus burrellii*	7	5	7	5
24-.018-.002-.	Staphylinidae	*Bryaxis nodicornis*		15		12
24-.018-.003-.	Staphylinidae	*Bryaxis collaris*	11		11	
24-.018-.008-.	Staphylinidae	*Bryaxis puncticollis*	1	2	1	2
24-.018-.025-.	Staphylinidae	*Bryaxis ullrichii*	1		1	
24-.019-.001-.	Staphylinidae	*Tychus niger*	1	5	1	5
24-.021-.001-.	Staphylinidae	*Brachygluta fossulata*	1	14	1	12
24-.025-.002-.	Staphylinidae	*Pselaphus heisei*		2		2
25-.001-.001-.	Lycidae	*Dictyopterus aurora*	11	14	11	13
25-.002-.001-.	Lycidae	*Pyropterus nigroruber*	4		4	
25-.0041.001-.	Lycidae	*Erotides cosnardi*	1	2	1	2
25-.005-.001-.	Lycidae	*Lygistopterus sanguineus*		1		1
251.001-.001-.	Omalisidae	*Omalisus fontisbellaquaei*	6	36	6	18
26-.001-.001-.	Lampyridae	*Lampyris noctiluca*	4	6	4	6
26-.002-.001-.	Lampyridae	*Lamprohiza splendidula*	86	74	41	39
27-.001-.001-.	Cantharidae	*Podabrus alpinus*	35	50	16	27
27-.002-.005-.	Cantharidae	*Cantharis fusca*		2		2
27-.002-.007-.	Cantharidae	*Cantharis rustica*		1		1
27-.002-.008-.	Cantharidae	*Cantharis pellucida*	14	41	13	30
27-.002-.010-.	Cantharidae	*Cantharis nigra*	1	2	1	2
27-.002-.014-.	Cantharidae	*Cantharis obscura*	1	2	1	2
27-.002-.016-.	Cantharidae	*Cantharis paradoxa*	1	3	1	3
27-.002-.018-.	Cantharidae	*Cantharis nigricans*	1	5	1	3
27-.002-.019-.	Cantharidae	*Cantharis pagana*		8		5
27-.002-.021-.	Cantharidae	*Cantharis terminata*		1		1
27-.002-.025-.	Cantharidae	*Cantharis decipiens*	6	3	6	3
27-.002-.026-.	Cantharidae	*Cantharis livida*		1		1
27-.0021.001-.	Cantharidae	*Ancistronycha abdominalis*		1		1
27-.0022.001-.	Cantharidae	*Metacantharis discoidea*	3	1	3	1
27-.003-.004-.	Cantharidae	*Podistra prolixa*	5	1	4	1
27-.003-.005-.	Cantharidae	*Podistra rufotestacea*	11	24	10	15
27-.003-.006-.	Cantharidae	*Podistra schoenherri*	5	6	4	4
27-.005-.001-.	Cantharidae	*Rhagonycha lutea*	104	118	37	38
27-.005-.002-.	Cantharidae	*Rhagonycha fulva*	12	99	10	33
27-.005-.003-.	Cantharidae	*Rhagonycha translucida*	47	27	25	17
27-.005-.008-.	Cantharidae	*Rhagonycha lignosa*	41	160	35	51
27-.005-.014-.	Cantharidae	*Rhagonycha gallica*		6		5
27-.006-.001-.	Cantharidae	*Cratosilis denticollis*		3		3
27-.008-.001-.	Cantharidae	*Malthinus flaveolus*	46	58	31	33
27-.008-.002-.	Cantharidae	*Malthinus seriepunctatus*		6		6
27-.008-.005-.	Cantharidae	*Malthinus facialis*	12	9	6	8
27-.008-.006-.	Cantharidae	*Malthinus glabellus*	1		1	

27-.008-.009-.	Cantharidae	*Malthinus biguttatus*	1	2	1	2
27-.008-.010-.	Cantharidae	*Malthinus frontalis*		2		2
27-.009-.011-.	Cantharidae	*Malthodes fuscus*	4	6	4	6
27-.009-.012-.	Cantharidae	*Malthodes minimus*	8	3	8	3
27-.009-.014-.	Cantharidae	*Malthodes alpicola*	8		6	
27-.009-.015-.	Cantharidae	*Malthodes guttifer*		1		1
27-.009-.016-.	Cantharidae	*Malthodes marginatus*		1		1
27-.009-.017-.	Cantharidae	*Malthodes mysticus*	1	2	1	2
27-.009-.021-.	Cantharidae	*Malthodes hexacanthus*	2	3	2	3
27-.009-.022-.	Cantharidae	*Malthodes pumilus*	50	75	36	42
27-.009-.032-.	Cantharidae	*Malthodes brevicollis*	4	9	3	7
28-.001-.001-.	Drilidae	*Drilus concolor*	13	18	10	15
29-.006-.0032.	Malachiidae	*Malachius bipustulatus*	6	16	6	14
29-.007-.002-.	Malachiidae	*Anthocomus fasciatus*		1		1
30-.002-.001-.	Dasytidae	*Aplocnemus impressus*	27	17	15	7
30-.002-.002-.	Dasytidae	*Aplocnemus nigricornis*	1	2	1	2
30-.005-.001-.	Dasytidae	*Dasytes niger*	1	3	1	3
30-.005-.003-.	Dasytidae	*Dasytes obscurus*		3		1
30-.005-.005-.	Dasytidae	*Dasytes caeruleus*	112	77	52	44
30-.005-.008-.	Dasytidae	*Dasytes plumbeus*	15	116	10	41
30-.005-.009-.	Dasytidae	*Dasytes aeratus*	8	10	6	7
31-.002-.001-.	Cleridae	*Tillus elongatus*	20	20	16	16
31-.003-.001-.	Cleridae	*Tilloidea unifasciata*		2		2
31-.006-.002-.	Cleridae	*Opilo mollis*	4	4	4	4
31-.007-.001-.	Cleridae	*Thanasimus formicarius*	17	30	13	24
31-.007-.002-.	Cleridae	*Thanasimus femoralis*	1		1	
31-.009-.003-.	Cleridae	*Trichodes alvearius*		1		1
321.001-.001-.	Trogossitidae	*Nemozoma elongatum*	21	25	17	16
321.005-.002-.	Trogossitidae	*Peltis ferruginea*	21	13	14	11
321.006-.001-.	Trogossitidae	*Thymalus limbatus*	2	6	2	5
33-.001-.001-.	Lymexylidae	*Elateroides dermestoides*	54	156	30	40
34-.001-.004-.	Elateridae	*Ampedus erythrogonus*	7	13	7	11
34-.001-.008-.	Elateridae	*Ampedus balteatus*	245	48	10	9
34-.001-.012-.	Elateridae	*Ampedus aethiops*	3	26	2	11
34-.001-.014-.	Elateridae	*Ampedus nigerrimus*	1	2	1	2
34-.001-.015-.	Elateridae	*Ampedus sanguineus*	7	4	5	4
34-.001-.019-.	Elateridae	*Ampedus pomorum*	211	314	62	70
34-.001-.0201.	Elateridae	*Ampedus quercicola*	85	123	31	35
34-.001-.022-.	Elateridae	*Ampedus elongatulus*		2		1
34-.001-.026-.	Elateridae	*Ampedus nigrinus*	30	51	20	30
34-.001-.0261.	Elateridae	*Ampedus auripes*	1		1	
34-.0011.001-.	Elateridae	*Brachygonus megerlei*	1		1	
34-.008-.001-.	Elateridae	*Sericus brunneus*	3	27	2	10
34-.008-.002-.	Elateridae	*Sericus subaeneus*		3		1
34-.009-.001-.	Elateridae	*Dalopius marginatus*	511	624	81	82
34-.010-.002-.	Elateridae	*Agriotes pallidulus*	122	50	38	27
34-.010-.003-.	Elateridae	*Agriotes acuminatus*	90	60	39	21

34-.010-.004-.	Elateridae	*Agriotes gallicus*		6		6
34-.010-.007-.	Elateridae	*Agriotes pilosellus*	371	317	57	49
34-.010-.009-.	Elateridae	*Agriotes lineatus*		1		1
34-.010-.014-.	Elateridae	*Agriotes sputator*	1	2	1	2
34-.0101.001-.	Elateridae	*Ectinus aterrimus*	2	82	2	15
34-.015-.004-.	Elateridae	*Adrastus pallens*		2		1
34-.016-.002-.	Elateridae	*Melanotus villosus*	19	228	2	63
34-.016-.003-.	Elateridae	*Melanotus castanipes*	808	619	112	92
34-.019-.001-.	Elateridae	*Agrypnus murinus*	3	8	3	6
34-.021-.001-.	Elateridae	*Orithales serraticornis*		10		7
34-.022-.004-.	Elateridae	*Ctenicera cuprea*	1	41	1	12
34-.023-.001-.	Elateridae	*Liotrichus affinis*	2		2	
34-.025-.001-.	Elateridae	*Prosternon tessellatum*	2	1	2	1
34-.026-.001-.	Elateridae	*Anostirus purpureus*	5	36	3	27
34-.026-.003-.	Elateridae	*Anostirus castaneus*		2		2
34-.026-.005-.	Elateridae	*Anostirus sulphuripennis*		1		1
34-.030-.001-.	Elateridae	*Calambus bipustulatus*	4	6	4	5
34-.031-.001-.	Elateridae	*Hypoganus inunctus*	20	11	17	6
34-.033-.002-.	Elateridae	*Denticollis rubens*	7	4	6	4
34-.033-.004-.	Elateridae	*Denticollis linearis*	87	92	44	44
34-.034-.001-.	Elateridae	*Cidnopus pilosus*		3		3
34-.0342.001-.	Elateridae	*Nothodes parvulus*	236	225	34	36
34-.0343.001-.	Elateridae	*Pheletes aeneoniger*	22	48	8	11
34-.035-.002-.	Elateridae	*Limonius minutus*		1		1
34-.035-.003-.	Elateridae	*Limonius poneli*		105		21
34-.0371.001-.	Elateridae	*Diacanthous undulatus*		1		1
34-.038-.002-.	Elateridae	*Stenagostus rhombeus*	6	12	6	11
34-.039-.001-.	Elateridae	*Hemicrepidius niger*	13	4	9	3
34-.039-.002-.	Elateridae	*Hemicrepidius hirtus*		8		7
34-.041-.001-.	Elateridae	*Athous haemorrhoidalis*	450	297	65	60
34-.041-.002-.	Elateridae	*Athous vittatus*	823	899	87	78
34-.041-.003-.	Elateridae	*Athous subfuscus*	2671	2525	90	78
34-.041-.004-.	Elateridae	*Athous zebei*	1316	1526	46	44
34-.041-.011-.	Elateridae	*Athous bicolor*	9	11	6	10
34-.044-.002-.	Elateridae	*Oedostethus quadripustulatus*		1		1
34-.049-.001-.	Elateridae	*Cardiophorus nigerrimus*		1		1
34-.050-.001-.	Elateridae	*Dicronychus cinereus*		1		1
35-.001-.001-.	Cerophytidae	*Cerophytum elaterolides*		1		1
36-.001-.001-.	Eucnemidae	*Melasis buprestoides*	45	30	24	23
36-.002-.001-.	Eucnemidae	*Isorhipis melasoides*	3	2	3	1
36-.002-.002-.	Eucnemidae	*Isorhipis marmottani*	3	15	3	11
36-.003-.001-.	Eucnemidae	*Eucnemis capucina*		1		1
36-.004-.001-.	Eucnemidae	*Dromaeolus barnabita*		2		1
36-.0082.003-.	Eucnemidae	*Microrhagus pygmaeus*	15	6	11	6
36-.0082.004-.	Eucnemidae	*Microrhagus lepidus*	2	3	2	2
36-.0101.001-.	Eucnemidae	*Epiphanis cornutus*	1	1	1	1

36-.011-.001-.	Eucnemidae	*Hylis olexai*	4	20	4	9
36-.011-.002-.	Eucnemidae	*Hylis cariniceps*	5	9	5	8
36-.011-.003-.	Eucnemidae	*Hylis foveicollis*	7	6	7	6
36-.011-.004-.	Eucnemidae	*Hylis procerulus*	3	2	3	2
36-.012-.001-.	Eucnemidae	*Xylophilus corticalis*	1	9	1	6
37-.001-.002-.	Throscidae	*Trixagus dermestoides*	213	137	59	60
37-.001-.003-.	Throscidae	*Trixagus carinifrons*	10		2	
37-.001-.0031.	Throscidae	*Trixagus gracilis*		1		1
37-.001-.0032.	Throscidae	*Trixagus leseigneuri*	4	1	1	1
37-.001-.0033.	Throscidae	*Trixagus meybohmi*	273	208	60	64
37-.001-.006-.	Throscidae	*Trixagus obtusus*	5	4	5	3
37-.002-.001-.	Throscidae	*Aulonothroscus brevicollis*	4	52	4	21
38-.015-.015-.	Buprestidae	*Anthaxia nitidula*		4		3
38-.015-.019-.	Buprestidae	*Anthaxia helvetica*		5		3
38-.015-.023-.	Buprestidae	*Anthaxia quadripunctata*		4		3
38-.020-.003-.	Buprestidae	*Agrilus biguttatus*		1		1
38-.020-.004-.	Buprestidae	*Agrilus laticornis*	3		3	
38-.020-.006-.	Buprestidae	*Agrilus angustulus*	1	1	1	1
381.001-.001-.	Clambidae	*Calyptomerus alpestris*		1		1
381.002-.001-.	Clambidae	*Clambus pubescens*		2		2
381.002-.0011.	Clambidae	*Clambus simsoni*		1		1
381.002-.004-.	Clambidae	*Clambus pallidulus*	6	3	6	3
381.002-.007-.	Clambidae	*Clambus armadillo*		3		3
39-.001-.001-.	Dascillidae	*Dascillus cervinus*	1	3	1	3
40-.001-.001-.	Scirtidae	*Elodes minuta*	1	5	1	4
40-.003-.001-.	Scirtidae	*Contacyphon coarctatus*	3	12	3	7
40-.003-.002-.	Scirtidae	*Contacyphon palustris*		1		1
40-.003-.003-.	Scirtidae	*Contacyphon ruficeps*	1		1	
40-.003-.011-.	Scirtidae	*Contacyphon padi*		5		5
40-.004-.001-.	Scirtidae	*Prionocyphon serricornis*	5	2	5	2
41-.001-.001-.	Eucinetidae	*Eucinetus haemorrhoidalis*		1		1
42-.002-.002-.	Dryopidae	*Dryops ernesti*	1	2	1	2
421.003-.003-.	Elmidae	*Elmis maugetii*		1		1
45-.001-.004-.	Dermestidae	*Dermestes murinus*	2		2	
45-.006-.001-.	Dermestidae	*Megatoma undata*	1	2	1	2
45-.008-.010-.	Dermestidae	*Anthrenus museorum*	1		1	
47-.010-.001-.	Byrrhidae	*Cytilus sericeus*		3		3
47-.011-.002-.	Byrrhidae	*Byrrhus pilula*	1	5	1	4
47-.011-.003-.	Byrrhidae	*Byrrhus arietinus*		6		6
47-.011-.006-.	Byrrhidae	*Byrrhus luniger*	6	7	3	7
47-.011-.007-.	Byrrhidae	*Byrrhus glabratus*	6	10	6	8
47-.012-.001-.	Byrrhidae	*Porcinolus murinus*	4	6	4	5
49-.001-.001-.	Byturidae	*Byturus tomentosus*	3	37	3	16
49-.001-.002-.	Byturidae	*Byturus ochraceus*	12	37	7	17
492.002-.001-.	Cerylonidae	*Cerylon fagi*	33	35	27	25

492.002-.002-.	Cerylonidae	*Cerylon histeroides*	47	98	32	52
492.002-.003-.	Cerylonidae	*Cerylon ferrugineum*	38	64	27	42
493.001-.008-.	Alexiidae	*Sphaerosoma piliferum*		2		2
50-.008-.003-.	Nitidulidae	*Meligethes denticulatus*	7	76	6	20
50-.008-.014-.	Nitidulidae	*Meligethes aeneus*	58	207	37	63
50-.008-.030-.	Nitidulidae	*Meligethes brunnicornis*		1		1
50-.008-.033-.	Nitidulidae	*Meligethes pedicularius*		4		3
50-.008-.058-.	Nitidulidae	*Meligethes nigrescens*	6	3	6	3
50-.009-.001-.	Nitidulidae	*Epuraea melanocephala*	283	217	47	50
50-.009-.002-.	Nitidulidae	*Epuraea guttata*		2		2
50-.009-.005-.	Nitidulidae	*Epuraea neglecta*		2		2
50-.009-.009-.	Nitidulidae	*Epuraea deubeli*		1		1
50-.009-.010-.	Nitidulidae	*Epuraea thoracica*	1		1	
50-.009-.011-.	Nitidulidae	*Epuraea angustula*		1		1
50-.009-.012-.	Nitidulidae	*Epuraea oblonga*	2		2	
50-.009-.015-.	Nitidulidae	*Epuraea marseuli*	16	11	12	9
50-.009-.016-.	Nitidulidae	*Epuraea pygmaea*	20	8	16	7
50-.009-.017-.	Nitidulidae	*Epuraea longula*	2	1	2	1
50-.009-.018-.	Nitidulidae	*Epuraea binotata*	9	14	8	11
50-.009-.027-.	Nitidulidae	*Epuraea unicolor*	22	29	19	14
50-.009-.028-.	Nitidulidae	*Epuraea variegata*		1		1
50-.009-.030-.	Nitidulidae	*Epuraea muehli*		1		1
50-.009-.033-.	Nitidulidae	*Epuraea aestiva*	27	15	19	12
50-.009-.035-.	Nitidulidae	*Epuraea rufomarginata*		1		1
50-.012-.001-.	Nitidulidae	*Amphotis marginata*	2	1	2	1
50-.013-.001-.	Nitidulidae	*Soronia punctatissima*	1		1	
50-.013-.002-.	Nitidulidae	*Soronia grisea*	2	2	2	2
50-.0131.001-.	Nitidulidae	*Stelidota geminata*	1	2	1	1
50-.014-.001-.	Nitidulidae	*Ipidia binotata*	2	4	2	4
50-.015-.001-.	Nitidulidae	*Pocadius ferrugineus*	1		1	
50-.017-.001-.	Nitidulidae	*Thalycra fervida*	2	2	2	2
50-.019-.001-.	Nitidulidae	*Cychramus variegatus*	2	10	2	7
50-.019-.002-.	Nitidulidae	*Cychramus luteus*	9	13	8	10
50-.020-.001-.	Nitidulidae	*Cryptarcha strigata*		5		2
50-.021-.001-.	Nitidulidae	*Glischrochilus quadriguttatus*	2	2	2	2
50-.021-.002-.	Nitidulidae	*Glischrochilus hortensis*		1		1
50-.021-.0021.	Nitidulidae	*Glischrochilus quadrisignatus*	2	3	2	2
50-.021-.003-.	Nitidulidae	*Glischrochilus quadripunctatus*	6	4	5	4
50-.022-.001-.	Nitidulidae	*Pityophagus ferrugineus*	10	12	10	10
501.001-.003-.	Kateretidae	*Kateretes rufilabris*		1		1
501.002-.001-.	Kateretidae	*Heterhelus scutellaris*		1		1
501.003-.001-.	Kateretidae	*Brachypterus urticae*		1		1

52-.0001.006-.	Monotomidae	*Monotoma brevicollis*		2		2
52-.0001.009-.	Monotomidae	*Monotoma longicollis*		2		2
52-.001-.002-.	Monotomidae	*Rhizophagus grandis*	2		2	
52-.001-.003-.	Monotomidae	*Rhizophagus depressus*	69	11	13	9
52-.001-.004-.	Monotomidae	*Rhizophagus ferrugineus*	3	1	3	1
52-.001-.006-.	Monotomidae	*Rhizophagus perforatus*	19	24	17	20
52-.001-.008-.	Monotomidae	*Rhizophagus dispar*	12	24	11	18
52-.001-.009-.	Monotomidae	*Rhizophagus bipustulatus*	68	57	41	37
52-.001-.010-.	Monotomidae	*Rhizophagus nitidulus*	17	35	13	18
52-.001-.012-.	Monotomidae	*Rhizophagus fenestralis*	4		4	
52-.001-.013-.	Monotomidae	*Rhizophagus cribratus*		17		12
53-.015-.002-.	Cucujidae	*Pediacus dermestoides*	4	3	4	2
531.006-.001-.	Silvanidae	*Silvanus bidentatus*	1	4	1	4
531.006-.002-.	Silvanidae	*Silvanus unidentatus*	1	1	1	1
531.007-.001-.	Silvanidae	*Silvanoprus fagi*	3	2	3	2
531.011-.001-.	Silvanidae	*Uleiota planatus*	1	22	1	15
54-.001-.001-.	Erotylidae	*Tritoma bipustulata*	8	58	8	30
54-.002-.001-.	Erotylidae	*Triplax aenea*	6	28	6	13
54-.002-.003-.	Erotylidae	*Triplax russica*	9	19	8	15
54-.002-.007-.	Erotylidae	*Triplax scutellaris*	1		1	
54-.002-.008-.	Erotylidae	*Triplax lepida*	1	5	1	5
54-.002-.009-.	Erotylidae	*Triplax rufipes*	14	39	11	18
54-.003-.004-.	Erotylidae	*Dacne bipustulata*	16	88	12	26
541.002-.001-.	Biphyllidae	*Diplocoelus fagi*		3		2
55-.007-.001-.	Cryptophagidae	*Pteryngium crenatum*	3	6	3	6
55-.008-.016-.	Cryptophagidae	*Cryptophagus lapponicus*		2		2
55-.008-.019-.	Cryptophagidae	*Cryptophagus pubescens*	4	4	4	4
55-.008-.027-.	Cryptophagidae	*Cryptophagus dentatus*	69	81	43	37
55-.008-.029-.	Cryptophagidae	*Cryptophagus dorsalis*	4		3	
55-.008-.030-.	Cryptophagidae	*Cryptophagus distinguendus*		2		2
55-.008-.035-.	Cryptophagidae	*Cryptophagus reflexus*	8	2	6	2
55-.008-.040-.	Cryptophagidae	*Cryptophagus lycoperdi*	31	346	11	19
55-.008-.053-.	Cryptophagidae	*Cryptophagus montanus*	15	9	14	9
55-.0081.005-.	Cryptophagidae	*Micrambe abietis*	6	16	6	13
55-.011-.001-.	Cryptophagidae	*Antherophagus pallens*		7		6
55-.011-.002-.	Cryptophagidae	*Antherophagus silaceus*	2	1	2	1
55-.012-.003-.	Cryptophagidae	*Caenoscelis sibirica*		1		1
55-.014-.006-.	Cryptophagidae	*Atomaria ornata*	7	10	7	8
55-.014-.014-.	Cryptophagidae	*Atomaria fuscata*	8	27	7	12
55-.014-.016-.	Cryptophagidae	*Atomaria lewisi*		1		1
55-.014-.025-.	Cryptophagidae	*Atomaria atricapilla*		1		1

55-.014-.028-.	Cryptophagidae	*Atomaria analis*	3	11	3	9
55-.014-.033-.	Cryptophagidae	*Atomaria turgida*	31	61	27	30
55-.014-.034-.	Cryptophagidae	*Atomaria apicalis*		1		1
55-.014-.037-.	Cryptophagidae	*Atomaria fimetarii*	1		1	
55-.014-.038-.	Cryptophagidae	*Atomaria umbrina*		1		1
55-.014-.041-.	Cryptophagidae	*Atomaria diluta*	2	16	2	13
55-.014-.045-.	Cryptophagidae	*Atomaria nigrirostris*		5		4
55-.014-.046-.	Cryptophagidae	*Atomaria linearis*	1	8	1	8
55-.014-.049-.	Cryptophagidae	*Atomaria abietina*		2		2
55-.014-.051-.	Cryptophagidae	*Atomaria pulchra*	6	4	5	4
55-.014-.053-.	Cryptophagidae	*Atomaria longicornis*	1		1	
55-.014-.054-.	Cryptophagidae	*Atomaria bella*	1		1	
55-.016-.001-.	Cryptophagidae	*Ephistemus globulus*	1	4	1	4
56-.002-.007-.	Phalacridae	*Olibrus bisignatus*	1		1	
56-.002-.010-.	Phalacridae	*Olibrus liquidus*	2		2	
561.001-.001-.	Laemophloeidae	*Laemophloeus monilis*	1	4	1	3
561.001-.002-.	Laemophloeidae	*Laemophloeus kraussi*	1		1	
561.002-.001-.	Laemophloeidae	*Placonotus testaceus*	2	3	2	2
561.004-.001-.	Laemophloeidae	*Cryptolestes duplicatus*		1		1
561.004-.005-.	Laemophloeidae	*Cryptolestes ferrugineus*		1		1
561.004-.007-.	Laemophloeidae	*Cryptolestes corticinus*	1		1	
561.005-.003-.	Laemophloeidae	*Leptophloeus alternans*	3	2	3	1
58-.003-.0021.	Latridiidae	*Latridius minutus*		12		11
58-.003-.0031.	Latridiidae	*Latridius assimilis*		1		1
58-.003-.0081.	Latridiidae	*Latridius hirtus*	8	12	8	9
58-.003-.0101.	Latridiidae	*Latridius consimilis*	1		1	
58-.004-.009-.	Latridiidae	*Enicmus brevicornis*	3	9	3	7
58-.004-.010-.	Latridiidae	*Enicmus fungicola*	3	9	2	6
58-.004-.012-.	Latridiidae	*Enicmus rugosus*	92	192	45	66
58-.004-.013-.	Latridiidae	*Enicmus testaceus*	15	32	11	23
58-.004-.014-.	Latridiidae	*Enicmus transversus*	5	1	5	1
58-.004-.015-.	Latridiidae	*Enicmus histrio*	2	1	1	1
58-.0041.001-.	Latridiidae	*Dienerella vincenti*	9	1	7	1
58-.0041.0021.	Latridiidae	*Dienerella clathrata*		1		1
58-.005-.0011.	Latridiidae	*Cartodere constricta*		1		1
58-.005-.0031.	Latridiidae	*Cartodere nodifer*	50	80	37	51
58-.0061.002-.	Latridiidae	*Stephostethus angusticollis*	53	104	20	36
58-.0061.006-.	Latridiidae	*Stephostethus alternans*	62	161	38	66
58-.0061.007-.	Latridiidae	*Stephostethus rugicollis*	4	8	4	6
58-.007-.007-.	Latridiidae	*Corticaria longicornis*	7	7	7	6
58-.007-.010-.	Latridiidae	*Corticaria saginata*	1		1	

58-.007-.0131.	Latridiidae	*Corticaria interstitialis*		1		1
58-.007-.0171.	Latridiidae	*Corticaria lateritia*		1		1
58-.007-.021-.	Latridiidae	*Corticaria elongata*		1		1
58-.008-.0011.	Latridiidae	*Corticarina alemannica*	2		1	
58-.008-.002-.	Latridiidae	*Corticarina similata*	13	36	9	22
58-.008-.0021.	Latridiidae	*Corticarina lambiana*	10	1	8	1
58-.008-.003-.	Latridiidae	*Corticarina parvula*		74		37
58-.008-.005-.	Latridiidae	*Corticarina minuta*	2	3	2	3
58-.0081.001-.	Latridiidae	*Cortinicara gibbosa*	449	1620	78	75
58-.009-.002-.	Latridiidae	*Melanophthalma distinguenda*	2	4	2	1
59-.002-.001-.	Mycetophagidae	*Triphyllus bicolor*	1	2	1	2
59-.003-.001-.	Mycetophagidae	*Litargus connexus*	18	21	13	10
59-.004-.001-.	Mycetophagidae	*Mycetophagus quadripustulatus*	5	11	5	7
59-.004-.003-.	Mycetophagidae	*Mycetophagus piceus*	1		1	
59-.004-.004-.	Mycetophagidae	*Mycetophagus salicis*	2		2	
59-.004-.006-.	Mycetophagidae	*Mycetophagus atomarius*	9	19	9	18
59-.004-.007-.	Mycetophagidae	*Mycetophagus quadriguttatus*	1	1	1	1
59-.004-.009-.	Mycetophagidae	*Mycetophagus fulvicollis*	1		1	
59-.004-.010-.	Mycetophagidae	*Mycetophagus populi*	1	2	1	2
59-.006-.001-.	Mycetophagidae	*Berginus tamarisci*		1		1
60-.003-.001-.	Zopheridae	*Pycnomerus terebrans*	3		3	
60-.013-.001-.	Zopheridae	*Synchita humeralis*	2	1	2	1
60-.013-.004-.	Zopheridae	*Synchita variegata*	3	3	3	3
60-.013-.005-.	Zopheridae	*Synchita undata*	4	17	4	12
60-.016-.001-.	Zopheridae	*Bitoma crenata*	3	14	3	9
60-.018-.001-.	Zopheridae	*Colydium elongatum*	2	2	2	2
601.002-.001-.	Corylophidae	*Arthrolips obscura*	8	9	7	5
601.004-.001-.	Corylophidae	*Sericoderus lateralis*	27	20	22	15
601.006-.001-.	Corylophidae	*Corylophus cassidoides*		1		1
601.008-.003-.	Corylophidae	*Orthoperus atomus*	18	29	16	23
601.008-.004-.	Corylophidae	*Orthoperus corticalis*		7		6
61-.010-.001-.	Endomychidae	*Lycoperdina bovistae*	1		1	
61-.012-.001-.	Endomychidae	*Mycetina cruciata*	10	13	9	12
61-.013-.001-.	Endomychidae	*Endomychus coccineus*	3	5	3	5
62-.008-.012-.	Coccinellidae	*Scymnus auritus*	1		1	
62-.008-.015-.	Coccinellidae	*Scymnus suturalis*	2		2	
62-.0081.004-.	Coccinellidae	*Nephus bipunctatus*	1		1	
62-.010-.001-.	Coccinellidae	*Clitostethus arcuatus*		1		1
62-.012-.002-.	Coccinellidae	*Chilocorus renipustulatus*	1		1	
62-.013-.001-.	Coccinellidae	*Exochomus quadripustulatus*		4		4
62-.017-.001-.	Coccinellidae	*Aphidecta obliterata*	4	3	4	3
62-.018-.003-.	Coccinellidae	*Hippodamia variegata*	1		1	

62-.022-.001-.	Coccinellidae	*Tytthaspis sedecimpunctata*	1	6	1	6
62-.023-.002-.	Coccinellidae	*Adalia decempunctata*	4	7	4	7
62-.025-.003-.	Coccinellidae	*Coccinella septempunctata*	26	62	13	31
62-.025-.004-.	Coccinellidae	*Coccinella magnifica*	1		1	
62-.028-.002-.	Coccinellidae	*Harmonia axyridis*	8	13	8	11
62-.029-.001-.	Coccinellidae	*Myrrha octodecimguttata*		1		1
62-.031-.001-.	Coccinellidae	*Calvia decemguttata*	10	8	10	7
62-.032-.001-.	Coccinellidae	*Propylea quatuordecimpunctata*	11	15	11	12
62-.034-.001-.	Coccinellidae	*Anatis ocellata*	8	10	7	8
62-.035-.001-.	Coccinellidae	*Halyzia sedecimguttata*	8	6	6	4
62-.036-.001-.	Coccinellidae	*Vibidia duodecimguttata*		2		2
62-.037-.001-.	Coccinellidae	*Psyllobora vigintiduopunctata*		3		3
63-.001-.001-.	Sphindidae	*Sphindus dubius*		1		1
63-.002-.001-.	Sphindidae	*Aspidiphorus orbiculatus*	7	10	6	7
65-.001-.001-.	Ciidae	*Octotemnus glabriculus*	5	7	5	5
65-.003-.001-.	Ciidae	*Ropalodontus perforatus*	1	4	1	4
65-.005-.001-.	Ciidae	*Sulcacis nitidus*		13		11
65-.005-.003-.	Ciidae	*Sulcacis fronticornis*		4		3
65-.006-.001-.	Ciidae	*Cis lineatocribratus*	1	3	1	3
65-.006-.004-.	Ciidae	*Cis glabratus*	9	6	7	6
65-.006-.010-.	Ciidae	*Cis submicans*	28	56	23	36
65-.006-.011-.	Ciidae	*Cis boleti*	9	36	8	24
65-.006-.012-.	Ciidae	*Cis quadridens*	3	2	3	2
65-.006-.013-.	Ciidae	*Cis punctulatus*	2	1	2	1
65-.006-.015-.	Ciidae	*Cis fusciclavis*	4	3	4	3
65-.006-.016-.	Ciidae	*Cis dentatus*	17	10	14	7
65-.006-.027-.	Ciidae	*Cis vestitus*		1		1
65-.006-.028-.	Ciidae	*Cis festivus*	11	7	9	7
65-.0061.001-.	Ciidae	*Orthocis alni*	2	3	2	3
65-.007-.002-.	Ciidae	*Ennearthron cornutum*	6	4	6	4
65-.008-.001-.	Ciidae	*Hadraule elongatula*		3		2
67-.004-.001-.	Bostrichidae	*Rhyzopertha dominica*	1		1	
68-.0011.001-.	Ptinidae	*Ptinomorphus imperialis*	209	132	91	52
68-.003-.003-.	Ptinidae	*Dryophilus pusillus*	5	4	5	4
68-.005-.002-.	Ptinidae	*Xestobium rufovillosum*	5		5	
68-.005-.003-.	Ptinidae	*Xestobium austriacum*	1		1	
68-.0051.001-.	Ptinidae	*Hyperisus plumbeum*	19	17	16	13
68-.007-.003-.	Ptinidae	*Ernobius abietinus*		4		4
68-.007-.012-.	Ptinidae	*Ernobius mollis*	2	2	2	2
68-.010-.001-.	Ptinidae	*Gastrallus immarginatus*	1	6	1	6
68-.012-.001-.	Ptinidae	*Anobium punctatum*	1		1	
68-.0122.001-.	Ptinidae	*Hemicoelus canaliculatus*	5	7	4	2
68-.0122.002-.	Ptinidae	*Hemicoelus costatus*	184	73	58	35

68-.0122.003-.	Ptinidae	*Hemicoelus fulvicornis*	34	16	22	10
68-.0122.004-.	Ptinidae	*Hemicoelus rufipennis*	8	4	4	3
68-.0123.001-.	Ptinidae	*Microbregma emarginatum*	38	6	9	6
68-.0124.001-.	Ptinidae	*Hadrobregmus denticollis*	10	4	10	4
68-.0124.002-.	Ptinidae	*Hadrobregmus pertinax*	4	6	4	5
68-.014-.001-.	Ptinidae	*Ptilinus pectinicornis*	148	194	48	69
68-.016-.005-.	Ptinidae	*Xyletinus ater*		4		4
68-.022-.0041.	Ptinidae	*Dorcatoma lomnickii*	1	2	1	1
68-.022-.0042.	Ptinidae	*Dorcatoma minor*		1		1
68-.022-.005-.	Ptinidae	*Dorcatoma punctulata*	4	7	4	4
68-.022-.006-.	Ptinidae	*Dorcatoma dresdensis*		1		1
68-.022-.007-.	Ptinidae	*Dorcatoma robusta*		5		3
69-.008-.004-.	Ptinidae	*Ptinus rufipes*	2	1	2	1
69-.008-.005-.	Ptinidae	*Ptinus fur*	3	1	2	1
69-.008-.013-.	Ptinidae	*Ptinus subpilosus*	2	8	2	7
69-.008-.016-.	Ptinidae	*Ptinus dubius*		1		1
69-.008-.017-.	Ptinidae	*Ptinus sexpunctatus*		1		1
70-.001-.001-.	Oedemeridae	*Calopus serraticornis*	2		2	
70-.004-.0021.	Oedemeridae	*Nacerdes carniolica*	1		1	
70-.006-.001-.	Oedemeridae	*Chrysanthia viridissima*		1		1
70-.007-.001-.	Oedemeridae	*Ischnomera sanguinicollis*	1	5	1	5
70-.007-.002-.	Oedemeridae	*Ischnomera caerulea*	6	2	6	2
70-.007-.003-.	Oedemeridae	*Ischnomera cinerascens*	1	2	1	2
70-.010-.002-.	Oedemeridae	*Oedemera podagrariae*	1	3	1	2
70-.010-.005-.	Oedemeridae	*Oedemera femorata*		2		1
70-.010-.006-.	Oedemeridae	*Oedemera pthysica*		1		1
711.004-.001-.	Salpingidae	*Sphaeriestes castaneus*	2		2	
711.005-.001-.	Salpingidae	*Vincenzellus ruficollis*	510	475	54	48
711.006-.002-.	Salpingidae	*Salpingus planirostris*	15	11	13	10
711.006-.003-.	Salpingidae	*Salpingus ruficollis*	45	28	25	21
713.001-.001-.	Prostomidae	*Prostomis mandibularis*	14	1	5	1
72-.001-.001-.	Pyrochroidae	*Pyrochroa coccinea*		6		5
72-.001-.002-.	Pyrochroidae	*Pyrochroa serraticornis*	29	164	12	36
72-.002-.001-.	Pyrochroidae	*Schizotus pectinicornis*	12	24	7	19
73-.003-.001-.	Scraptiidae	*Cyrtanaspis phalerata*	1	1	1	1
73-.004-.009-.	Scraptiidae	*Anaspis frontalis*	12	263	9	27
73-.004-.010-.	Scraptiidae	*Anaspis maculata*		6		4
73-.004-.011-.	Scraptiidae	*Anaspis septentrionalis*		5		5
73-.004-.012-.	Scraptiidae	*Anaspis thoracica*	12	13	5	11
73-.004-.013-.	Scraptiidae	*Anaspis ruficollis*	18	16	14	13
73-.004-.019-.	Scraptiidae	*Anaspis rufilabris*	116	99	59	43

73-.004-.021-.	Scraptiidae	*Anaspis costai*	3	4	3	4
73-.004-.022-.	Scraptiidae	*Anaspis flava*	1	2	1	2
73-.004-.034-.	Scraptiidae	*Anaspis chevrolati*	13	40	11	27
74-.003-.001-.	Aderidae	*Euglenes pygmaeus*		1		1
74-.003-.002-.	Aderidae	*Euglenes oculatus*	3		3	
74-.004-.001-.	Aderidae	*Anidorus nigrinus*	1		1	
75-.001-.003-.	Anthicidae	*Notoxus monoceros*	1		1	
75-.004-.0071.	Anthicidae	*Anthicus antherinus*	2		2	
75-.0043.002-.	Anthicidae	*Omonadus floralis*		1		1
76-.007-.002-.	Meloidae	*Meloe violaceus*		1		1
77-.003-.001-.	Ripiphoridae	*Metoecus paradoxus*		1		1
79-.001-.001-.	Mordellidae	*Tomoxia bucephala*	18	105	10	40
79-.002-.001-.	Mordellidae	*Variimorda villosa*		16		6
79-.003-.007-.	Mordellidae	*Mordella brachyura*		8		6
79-.003-.008-.	Mordellidae	*Mordella holomelaena*		12		5
79-.006-.002-.	Mordellidae	*Curtimorda bisignata*		1		1
79-.011-.042-.	Mordellidae	*Mordellistena pygmaeola*		1		1
79-.011-.044-.	Mordellidae	*Mordellistena pumila*		2		1
79-.011-.052-.	Mordellidae	*Mordellistena neuwaldeggiana*	8	10	6	8
79-.011-.053-.	Mordellidae	*Mordellistena variegata*		7		4
79-.011-.054-.	Mordellidae	*Mordellistena humeralis*		1		1
79-.012-.001-.	Mordellidae	*Mordellochroa abdominalis*	28	70	19	30
80-.005-.002-.	Melandryidae	*Orchesia micans*	7	3	5	3
80-.005-.004-.	Melandryidae	*Orchesia minor*	1	1	1	1
80-.005-.005-.	Melandryidae	*Orchesia fasciata*	1		1	
80-.005-.006-.	Melandryidae	*Orchesia undulata*	32	81	24	42
80-.007-.001-.	Melandryidae	*Abdera affinis*		1		1
80-.007-.002-.	Melandryidae	*Abdera flexuosa*	7		4	
80-.009-.002-.	Melandryidae	*Phloiotrya rufipes*	4	4	4	4
80-.0111.002-.	Melandryidae	*Dolotarsus lividus*	1	1	1	1
80-.012-.001-.	Melandryidae	*Serropalpus barbatus*	7	3	7	3
80-.013-.001-.	Melandryidae	*Hypulus quercinus*	2	3	2	3
80-.016-.001-.	Melandryidae	*Melandrya caraboides*	3	34	2	25
80-.016-.002-.	Melandryidae	*Melandrya barbata*	3	6	3	5
80-.018-.001-.	Melandryidae	*Conopalpus testaceus*	2		2	
80-.018-.002-.	Melandryidae	*Conopalpus brevicollis*	2	5	2	4
80-.019-.001-.	Melandryidae	*Osphya bipunctata*	4	30	3	23
801.001-.003-.	Tetratomidae	*Tetratoma ancora*	10		9	
801.004-.001-.	Tetratomidae	*Hallomenus binotatus*	1	1	1	1
81-.001-.001-.	Tenebrionidae	*Lagria hirta*	11	27	8	21
81-.001-.002-.	Tenebrionidae	*Lagria atripes*	4	1	3	1
82-.001-.002-.	Tenebrionidae	*Allecula morio*	1		1	
82-.003-.002-.	Tenebrionidae	*Prionychus melanarius*	2	9	2	3
82-.005-.001-.	Tenebrionidae	*Pseudocistela ceramboides*	2	5	2	5
82-.006-.001-.	Tenebrionidae	*Gonodera luperus*	1	1	1	1
82-.008-.011-.	Tenebrionidae	*Mycetochara maura*	3	13	3	9
83-.014-.001-.	Tenebrionidae	*Bolitophagus reticulatus*	16	29	12	17

83-.018-.001-.	Tenebrionidae	*Neomida haemorrhoidalis*		1		1
83-.019-.001-.	Tenebrionidae	*Scaphidema metallica*	1	1	1	1
83-.020-.001-.	Tenebrionidae	*Platydema violacea*	1		1	
83-.023-.001-.	Tenebrionidae	*Corticeus unicolor*	7	26	7	18
83-.023-.008-.	Tenebrionidae	*Corticeus fasciatus*		1		1
83-.023-.009-.	Tenebrionidae	*Corticeus linearis*	1	1	1	1
83-.024-.002-.	Tenebrionidae	*Palorus depressus*		1		1
83-.030-.001-.	Tenebrionidae	*Uloma culinaris*		2		2
83-.030-.002-.	Tenebrionidae	*Uloma rufa*	2	1	2	1
83-.039-.001-.	Tenebrionidae	*Stenomax aeneus*	7	8	5	5
841.001-.002-.	Trogidae	*Trox sabulosus*		2		2
841.001-.004-.	Trogidae	*Trox scaber*	1	1	1	1
842.003-.001-.	Geotrupidae	*Typhaeus typhoeus*	1		1	
842.005-.001-.	Geotrupidae	*Anoplotrupes stercorosus*	9730	9103	114	100
842.006-.002-.	Geotrupidae	*Trypocopris vernalis*	1		1	
845.001-.001-.	Bolboceratidae	*Odonteus armiger*		1		1
85-.014-.005-.	Scarabaeidae	*Onthophagus verticicornis*	31	62	18	27
85-.014-.008-.	Scarabaeidae	*Onthophagus ovatus*	13	35	10	19
85-.014-.017-.	Scarabaeidae	*Onthophagus fracticornis*	4	2	4	2
85-.014-.018-.	Scarabaeidae	*Onthophagus similis*		2		2
85-.014-.019-.	Scarabaeidae	*Onthophagus coenobita*	7	2	7	2
85-.019-.012-.	Scarabaeidae	*Aphodius rufipes*	12	10	9	10
85-.019-.014-.	Scarabaeidae	*Aphodius depressus*	69	63	35	33
85-.019-.022-.	Scarabaeidae	*Aphodius maculatus*	1	5	1	5
85-.019-.031-.	Scarabaeidae	*Aphodius sticticus*	125	124	54	43
85-.019-.044-.	Scarabaeidae	*Aphodius prodromus*	23	16	8	9
85-.019-.060-.	Scarabaeidae	*Aphodius fimetarius*	4	3	4	3
85-.019-.066-.	Scarabaeidae	*Aphodius ater*	4	1	4	1
85-.019-.069-.	Scarabaeidae	*Aphodius borealis*		1		1
85-.019-.076-.	Scarabaeidae	*Aphodius rufus*	1		1	
85-.019-.079-.	Scarabaeidae	*Aphodius corvinus*	3	1	2	1
85-.019-.084-.	Scarabaeidae	*Aphodius varians*		1		1
85-.019-.086-.	Scarabaeidae	*Aphodius granarius*		1		1
85-.025-.001-.	Scarabaeidae	*Serica brunnea*	8	52	7	15
85-.033-.002-.	Scarabaeidae	*Melolontha melolontha*	1		1	
85-.037-.001-.	Scarabaeidae	*Phyllopertha horticola*	5	35	5	18
85-.040-.002-.	Scarabaeidae	*Hoplia philanthus*		1		1
85-.040-.005-.	Scarabaeidae	*Hoplia argentea*	1	17	1	6
85-.047-.0061.	Scarabaeidae	*Protaetia cuprea*	1	1	1	1
85-.048-.001-.	Scarabaeidae	*Valgus hemipterus*		24		8
85-.050-.001-.	Scarabaeidae	*Gnorimus nobilis*		1		1
85-.051-.001-.	Scarabaeidae	*Trichius fasciatus*	1	65	1	17
86-.001-.001-.	Lucanidae	*Lucanus cervus*		12		11
86-.002-.001-.	Lucanidae	*Dorcus parallelipipedus*	6	6	5	6
86-.003-.001-.	Lucanidae	*Platycerus caprea*		104		29
86-.003-.002-.	Lucanidae	*Platycerus caraboides*	185	112	63	36

86-.005-.001-.	Lucanidae	*Sinodendron cylindricum*	5	6	5	6
86-.006-.001-.	Lucanidae	*Aesalus scarabaeoides*	1		1	
87-.004-.001-.	Cerambycidae	*Prionus coriarius*	6	10	5	8
87-.006-.001-.	Cerambycidae	*Spondylis buprestoides*	1		1	
87-.008-.001-.	Cerambycidae	*Arhopalus rusticus*	2	1	2	1
87-.010-.001-.	Cerambycidae	*Tetropium castaneum*	6	10	5	8
87-.010-.002-.	Cerambycidae	*Tetropium fuscum*	2	1	2	1
87-.010-.003-.	Cerambycidae	*Tetropium gabrieli*	1		1	
87-.011-.001-.	Cerambycidae	*Rhagium bifasciatum*	329	599	57	51
87-.011-.002-.	Cerambycidae	*Rhagium sycophanta*	7	7	7	4
87-.011-.003-.	Cerambycidae	*Rhagium mordax*	52	200	38	64
87-.011-.004-.	Cerambycidae	*Rhagium inquisitor*	3	9	3	7
87-.014-.001-.	Cerambycidae	*Oxymirus cursor*	41	37	20	20
87-.015-.001-.	Cerambycidae	*Stenocorus meridianus*	7	18	5	13
87-.018-.003-.	Cerambycidae	*Evodinus clathratus*	23	24	12	17
87-.019-.001-.	Cerambycidae	*Gaurotes virginea*		2		2
87-.0201.001-.	Cerambycidae	*Dinoptera collaris*		16		7
87-.021-.001-.	Cerambycidae	*Pidonia lurida*		17		10
87-.022-.001-.	Cerambycidae	*Cortodera femorata*		1		1
87-.022-.002-.	Cerambycidae	*Cortodera humeralis*	2		2	
87-.023-.002-.	Cerambycidae	*Grammoptera ruficornis*		8		6
87-.024-.001-.	Cerambycidae	*Alosterna tabacicolor*	9	56	7	19
87-.027-.0021.	Cerambycidae	*Leptura aurulenta*		1		1
87-.027-.0031.	Cerambycidae	*Leptura quadrifasciata*	3	6	3	5
87-.0271.002-.	Cerambycidae	*Anoplodera sexguttata*	1	9	1	8
87-.0272.001-.	Cerambycidae	*Pseudovadonia livida*		2		2
87-.0274.004-.	Cerambycidae	*Stictoleptura maculicornis*		15		10
87-.0274.006-.	Cerambycidae	*Stictoleptura rubra*	36	68	21	39
87-.0274.009-.	Cerambycidae	*Stictoleptura scutellata*	1	3	1	3
87-.0275.001-.	Cerambycidae	*Anastrangalia sanguinolenta*		2		1
87-.0276.001-.	Cerambycidae	*Lepturobosca virens*		2		1
87-.0278.001-.	Cerambycidae	*Rutpela maculata*	11	74	11	30
87-.028-.001-.	Cerambycidae	*Judolia sexmaculata*		4		3
87-.0281.001-.	Cerambycidae	*Pachytodes cerambyciformis*	6	33	2	16
87-.0291.001-.	Cerambycidae	*Pedostrangalia revestita*		1		1
87-.0293.001-.	Cerambycidae	*Stenurella melanura*	29	758	19	72
87-.031-.001-.	Cerambycidae	*Saphanus piceus*		1		1
87-.037-.002-.	Cerambycidae	*Obrium brunneum*	1		1	
87-.039-.001-.	Cerambycidae	*Molorchus minor*	5	4	4	4
87-.053-.002-.	Cerambycidae	*Callidium violaceum*		1		1
87-.053-.003-.	Cerambycidae	*Callidium aeneum*	3		3	
87-.054-.001-.	Cerambycidae	*Pyrrhidium sanguineum*	3	3	3	3
87-.055-.001-.	Cerambycidae	*Phymatodes testaceus*	4	4	4	4

87-.058-.003-.	Cerambycidae	*Clytus arietis*	10	21	10	18
87-.063-.001-.	Cerambycidae	*Anaglyptus mysticus*	4	3	4	3
87-.075-.001-.	Cerambycidae	*Pogonocherus hispidulus*		2		2
87-.078-.001-.	Cerambycidae	*Leiopus nebulosus*	42	28	28	16
87-.078-.003-.	Cerambycidae	*Leiopus femoratus*	1		1	
87-.081-.003-.	Cerambycidae	*Agapanthia villosoviridescens*		6		3
87-.085-.001-.	Cerambycidae	*Stenostola dubia*	1	4	1	4
87-.086-.008-.	Cerambycidae	*Phytoecia cylindrica*		2		2
88-.0061.003-.	Chrysomelidae	*Oulema gallaeciana*	4	8	4	8
88-.0061.005-.	Chrysomelidae	*Oulema melanopus*	1	2	1	2
88-.008-.002-.	Chrysomelidae	*Lilioceris merdigera*		3		3
88-.008-.0021.	Chrysomelidae	*Lilioceris schneideri*		1		1
88-.017-.026-.	Chrysomelidae	*Cryptocephalus sericeus*		1		1
88-.017-.063-.	Chrysomelidae	*Cryptocephalus pygmaeus*		1		1
88-.023-.028-.	Chrysomelidae	*Chrysolina sturmi*		1		1
88-.023-.036-.	Chrysomelidae	*Chrysolina varians*		2		2
88-.023-.038-.	Chrysomelidae	*Chrysolina hyperici*		1		1
88-.023-.044-.	Chrysomelidae	*Chrysolina marginata*		1		1
88-.035-.012-.	Chrysomelidae	*Gonioctena intermedia*	1		1	
88-.036-.004-.	Chrysomelidae	*Phratora laticollis*	1	2	1	1
88-.041-.001-.	Chrysomelidae	*Galeruca tanaceti*	1	1	1	1
88-.042-.001-.	Chrysomelidae	*Lochmaea capreae*		2		2
88-.0451.002-.	Chrysomelidae	*Calomicrus pinicola*	1		1	
88-.049-.002-.	Chrysomelidae	*Phyllotreta vittula*	4	8	4	8
88-.049-.005-.	Chrysomelidae	*Phyllotreta undulata*		1		1
88-.049-.010-.	Chrysomelidae	*Phyllotreta striolata*		4		1
88-.049-.011-.	Chrysomelidae	*Phyllotreta ochripes*	3	4	3	4
88-.049-.014-.	Chrysomelidae	*Phyllotreta atra*		1		1
88-.049-.015-.	Chrysomelidae	*Phyllotreta cruciferae*		1		1
88-.049-.021-.	Chrysomelidae	*Phyllotreta nigripes*		1		1
88-.050-.014-.	Chrysomelidae	*Aphthona venustula*		1		1
88-.050-.015-.	Chrysomelidae	*Aphthona euphorbiae*	2	1	2	1
88-.051-.017-.	Chrysomelidae	*Longitarsus melanocephalus*	1	1	1	1
88-.051-.0171.	Chrysomelidae	*Longitarsus kutscherai*		1		1
88-.051-.019-.	Chrysomelidae	*Longitarsus exsoletus*		1		1
88-.051-.035-.	Chrysomelidae	*Longitarsus dorsalis*	1		1	
88-.051-.039-.	Chrysomelidae	*Longitarsus luridus*	9	43	6	17
88-.052-.004-.	Chrysomelidae	*Altica brevicollis*	5	2	4	2
88-.053-.002-.	Chrysomelidae	*Hermaeophaga mercurialis*	67	52	19	23
88-.054-.002-.	Chrysomelidae	*Batophila rubi*	2	15	2	3
88-.059-.001-.	Chrysomelidae	*Derocrepis rufipes*	2	6	2	5
88-.062-.001-.	Chrysomelidae	*Epitrix atropae*		3		1
88-.066-.003-.	Chrysomelidae	*Chaetocnema concinna*	3	19	3	8
88-.066-.004-.	Chrysomelidae	*Chaetocnema picipes*		1		1
88-.066-.017-.	Chrysomelidae	*Chaetocnema hortensis*		6		6
88-.069-.003-.	Chrysomelidae	*Apteropeda orbiculata*		3		2

88-.072-.002-.	Chrysomelidae	*Psylliodes affinis*		1		1
88-.072-.007-.	Chrysomelidae	*Psylliodes chrysocephala*	1	3	1	3
88-.072-.010-.	Chrysomelidae	*Psylliodes napi*	1	16	1	7
88-.076-.005-.	Chrysomelidae	*Cassida nebulosa*		1		1
88-.076-.028-.	Chrysomelidae	*Cassida vittata*		1		1
881.004-.001-.	Orsodacnidae	*Orsodacne cerasi*		2		2
89-.003-.004-.	Chrysomelidae	*Bruchus atomarius*		3		3
89-.003-.011-.	Chrysomelidae	*Bruchus brachialis*		1		1
89-.003-.014-.	Chrysomelidae	*Bruchus luteicornis*		1		1
89-.004-.010-.	Chrysomelidae	*Bruchidius varius*		2		1
90-.001-.001-.	Anthribidae	*Platyrhinus resinosus*	2	6	2	6
90-.008-.001-.	Anthribidae	*Dissoleucas niveirostris*	7	7	7	7
90-.010-.001-.	Anthribidae	*Platystomos albinus*	9	13	7	9
90-.012-.003-.	Anthribidae	*Anthribus nebulosus*	5	4	4	4
90-.015-.002-.	Anthribidae	*Choragus sheppardi*		1		1
91-.001-.003-.	Curculionidae	*Scolytus intricatus*	3	11	3	7
91-.001-.004-.	Curculionidae	*Scolytus mali*		1		1
91-.001-.005-.	Curculionidae	*Scolytus carpini*	1	2	1	2
91-.0031.003-.	Curculionidae	*Phloeotribus spinulosus*	1		1	
91-.004-.001-.	Curculionidae	*Hylastes ater*	4		4	
91-.004-.0011.	Curculionidae	*Hylastes brunneus*		2		2
91-.004-.002-.	Curculionidae	*Hylastes opacus*	1		1	
91-.004-.003-.	Curculionidae	*Hylastes cunicularius*	419	222	76	51
91-.004-.005-.	Curculionidae	*Hylastes attenuatus*	43	20	20	10
91-.005-.001-.	Curculionidae	*Hylurgops glabratus*		1		1
91-.005-.002-.	Curculionidae	*Hylurgops palliatus*	48	35	21	20
91-.006-.002-.	Curculionidae	*Tomicus piniperda*	1		1	
91-.010-.002-.	Curculionidae	*Polygraphus poligraphus*	7	2	6	2
91-.011-.001-.	Curculionidae	*Hylesinus crenatus*		1		1
91-.011-.003-.	Curculionidae	*Hylesinus varius*	1	10	1	7
91-.011-.004-.	Curculionidae	*Hylesinus wachtli*		8		8
91-.013-.001-.	Curculionidae	*Hylastinus obscurus*		2		2
91-.016-.001-.	Curculionidae	*Xylechinus pilosus*	40	8	19	4
91-.020-.001-.	Curculionidae	*Crypturgus cinereus*	18	15	11	12
91-.020-.002-.	Curculionidae	*Crypturgus hispidulus*		28		15
91-.020-.003-.	Curculionidae	*Crypturgus pusillus*	37	2	17	2
91-.021-.001-.	Curculionidae	*Lymantor coryli*	1		1	
91-.022-.001-.	Curculionidae	*Xylocleptes bispinus*		2		2
91-.024-.001-.	Curculionidae	*Dryocoetes autographus*	98	78	42	34
91-.024-.0011.	Curculionidae	*Dryocoetes hectographus*		6		5
91-.024-.002-.	Curculionidae	*Dryocoetes villosus*	9	2	3	2
91-.024-.003-.	Curculionidae	*Dryocoetes alni*		1		1
91-.025-.002-.	Curculionidae	*Trypophloeus granulatus*		1		1
91-.026-.001-.	Curculionidae	*Cryphalus piceae*		2		2
91-.026-.004-.	Curculionidae	*Cryphalus asperatus*	21	7	15	6
91-.027-.001-.	Curculionidae	*Ernoporicus fagi*	247	80	62	43
91-.028-.001-.	Curculionidae	*Ernoporus tiliae*	1	1	1	1
91-.029-.001-.	Curculionidae	*Pityophthorus exsculptus*		1		1

91-.029-.002-.	Curculionidae	*Pityophthorus pityographus*	180	55	25	23
91-.031-.003-.	Curculionidae	*Taphrorychus bicolor*		52		28
91-.031-.004-.	Curculionidae	*Taphrorychus villifrons*	56	44	33	20
91-.032-.001-.	Curculionidae	*Pityogenes chalcographus*	85	59	36	22
91-.032-.006-.	Curculionidae	*Pityogenes bidentatus*	2		1	
91-.034-.002-.	Curculionidae	*Orthotomicus laricis*		1		1
91-.035-.004-.	Curculionidae	*Ips typographus*	73	55	18	25
91-.036-.003-.	Curculionidae	*Xyleborus cryptographus*	2		2	
91-.036-.005-.	Curculionidae	*Xyleborus monographus*	1	4	1	4
91-.036-.007-.	Curculionidae	*Xyleborus dryographus*	4	3	4	3
91-.0361.001-.	Curculionidae	*Cyclorhipidion bodoanum*	29	15	15	11
91-.0362.001-.	Curculionidae	*Anisandrus dispar*	218	94	44	24
91-.0363.001-.	Curculionidae	*Xyleborinus saxesenii*	544	307	47	44
91-.0364.001-.	Curculionidae	*Xylosandrus germanus*	2153	487	79	68
91-.038-.001-.	Curculionidae	*Trypodendron domesticum*	371	226	51	46
91-.038-.002-.	Curculionidae	*Trypodendron signatum*	112	24	39	15
91-.038-.003-.	Curculionidae	*Trypodendron lineatum*	3	34	1	19
91-.038-.004-.	Curculionidae	*Trypodendron laeve*	2		2	
92-.001-.001-.	Curculionidae	*Platypus cylindrus*		1		1
922.002-.001-.	Nemonychidae	*Cimberis attelaboides*	15	14	12	12
923.003-.003-.	Rhynchitidae	*Lasiorhynchites olivaceus*	50	20	23	13
923.0041.001-.	Rhynchitidae	*Neocoenorrhinus germanicus*		3		3
923.0042.001-.	Rhynchitidae	*Tatianaerhynchites aequatus*		2		2
923.007-.004-.	Rhynchitidae	*Deporaus betulae*	1	2	1	1
925.001-.004-.	Apionidae	*Omphalapion hookerorum*		1		1
925.021-.002-.	Apionidae	*Protapion fulvipes*	1	5	1	5
925.021-.003-.	Apionidae	*Protapion nigritarse*		1		1
925.021-.004-.	Apionidae	*Protapion filirostre*		1		1
925.021-.005-.	Apionidae	*Protapion trifolii*	1	3	1	3
925.021-.008-.	Apionidae	*Protapion apricans*		4		2
925.036-.001-.	Apionidae	*Synapion ebeninum*		1		1
925.038-.001-.	Apionidae	*Hemitrichapion reflexum*		1		1
925.042-.003-.	Apionidae	*Oxystoma craccae*	2	1	1	1
925.042-.007-.	Apionidae	*Oxystoma ochropus*		4		4
925.044-.006-.	Apionidae	*Eutrichapion punctiger*		1		1

93-.015-.041-.	Curculionidae	*Otiorhynchus tenebricosus*	10	1	6	1
93-.015-.057-.	Curculionidae	*Otiorhynchus nodosus*	2		2	
93-.015-.060-.	Curculionidae	*Otiorhynchus rugosostriatus*		7		3
93-.015-.085-.	Curculionidae	*Otiorhynchus porcatus*		1		1
93-.015-.089-.	Curculionidae	*Otiorhynchus carinatopunctatus*	117	132	28	32
93-.015-.104-.	Curculionidae	*Otiorhynchus singularis*	80	25	31	13
93-.015-.108-.	Curculionidae	*Otiorhynchus pupillatus*		2		2
93-.015-.159-.	Curculionidae	*Otiorhynchus ovatus*		1		1
93-.015-.162-.	Curculionidae	*Otiorhynchus crataegi*		1		1
93-.018-.001-.	Curculionidae	*Simo hirticornis*	19	14	8	9
93-.021-.008-.	Curculionidae	*Phyllobius oblongus*	27	28	8	13
93-.021-.013-.	Curculionidae	*Phyllobius arborator*	5	10	4	7
93-.021-.014-.	Curculionidae	*Phyllobius pomaceus*	1		1	
93-.021-.015-.	Curculionidae	*Phyllobius glaucus*		3		3
93-.021-.019-.	Curculionidae	*Phyllobius argentatus*	207	128	48	23
93-.025-.001-.	Curculionidae	*Rhinomias forticornis*	356	86	43	17
93-.0264.002-.	Curculionidae	*Cathormiocerus spinosus*	4		4	
93-.027-.001-.	Curculionidae	*Polydrusus impar*	3		3	
93-.027-.003-.	Curculionidae	*Polydrusus aeratus*		2		2
93-.027-.007-.	Curculionidae	*Polydrusus pterygomalis*	76	211	24	34
93-.027-.016-.	Curculionidae	*Polydrusus tereticollis*	118	112	37	36
93-.027-.023-.	Curculionidae	*Polydrusus formosus*	19	6	11	5
93-.027-.026-.	Curculionidae	*Polydrusus mollis*	5	17	2	11
93-.029-.001-.	Curculionidae	*Liophloeus tessulatus*		4		3
93-.033-.001-.	Curculionidae	*Sciaphilus asperatus*	6	2	5	2
93-.035-.003-.	Curculionidae	*Brachysomus hirtus*	13	23	9	15
93-.035-.006-.	Curculionidae	*Brachysomus echinatus*	3		2	
93-.037-.002-.	Curculionidae	*Exomias tenex*	293	60	9	9
93-.037-.007-.	Curculionidae	*Exomias araneiformis*	180	155	23	20
93-.037-.011-.	Curculionidae	*Exomias pellucidus*	386	941	29	54
93-.040-.002-.	Curculionidae	*Strophosoma melanogrammum*	2337	1868	75	71
93-.040-.003-.	Curculionidae	*Strophosoma capitatum*	219	116	15	10
93-.043-.002-.	Curculionidae	*Barynotus obscurus*	2	15	2	12
93-.043-.003-.	Curculionidae	*Barynotus moerens*		5		3
93-.043-.004-.	Curculionidae	*Barynotus alternans*	3	2	2	2
93-.044-.007-.	Curculionidae	*Sitona striatellus*	1		1	
93-.044-.010-.	Curculionidae	*Sitona lineatus*	1	14	1	6
93-.044-.013-.	Curculionidae	*Sitona sulcifrons*		1		1
93-.044-.016-.	Curculionidae	*Sitona obsoletus*		1		1
93-.044-.024-.	Curculionidae	*Sitona humeralis*	1	3	1	2
93-.050-.004-.	Curculionidae	*Tropiphorus elevatus*	7	4	7	4
93-.051-.016-.	Curculionidae	*Lixus fasciculatus*		2		2
93-.069-.001-.	Curculionidae	*Cotaster uncipes*	3		3	
93-.078-.004-.	Curculionidae	*Rhyncolus ater*	9	8	9	8
93-.081-.001-.	Curculionidae	*Stereocorynes truncorum*	1		1	
93-.090-.020-.	Curculionidae	*Dorytomus rufatus*	1		1	
93-.099-.001-.	Curculionidae	*Orthochaetes setiger*	1		1	
93-.102-.001-.	Curculionidae	*Ellescus scanicus*	1		1	

93-.103-.001-.	Curculionidae	*Lignyodes enucleator*		1		1
93-.104-.019-.	Curculionidae	*Tychius picirostris*	5	5	4	5
93-.106-.010-.	Curculionidae	*Anthonomus pedicularius*		1		1
93-.106-.015-.	Curculionidae	*Anthonomus rubi*		5		5
93-.106-.017-.	Curculionidae	*Anthonomus phyllocola*	17	22	9	7
93-.106-.021-.	Curculionidae	*Anthonomus rectirostris*	26	1	11	1
93-.108-.001-.	Curculionidae	*Brachonyx pineti*	2		2	
93-.109-.001-.	Curculionidae	*Bradybatus creutzeri*		1		1
93-.109-.004-.	Curculionidae	*Bradybatus kellneri*	25	7	7	6
93-.109-.006-.	Curculionidae	*Bradybatus fallax*		1		1
93-.110-.002-.	Curculionidae	*Curculio venosus*	8	3	7	3
93-.110-.006-.	Curculionidae	*Curculio glandium*	21	6	16	6
93-.1101.011-.	Curculionidae	*Archarius pyrrhoceras*	2		2	
93-.111-.001-.	Curculionidae	*Pissodes piceae*		1		1
93-.111-.006-.	Curculionidae	*Pissodes pini*		1		1
93-.112-.002-.	Curculionidae	*Magdalis ruficornis*	1		1	
93-.112-.006-.	Curculionidae	*Magdalis cerasi*	1	2	1	2
93-.112-.013-.	Curculionidae	*Magdalis nitida*		1		1
93-.112-.017-.	Curculionidae	*Magdalis violacea*	3		3	
93-.113-.001-.	Curculionidae	*Trachodes hispidus*	11	8	10	8
93-.115-.001-.	Curculionidae	*Hylobius excavatus*	1		1	
93-.115-.002-.	Curculionidae	*Hylobius abietis*	9	34	9	8
93-.116-.007-.	Curculionidae	*Liparus coronatus*		7		5
93-.117-.001-.	Curculionidae	*Leiosoma deflexum*	7	29	6	8
93-.120-.001-.	Curculionidae	*Mitoplinthus caliginosus*	10	1	6	1
93-.123-.003-.	Curculionidae	*Graptus triguttatus*		1		1
93-.125-.014-.	Curculionidae	*Hypera meles*		1		1
93-.125-.024-.	Curculionidae	*Hypera postica*	2	5	2	5
93-.125-.030-.	Curculionidae	*Hypera nigrirostris*	2	2	2	2
93-.132-.001-.	Curculionidae	*Gasterocercus depressirostris*		1		1
93-.135-.007-.	Curculionidae	*Acalles camelus*	102	60	32	26
93-.135-.013-.	Curculionidae	*Acalles fallax*	36	14	19	12
93-.135-.014-.	Curculionidae	*Acalles micros*	25	12	9	8
93-.1351.001-.	Curculionidae	*Kyklioacalles aubei*	1	3	1	3
93-.1351.002-.	Curculionidae	*Kyklioacalles roboris*	3	9	2	5
93-.1352.003-.	Curculionidae	*Echinodera hypocrita*	14	10	14	10
93-.1373.003-.	Curculionidae	*Aulacobaris coerulescens*	1		1	
93-.145-.008-.	Curculionidae	*Rhinoncus castor*		1		1
93-.157-.003-.	Curculionidae	*Coeliodes rana*	4	2	4	2
93-.163-.003-.	Curculionidae	*Ceutorhynchus erysimi*	1		1	
93-.163-.023-.	Curculionidae	*Ceutorhynchus pallidactylus*	5	2	4	2
93-.163-.024-.	Curculionidae	*Ceutorhynchus atomus*	5	4	5	4
93-.163-.040-.	Curculionidae	*Ceutorhynchus obstrictus*	2	3	2	3
93-.163-.0601.	Curculionidae	*Ceutorhynchus typhae*	2	12	1	7
93-.169-.001-.	Curculionidae	*Nedyus quadrimaculatus*		5		2

93-.173-.011-.	Curculionidae	*Mecinus pascuorum*	2	2	2	2
93-.1741.004-.	Curculionidae	*Rhinusa antirrhini*		1		1
93-.176-.002-.	Curculionidae	*Cionus tuberculosus*	1	1	1	1
93-.177-.001-.	Curculionidae	*Cleopus solani*		1		1
93-.178-.001-.	Curculionidae	*Stereonychus fraxini*	9	8	5	7
93-.1802.004-.	Curculionidae	*Tachyerges salicis*		1		1
93-.1804.002-.	Curculionidae	*Orchestes betuleti*	1		1	
93-.1804.005-.	Curculionidae	*Orchestes quercus*		2		2
93-.1804.013-.	Curculionidae	*Orchestes fagi*	504	536	47	47
933.003-.001-.	Dryophthoridae	*Dryophthorus corticalis*	5	2	2	2

Table S3: Comparison of meta-analysis results and simple paired t-tests.

**Scale**	**Facet**	**Meta_LC**	**Meta_Dif**	**Meta_UC**	**t_Est**	**t_pval**
γ	TD	58.905	65.464	72.023	83.332	**0.001**
α	TD	28.477	32.728	36.979	31.247	**0.000**
β (1-S)	TD	0.091	0.101	0.112	0.092	**0.009**
γ	FD	-0.218	1.691	3.601	1.976	**0.000**
α	FD	0.568	1.031	1.494	1.100	**0.001**
β (1-S)	FD	-0.013	0.007	0.026	0.004	0.379

Additional references not present in main manuscript

Birkemoe, T., R. M. Jacobsen, A. Sverdrup-Thygeson, and P. H. Biedermann. 2018. “Insect-Fungus Interactions in Dead Wood Systems.” In Saproxylic Insects: Diversity, Ecology and Conservation, 377–427. Springer International Publishing.

Brändle, M., Brandl, R. (2001). Species richness of insects and mites on trees: expanding Southwood. J. Animal Ecology, 70, 491-504.

Bussler, H., Bouget, C., Brustel, H., Brändle, H., Riedinger, V., Brandl et al. (2011). Abundance and pest classification of scolytid species (Coleoptera: Curculionidae, Scolytinae) follow different patterns. Forest Ecology and Management, 262, 1887-1894.

Chown, S.L. & Gaston, K.J. (2010). Body size variation in insects: a macroecological perspective. Biological Reviews, 85, 139-169.

Gossner, M.M., Lachat, T., Brunet, J., Isacsson, G., Bouget, C., Brustel, H. et al. (2013). Current "near-to-nature" forest management effects on functional trait composition of saproxylic beetles in beech forests. Conserv. Biol., 27, 605-614.

Guzman, L.M., Thompson, P.L., Viana, D.S., Vanschoenwinkel, B., Horvath, Z., Ptacnik, R. et al. (2022). Accounting for temporal change in multiple biodiversity patterns improves the inference of metacommunity processes. Ecology, 103, e3683.

Kirkendall, L., Biedermann, P., Jordal, B. (2015). Evolution and Diversity of Bark and Ambrosia Beetles. Pages 85-156 in F. Vega and R. Hofstetter, editors. Bark Beetles: Biology and Ecology of Native and Invasive Species. Academic Press, San Diego.

Kortmann, M., Chao, A., Chiu, C.-H., Heibl, C., Mitesser, O., Morinière, J. et al. (2025a). A short cut to sample coverage standardization in meta-barcoding data provides new insights into land use effects on insect diversity. Proc R Soc Lond B Biol Sci, 292, 20242927.

Kortmann, M., Chao, A., Schaefer, H.M., Blüthgen, N., Gelis, R., Tremlett, C.J. et al. (2025b). Sample coverage affects diversity measures of bird communities along a natural recovery gradient of abandoned agriculture in tropical lowland forests. Journal of Applied Ecology, 62, 480-491.

Mori, A.S., Fujii, S., Kitagawa, R. & Koide, D. (2015a). Null model approaches to evaluating the relative role of different assembly processes in shaping ecological communities. Oecologia, 178, 261-273.

Müller, J., Hothorn, T., Yuan, Y., Seibold, S., Mitesser, O., Rothacher, J. et al. (2024). Weather explains the decline and rise of insect biomass over 34 years. Nature, 628, 349-354.

Rothacher, J., Seidl, R., Thom, D., Kortmann, M., Chao, A., Chiu, C.-H. et al. (2025). The impact of tree mortality and post-disturbance management on insect diversity in temperate forests: Insights from a replicated experiment. Journal of Applied Ecology, 62 1878-1888.

Seibold, S., Müller, J., Franz, K., Baldrian, P., Cadotte, M.W., Brandl, R. et al. (2019). Fungi associated with beetles dispersing from dead wood – Let's take the beetle bus! Fungal Ecology, 39, 100-108.

van Klink, R., Bowler, D.E., Gongalsky, K.B., Shen, M., Swengel, S.R. & Chase, J.M. (2024). Disproportionate declines of formerly abundant species underlie insect loss. Nature, 628, 359–364.

Vogel, S., H. Bussler, S. Finnberg, J. Müller, E. Stengel, Thorn, S. (2021). Diversity and conservation of saproxylic beetles of 42 European tree species – an experimental approach from early successional stages of branches. Insect Conserv Divers, 14, 132-143.

Weslien, J., Djupström, L.B., Schroeder, M., Widenfalk, O. (2011). Long-term priority effects among insects and fungi colonizing decaying wood. Journal of Animal Ecology, 80, 1155-1162.

Zeuss, D., Brandl, R., Brandle, M., Rahbek, C. & Brunzel, S. (2014). Global warming favours light-coloured insects in Europe. Nature Communications, 5, 3874.

Zou, J.Y., Cadotte, M.W., Bässler, C., Brandl, R., Baldrian, P., Borken, W. et al. (2023). Wood decomposition is increased by insect diversity, selection effects and interactions between insects and microbes. Ecology, 104, e4184.
